# Incidence and Complications of Atrial Fibrillation in a Low Socioeconomic and High Disability United States (US) Population: A Combined Statistical and Machine Learning Approach

**DOI:** 10.1155/2022/8649050

**Published:** 2022-08-30

**Authors:** Gregory Y. H. Lip, Ash Genaidy, George Tran, Patricia Marroquin, Cara Estes

**Affiliations:** ^1^Liverpool Centre for Cardiovascular Science, University of Liverpool and Liverpool Heart & Chest Hospital, Liverpool, UK; ^2^Anthem Inc, Indianapolis, IN, USA; ^3^IngenioRX, Indianapolis, IN, USA

## Abstract

**Background:**

Poor socioeconomic status coupled with individual disability is significantly associated with incident atrial fibrillation (AF) and AF-related adverse outcomes, with the information currently lacking for US cohorts. We examined AF incidence/complications and the dynamic nature of associated risk factors in a large socially disadvantaged US population.

**Methods:**

A large population representing a combined poor socioeconomic status/disability (Medicaid program) was examined from diverse geographical regions across the US continent. The target population was extracted from administrative databases with patients possessing medical/pharmacy benefits. This retrospective cohort study was conducted from Jan 1, 2016, to Sep 30, 2021, and was limited to 18- to 80-year age group drawn from the Medicaid program. Descriptive and inferential statistics (parametric: logistic regression and neural network) were applied to all computations using a combined statistical and machine learning (ML) approach.

**Results:**

A total of 617413 individuals participated in the study, with mean age of 41.7 years (standard deviation “SD” 15.2) and 65.6% female patients. Seven distinct groups were identified with different combinations of low socioeconomic status and disability constraints. The overall crude AF incidence rate was 0.49 cases/100 person-years (95% confidence limit “CI” 0.40–0.58), with the lowest rate for the younger group (temporary assistance for needy family “TANF”) (0.20, 95%CI 0.18–0.21), the highest rates for the older groups (age, blindness, or disability “ABD” duals—1.51, 95% CI 1.31–1.58; long-term services and support “LTSS” duals—1.45, 95% CI 1.31–1.58), and the remaining four other groups in between the lower and upper rates. Based on independent effects after accounting for confounders in main effect modeling, the point estimates of odds ratios for AF status with various clinical outcomes were as follows: stroke (2.69, 95% CI 2.53–2.85); heart failure (6.18, 95% CI 5.86–6.52); myocardial infarction (3.71, 95% CI 3.49–3.94); major bleeding (2.26, 95% CI 2.14–2.38); and cognitive impairment (1.74, 95% CI 1.59–1.91). A logistic regression-based ML model produced excellent discriminant validity for high-risk AF outcomes (c “concordance” index based on training data 0.91, 95%CI 0.891–0.929), together with similar measures for external validity, calibration, and clinical utility. The performance measures for the ML models predicting associated complications with high-risk AF cases were good to excellent.

**Conclusions:**

A combination of low socioeconomic status and disability contributes to AF incidence and complications, elevating risks to higher levels relative to the general population. ML algorithms can be used to identify AF patients at high risk of clinical events. While further research is definitely in need on this socially important issue, the reported investigation is unique in which it integrates the general case about the subject due to the different ethnic groups around the world under a unified culture stemming from residing in the US.

## 1. Introduction

Atrial fibrillation (AF) is an evolving medical condition that is fueled in most cases by cardiovascular and/or noncardiovascular multimorbidity. [1, 2] If not properly treated, AF patients are at a high risk of stroke and other AF-related complications related to dynamic interactions with age, gender, and associated comorbidities [3].

In addition to the traditional cardiovascular risk factors, poor socioeconomic status coupled with individual disability are significantly associated with adverse AF outcomes. [4, 5] Yet, there is little information in the published literature from the US. [6] A recent study [7] in the US indicated that individuals with household income <^$^40 000 had the greatest risk for heart failure (HR “hazard ratio” 1.17; 95% CI 1.05 to 1.30) and MI (HR 1.18; 95% CI 0.98 to 1.41) relative to those with income ≥^$^100 000.

In the US, the Medicaid population is a typical program for multiple groups with varying characteristics financed by the federal/state governments, with individuals centered around the poverty line and several constituents possessing varied degrees of cognitive and/or physical disability. To date, there has been no study examining these different groups within the Medicaid space with respect to AF incidence and associated complications.

This investigation was initiated to examine this issue in greater detail, with an emphasis on the incident AF and AF-related complications, as well as the dynamic nature of associated risk factors in a large socially disadvantaged Medicaid population, spread across several geographical areas in the US continent.

Our specific aims are as follows: (i) to report the incidence of AF in Medicaid recipients making up seven distinct groups of low socioeconomic status and disability; and (ii) to use a combined statistical and machine learning (ML) approach to examine and predict AF incidence and AF-related complications (i.e., stroke, congestive heart failure, myocardial infarction, major bleeding, and cognitive impairment) as a function of different Medicaid groups and their comorbid/demographic profiles.

## 2. Methods

### 2.1. Data Sources and Eligibility Criteria for Poor or Socially Disadvantaged Socioeconomic Status

The patients were drawn from the US Medicaid program financed by both the federal and state governments. The program is aimed to address healthcare coverage for the adult population ranging in age from 18 to 90 years and characterized with low socioeconomic status coupled with disability. The data were accessed from administrative medical and pharmacy claim databases that are subject to US privacy laws.

This retrospective cohort study period was conducted from Jan 1, 2016, to Sep 30, 2021, with patients enrolled in both medical and pharmacy benefits. This population was subjected to a number of inclusions and exclusions with the most notable of continuous enrollment with a minimum period of 30 months to allow the investigation of incident AF cases which require the absence of claims for at least 24 months with AF ICD (“International Classification of Diseases”) 10 codes in the medical databases.

Our approach was in line with the methods determined by Piccini et al. [8] and Lip et al. [2] According to the researchers, to avoid classifying patients with prevalent AF as incident cases, it was considered that an individual is to have an incident AF only if the diagnosis occurred after at least 2 years of enrollment in the health plan with no AF diagnosis. Tu et al. [9] and Lip et al. [2] found that adding additional requirements based on pharmacy data would increase the sensitivity and specificity of identifying incident AF patients from administrative databases. The researchers reported the added requirements of the absence of anticoagulant use and heart rhythm control subject to the proper exclusions (see suppl Tables S1 and S2).

### 2.2. Definition of Poor Economic Status/Socially Disadvantaged for Eligibility into Medicaid Programs and Its Group Categories

Eligibility into Medicaid programs is based on financial requirements and/or medical needs. The upper limit for financial criteria is based on a percentage of the Federal Poverty Line (FPL), which is considered a measure of the minimum household income developed yearly by the US government. For 2021, the federal government set the income limit to qualify for Medicaid at 138% of the FPL depending on the family size. For example, if a two-person household's income is at or less than 138% of ^$^17,420–^$^24,040 annually or ^$^2,003 per month, one would be eligible for government assistance programs. For a three-member household, the upper limit is ^$^2,525 per month. The medically needy program varies from state to state and is designed for individuals with significant health needs whose income is higher than the income limit requirements to otherwise qualify for Medicaid under income eligibility groups. As such, the main criteria for eligibility for Medicaid are based on financial and medical needs and are reserved for individuals with poor economic status and critical medical needs.

### 2.3. The Medicaid Program Have Several Groups Including

- Temporary assistance for needy families (TANF)—This program, which is time limited, assists families with children when the parents or other responsible relatives cannot provide for the family's basic needs.
- Family care–This Medicaid program offers healthcare coverage focused on uninsured adults and parents of Medicaid-eligible children and parents of CHIP (i.e., children's health insurance program, that is, insurance program providing low-cost health coverage to children in families that earn too much money to qualify for Medicaid but not enough to buy private insurance).
- Age, blindness, or disability (ABD)—This program is open to individuals who are eligible for and receive Medicaid benefits because of age, blindness, or disability in addition to the amount of their income and assets. These patients are characterized as nondual Medicaid recipients with the exception of a subset group of patients who are termed as dual eligible with benefits paid for by both Medicaid and Medicare (each patient is designated as primary Medicare and secondary Medicaid).
- Long-term services and support (LTSS)—It is a diverse group, extending from young to old adult population, with many different types of physical and cognitive disabilities and receiving (a) institutional care and (b) home- and community-based services. They often receive services and support for many years, or even decades, and often have complex conditions and high needs. Thus, they are among the Medicaid's most expensive beneficiaries. Similar to the ABD program, individuals in the LTSS group also have a subset of a dual-eligible population.

With the above in mind, the TANF, family care, nondual ABD, or nondual LTSS programs are covered by Medicaid. On the other hand, individuals in the dual ABD and LTSS programs are covered by both Medicaid and Medicare.

### 2.4. Variable Definition

The index date for an incident AF case was qualified as the date corresponding to the first medical claim with an AF ICD 10 code as explained above. The incidence of any adverse clinical outcome (i.e., heart failure, stroke, myocardial infarction, major bleeding, or cognitive impairment) was identified as the first case, after the AF index date by at least 30 days until the end of the study period (Sep 30, 2021) (see suppl.  Table S3 for the definition of outcomes). Patients were censored for each of the five adverse clinical outcomes. Clinical outcomes were treated as binary variables with 1 for the presence of a condition and 0 for its absence. The presence or absence of AF was also treated as a binary variable and was defined in a similar way.

The list of comorbid conditions was identified during a baseline period of 2 years preceding the AF index date. The clinical outcomes and baseline comorbid conditions were identified from medical claims using primary and/or secondary diagnoses, as summarized in suppl Table S3 (for ICD 10 codes). Each comorbid condition was treated as a binary variable, with 1 for condition presence and 0 for its absence.

Demographic variables included gender and age. Gender was used as a binary variable, with females as 1 and males as 0 (or reference group). Age was defined as a continuous (in years) as well as a categorical (i.e., nominal variable consisting of multiple levels) variable, with several groups (18–44 years being the reference group or 0; 45–54 years or 1; 55–64 years or 2; 65–74 years or 3; and 75–90 years or 4).

Medicaid group type was categorized into seven groups (TANF or 0; family care or 1; any two categories enrolled at different times by the same patient or 2; ABD nondual or 3; LTSS nondual or 4; ABD duals or 5; and LTSS duals or 6). The TANF group was the reference category and the Medicaid group type was treated as a nominal variable, with multiple levels including the reference group.

Two multimorbid indices were defined in this study as the sum of comorbid conditions (i.e., the sum of all 1s when a chronic condition is present) for the first index and the sum of all comorbid conditions and age group as a nominal variable. The two multimorbid indices included both cardiovascular and noncardiovascular multimorbidity. The CHADS2, [10] CHA2DS2_VASc, [11] and C2HEST [12] clinical rules were used as originally defined in the literature.

### 2.5. Quantitative Analyses

The analyses included both descriptive and inferential computations. The SAS Enterprise Software was used for all descriptive computations and main effect modeling using logistic regression. Machine learning computations were performed using parametric methods (i.e., logistic regression and neural networking) of the SAS Miner Software [13, 14].

The details of quantitative analyses are provided in Supplementary Materials. In particular, in order to address the limitations of using administrative databases in reference to the severity of comorbid conditions and clinical outcomes, this was partly accomplished by using a cost threshold with the assumption that any clinical condition exceeding the cost threshold is considered economically costlier conditions and hence more severe. Therefore, a binary variable was created with 1 representing the high cost (hence, the severe cases) and 0 representing the lower cost (thus, the mild cases). The cost was based on the total allowed amount (paid by the insurance company as well as the deduction paid by the patient in a given health plan) for the year prior to the AF index date. Therefore, any member with, for example, a total annual cost of ^$^2000 or more in the prior year to start participating in the study (as defined by the index date or the equivalent) was considered a severe case or else nonsevere case or ^$^0. More details are provided in Appendix S1.

## 3. Results

### 3.1. Medicaid Population/Group Characteristics and Comorbid Profile

The final Medicaid population consisted of 617413 individuals (65.6% female) and average age of 41.7 years (SD 15.2) (Table 1). About 59.2% of the population consisted of the age 18–44 years bracket, followed by 19.8% and 14.6%, respectively, for the age 45–54 and 55–64 groups.

The TANF group was the largest and consisted of 50.9% of the Medicaid population. The LTSS duals and nondual groups were evenly distributed at 4.8% each. The ABD nondual was the second largest individual Medicaid group with a sample close to 11.9% of the Medicaid population; the ABD duals were much smaller and equal to 3.8%. The family care group was among the smallest individual cohorts in the Medicaid population (3.8%). Lastly, about 19.9% of the population was enrolled in two individual programs during their benefit enrollment.

With respect to age, TANF had the youngest age group (36.8 years SD 11.8) and the duals were the oldest (LTSS: 68.6 years SD 13.9; ABD: 56.2 years SD 15.3). Other groups were, on average, older than the TANF group within a range of 4 to 8 years.

Among the comorbid conditions, hypertension (35.9%), spondylosis/intervertebral discs (36.6%), and lipid disorders (27.6) had the highest prevalence, followed by a high prevalence of depression (19.2%) (Table 1).

### 3.2. Crude Incidence Rates for Medicaid Groups

Table 2 shows the details of crude incidence rate analyses. The TANF group had the lowest incidence rate in new cases/100 person-years (0.20 95% CI 0.18–0.21). The dual ABD or LTSS duals had the highest incidence rates (ABD—1.50 95% CI 1.31–1.58; LTSS—1.45 95% CI 1.31–1.58). Other groups had crude incidence rates between the TANF and ABD/LTSS duals (family care—0.79 95% CI 0.68–0.90; two groups—0.61 95% CI 0.57–0.66; LTSS nondual—0.61 95% CI 0.53–0.70; and ABD nondual—0.69 95% CI 0.63–0.75). The overall crude incidence rate was 0.49 (95% CI 0.40–0.58).

### 3.3. AF Status Outcome and AF Complication Outcomes Using Main Effect/ML Modeling

With care cost threshold introduced as a model feature (i.e., an input variable defined as a risk factor if its total annual cost prior to the index date for AF cases or equivalent for non AF cases exceeds the cost threshold of ^$^2000), all comorbid conditions were significant risk factors for incident AF events, with the exception of cognitive impairment which was nonstatistically significant, while lipid disorders, metabolic syndrome, and asthma were protective factors (Table 3). Males were at a higher risk of AF incidence than females; as well as advancing in age. In general, strong risk factors (≥50% higher risk for AF incidence relative to its absence) were congestive heart failure, hypertension, valvular disease, chronic obstructive pulmonary disease, cost threshold, age group, and Medicaid group type. The C index was 0.822.

The ML models demonstrated better discriminant validity relative to main effect modeling. The two parametric methods employed showed comparative results for both: (a) training (logistic regression–c index 0.856; 95% CI 0.832–0.88; neural network–c index 0.846; 95% CI 0.845–0.847); and (b) validation (logistic regression–c index 0.851; 95% CI 0.816–0.886; neural network–c index 0.844; 95% CI 0.803–0.885) (suppl Figure S1). The true value of ML models lies in their nonlinear associations with the outcomes including the two-way interactions (suppl Table S4).

The point estimates of odds ratios for AF status with various clinical outcomes were as follows: stroke (2.69 95% CI 2.53–2.85); heart failure (6.18 95% CI 5.86–6.52); myocardial infarction (3.71 95% CI 3.49–3.94); major bleeding (2.26 95% CI 2.14–2.38); and cognitive impairment (1.74 95% CI 1.59–1.91) (Table 4).

The Medicaid group type significantly contributed to the adverse effects of clinical outcomes relative to the TANF referent group. The ABD nondual and LTSS nondual had the strongest associations with the clinical outcomes using main effect modeling. As expected, a prior history of an adverse clinical outcome was a major contributor to its future events, with the risk effects varying from almost 3 to 20 times (prior major bleeding: 3.79 (95% CI 3.69–3.89); prior myocardial infarction: 3.36 (95% CI 3.22–3.51); prior stroke: 9.86 (95% CI 9.49–10.25); prior heart failure: 18.23 (95% CI 17.53–18.95); prior cognitive impairment: 20.24 (95% CI 18.97–21.59)) (Table 4).

### 3.4. ML Modeling of Higher Risk AF Incidence and Associated Complications

Table 5 shows the c index values for the ML-based models for higher-risk AF incidence (defined by condition presence and cost threshold of at least ^$^5000 in terms of total care cost in the year prior to index date) and the associated adverse clinical outcomes. The *c* index values were good to excellent (0.82–0.92 for all outcomes, except for major bleeding which was about average “0.72”) for both the training and validation samples. The areas under the curve and the curve calibration for the external validation samples were good (suppl Figure S2).

The cumulative lift values were good (Table 5). For example, targeting the top 10% of high-risk populations would capture about 70% of all AF patients, and 50% to 65% of the associated stroke, heart failure, myocardial infarction, and cognitive impairment cases; about 32% of major bleeding events can be detected for the top 10% of high-risk members.

The ML-based formulations were nonlinear in nature and mostly dominated by interactive terms and fewer polynomial and main effects (see suppl Table S5). Both Medicaid group type and AF contributed significantly to the associations with adverse clinical outcomes.

**Figure 1:**
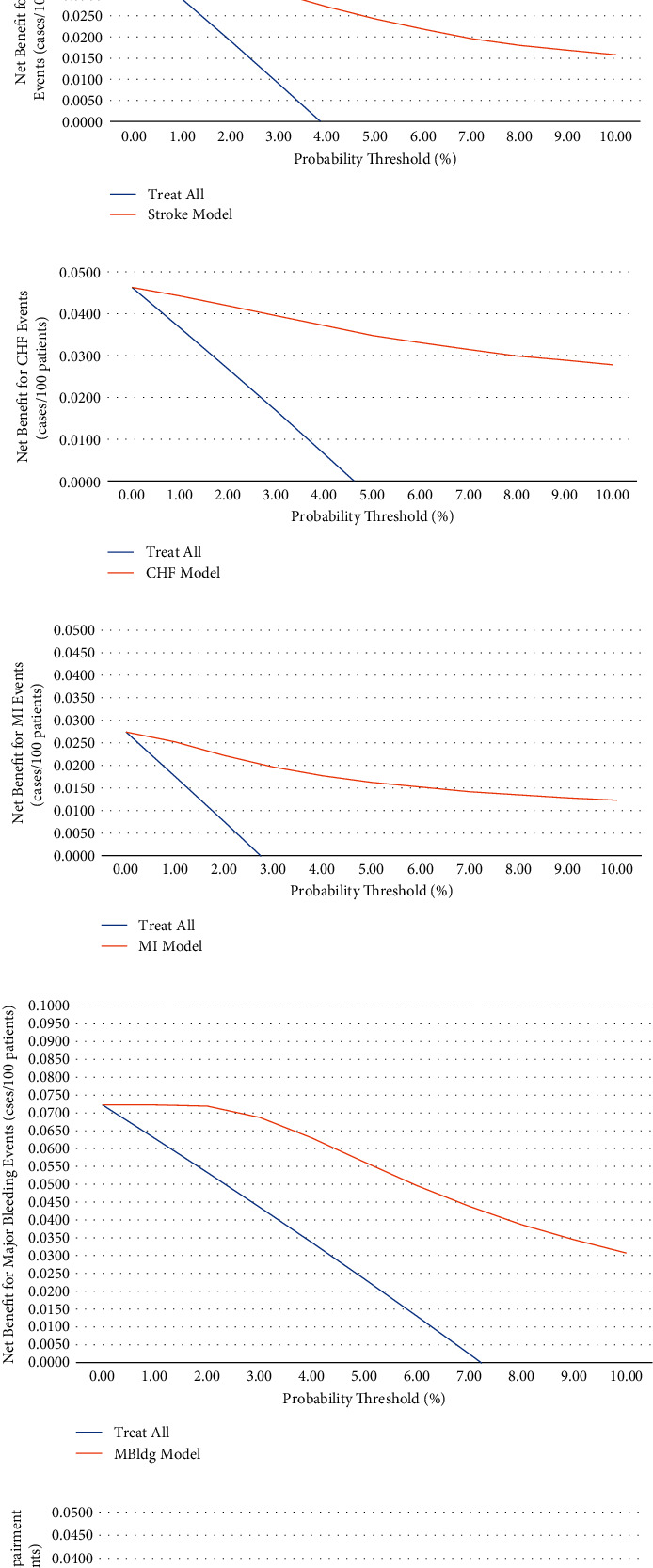
Clinical utility for ML logistic regression-based prediction models ((a)—AF; (b)—stroke; (c)—CHF; (d)—MI; (e)—major bleeding; (f)—cognitive impairment).

Figure 1 shows the decision curve analysis result for all ML models. The developed models produced better results in terms of net true positives than the “treat all” option even in the presence of low prevalence for the diagnosed conditions. Selecting a probability threshold of 2% for AF and cognitive impairment outcomes as the separator between low- and high-risk outcomes had corresponding sensitivity/specificity values of 71.2%/90.1% and 87.6%/86.3%, respectively, for the AF and cognitive impairment outcomes. A probability threshold of 3.5% would be adequate for stroke, CHF “congestive heart failure,” and MI “myocardial infarction” outcomes and having sensitivity/specificity values of 74.3%/72.5%, 83.1%/76.4%, and 71.9%/82.6%, respectively. Finally, a higher threshold of 6.5% was satisfactory for major bleeding (sensitivity/specificity values: 64.5%/65.3%), having the highest prevalence among the outcomes.

## 4. Discussion

In this study, our principal finding shows how combination(s) of socioeconomic status and disability contributes to AF incidence and complications, elevating risks to higher levels relative to the general population. Second, ML algorithms can be used to identify these AF patients at high risk of clinical events in these groups with low socioeconomic status and disability. Moreover, we identified the distinct Medicaid groups which are at the highest risk of AF incidence and complications.

It has been suggested that poor socioeconomic status is associated with an increased risk of AF incidence, but the literature is not definitive on this relationship. A recent systematic review found no consistent pattern for an association between socioeconomic status and the risk of AF. [15] One Chinese study [16] found that the prevalence of AF was highest in high-income regions (2.54%), followed by middle-income regions (2.33%), and lowest in low-income regions (1.98%). On the other hand, a European study [17] found that high-income groups tended to have the lowest levels of AF risk relative to low-income cohorts. A life-course disadvantaged socioeconomic status is an important predictor of the first hospitalization of AF. [4] Nonetheless, the US lacks a comprehensive study of different combinations of disadvantaged socioeconomic and disability cohorts such as those enrolled in the Medicaid population.

Of note, in this Medicaid population, the great majority of the population examined was under 65 years of age, and the overall crude incidence rate was distinctively high (0.49 cases/100 person-years (95% CI 0.40–0.58) and ranged from 0.20 to 1.5, which are relatively high compared to the published literature for this age group). For example, Wilke et al. [18] found incidence rates of 0.436 cases/100 person-years for men and 0.387 for women for the German population. Miyasaka et al. [19] reported an increase in age-/gender-adjusted incidence of AF per 100 person-years from 0.304 (95% CI 0.278–0.331) in 1980 to 0.368 (95% CI 0.342–0.395) in 2000 based on a general cohort in a Minnesota county in the US, so the higher incidence rates obtained in the present investigation are suggestive of the examined cohort being a sicker group than the general population. Furthermore, mental issues such as depression are also paramount as the prevalence was considerably high as well (19.2%).

The above argument is further supported by the incidence ratios of complications of higher AF risk patients. The incidence ratios of adverse clinical outcomes were as follows: 22.3% for stroke, 45.6% for heart failure, 21.7% for myocardial infarction, 24.2% for major bleeding, and 9.2% for cognitive impairment relative to the following ratios for the non-AF cohorts (3.7% for stroke, 4.2% for heart failure, 2.5% for myocardial infarction, and 1.3% for cognitive impairment).

The contribution of Medicaid group type toward the high AF incidence and adverse clinical outcomes was clearly demonstrated in strong terms both as an independent effect as well as interaction with comorbid profile (e.g., depression, vascular disease, and diabetes mellitus) and demographic variables (i.e., age groups and gender). The findings of this investigation clearly suggest that a poor socioeconomic status coupled with disability constraints may have negative consequences for AF incidence and associated AF-related complications. These were indeed demonstrated in the ML models developed for the detection of high-risk AF incidence and their associated complications, particularly in light of their high discriminant validity/performance effectiveness (based on cumulative lift) and high calibration/clinical utility. Further research would help advance the role of population health studies with respect to improved quality of care and cost of care savings.

### 4.1. Limitations

This study is observational in nature and may be limited by its inherent biases as well as the use of administrative databases. Yet, our findings support the findings of European studies on the role of poor socioeconomic status as a risk factor for AF incidence and its potential complications.

## 5. Conclusions

A combination of low socioeconomic status and disability constraints contributed significantly to AF incidence and complications, elevating the risk to higher levels relative to the general population. The use of ML algorithms revealed significant nonlinear associations which can be used to target high-risk AF patients for cardiovascular prevention programs.

## Figures and Tables

**Table 1:** Baseline characteristics for Medicaid groups and overall cohort. Values are shown in numbers (%), with exceptions as shown in the table.

Baseline characteristic	TANF	Family care	Two groups	LTSS—nondual	ABD—nondual	LTSS—duals	ABD—duals	Population
Age group (years)								
18–44	228848 (72.8)	13321 (56.7)	68843 (56.0)	14666 (49.9)	31991 (43.6)	2061 (6.9)	5857 (24.1)	365587 (59.2)
45–54	58651 (18.7)	6605 (28.1)	20298 (16.5)	8085 (27.5)	21180 (28.8)	2665 (9.0)	4787 (19.7)	122271 (19.8)
55–64	26566 (8.5)	3579 (15.2)	26580 (21.6)	6308 (21.5)	16866 (23.0)	4592 (15.4)	5498 (22.6)	89989 (14.6)
65–74	59 (0.0)	2 (0.0)	4248 (3.5)	250 (0.9)	2294 (3.1)	9516 (32.0)	5695 (23.5)	22064 (3.6)
75–90	17 (0.0)	0 (0.0)	2923 (2.4)	98 (0.3)	1104 (1.5)	10914 (36.7)	2446 (10.1)	17502 (2.8)
Age (years), mean (SD)	36.8 (11.8)	40.8 (12.3)	42.7 (15.9)	42.7 (13.0)	45.0 (14.0)	68.6 (13.9)	56.2 (15.3)	41.7 (15.2)

Gender								
Males	95961 (47.0)	11366 (48.4)	38252 (31.1)	13835 (47.0)	33992 (46.3)	9354 (31.4)	9860 (40.6)	212620 (34.4)
Females	218180 (53.0)	12141 (51.6)	84640 (68.9)	15572 (53.0)	39443 (53.7)	20394 (68.6)	14423 (59.4)	404793 (65.6)
Total	314141 (100.0)	23507 (100.0)	122892 (100.0)	29407 (100.0)	73435 (100.0)	29748 (100.0)	24283 (100.0)	617413 (100.0)

Comorbid history								
Congestive heart failure	3206 (1.0)	324 (1.4)	5260 (4.3)	2050 (7.0)	3772 (5.1)	2002 (6.7)	1391 (5.7)	18005 (2.9)
Hypertension	87325 (27.8)	9073 (38.6)	46765 (38.1)	15713 (53.4)	37236 (50.7)	13241 (44.5)	12235 (50.4)	221588 (35.9)
Diabetes mellitus	20671 (6.6)	2232 (9.5)	14853 (12.1)	5733 (19.5)	11379 (15.5)	4048 (13.6)	4297 (17.7)	63213 (10.2)
Stroke	4727 (1.5)	488 (2.1)	4470 (3.6)	1697 (5.8)	3173 (4.3)	1268 (4.3)	833 (3.4)	16656 (2.7)
Ischemic stroke	2224 (0.7)	283 (1.2)	3073 (2.5)	1326 (4.5)	2191 (3.0)	1030 (3.5)	528 (2.2)	10655 (1.7)
Transient ischemic attack	2806 (0.9)	283 (1.2)	1646 (1.3)	537 (1.8)	1160 (1.6)	264 (0.9)	322 (1.3)	6970 (1.1)
Thromboembolic events	349 (0.1)	48 (0.2)	486 (0.4)	147 (0.5)	363 (0.5)	37 (0.1)	97 (0.4)	1525 (0.2)
Vascular disease	8407 (2.7)	968 (4.1)	7736 (6.3)	2879 (9.8)	5980 (8.1)	1614 (5.4)	2066 (8.5)	29650 (4.8)
Myocardial infarction	3072 (1.0)	372 (1.6)	3031 (2.5)	876 (3.0)	2651 (3.6)	311 (1.0)	671 (2.8)	10984 (1.8)
Peripheral artery disease	5628 (1.8)	639 (2.7)	5321 (4.3)	2201 (7.5)	3771 (5.1)	1357 (4.6)	1499 (6.2)	20416 (3.3)
Valvular disease	8426 (2.7)	875 (3.7)	4858 (4.0)	1432 (4.9)	3932 (5.4)	424 (1.4)	1181 (4.9)	21128 (3.4)
Coronary artery disease	6731 (2.1)	866 (3.7)	7305 (5.9)	2262 (7.7)	5749 (7.8)	1730 (5.8)	2374 (9.8)	27017 (4.4)
Chronic sleep apnea	4765 (1.5)	508 (2.2)	2189 (1.8)	904 (3.1)	1959 (2.7)	154 (0.5)	507 (2.1)	10986 (1.8)
Chronic kidney disease	4014 (1.3)	491 (2.1)	5831 (4.7)	2291 (7.8)	4129 (5.6)	1944 (6.5)	2131 (8.8)	20831 (3.4)
Chronic pulmonary obstructive disease /bronchiectasis	27511 (8.8)	2206 (9.4)	16719 (13.6)	5983 (20.3)	15918 (21.7)	2824 (9.5)	4357 (17.9)	75518 (12.2)
Major bleeding	17127 (5.5)	1535 (6.5)	7656 (6.2)	2391 (8.1)	5637 (7.7)	771 (2.6)	1319 (5.4)	36436 (5.9)
Cognitive impairment	703 (0.2)	105 (0.4)	1594 (1.3)	638 (2.2)	928 (1.3)	1575 (5.3)	398 (1.6)	5941 (1.0)
Liver disease	27587 (8.8)	2386 (10.2)	11440 (9.3)	4460 (15.2)	9131 (12.4)	723 (2.4)	1961 (8.1)	57688 (9.3)
Anemia	43835 (14.0)	4015 (17.1)	19387 (15.8)	5808 (19.8)	11646 (15.9)	2437 (8.2)	3291 (13.6)	90419 (14.6)
Depression	57261 (18.2)	3012 (12.8)	23725 (19.3)	8273 (28.1)	18782 (25.6)	2416 (8.1)	5074 (20.9)	118543 (19.2)
Lipid disorders	74824 (23.8)	8651 (36.8)	33194 (27.0)	12376 (42.1)	27014 (36.8)	5235 (17.6)	9062 (37.3)	170356 (27.6)
Spondylosis and intervertebral discs	117952 (37.5)	8774 (37.3)	43662 (35.5)	11965 (40.7)	31565 (43.0)	3450 (11.6)	8509 (35.0)	225877 (36.6)
Osteoarthritis	27353 (8.7)	2936 (12.5)	17253 (14.0)	6028 (20.5)	13877 (18.9)	3601 (12.1)	4529 (18.7)	75577 (12.2)
Hyperthyroidism	4122 (1.3)	377 (1.6)	1443 (1.2)	409 (1.4)	915 (1.2)	76 (0.3)	202 (0.8)	7544 (1.2)
Metabolic syndrome	1818 (0.6)	166 (0.7)	568 (0.5)	263 (0.9)	534 (0.7)	35 (0.1)	111 (0.5)	3495 (0.6)
Asthma	35210 (11.2)	3099 (13.2)	15373 (12.5)	4909 (16.7)	12475 (17.0)	932 (3.1)	2636 (10.9)	74634 (12.1)

**Table 2:** Atrial fibrillation crude incidence rates (cases/100 person-years) and ratios for Medicaid groups and overall cohort.

Variable		^#^AF events	Follow-up time (person-years)	Incidence rate (cases/100 person-years)	Incidence ratio
Medicaid group	TANF	2137	1092473	0.20	0.68
	Family care	641	81089	0.79	2.73
	Two groups	2639	429690	0.61	2.15
	LTSS—nondual	650	105704	0.61	2.21
	ABD—nondual	1775	258443	0.69	2.42
	LTSS—duals	1477	102150	1.45	4.97
	ABD—duals	1281	85090	1.51	5.28

Total		10600	2154639	0.49	3.37
				490	

Note: AF—atrial fibrillation.

**Table 3:** Odds ratios “OR” (95% CI) for relationships between baseline characteristics of comorbid history/demographic variables/Medicaid group categories and atrial fibrillation status group as an outcome, with cost threshold (of ^$^2000 or more in the year prior to index date or the equivalent for non-AF cases) as a model feature.

Variable	Level	OR	95% CL	
Congestive heart failure	1 vs 0	2.38	2.24	2.53
Hypertension	1 vs 0	1.56	1.48	1.64
Diabetes mellitus	1 vs 0	1.18	1.12	1.24
Stroke	1 vs 0	1.30	1.21	1.40
Vascular disease	1 vs 0	1.21	1.14	1.29
Valvular disease	1 vs 0	1.62	1.52	1.73
Coronary artery disease	1 vs 0	1.36	1.28	1.44
Chronic sleep apnea	1 vs 0	1.16	1.04	1.29
Chronic kidney disease	1 vs 0	1.36	1.28	1.45
Chronic pulmonary obstructive disease/bronchiectasis	1 vs 0	1.53	1.46	1.61
Major bleeding	1 vs 0	1.20	1.12	1.28
Cognitive impairment	1 vs 0			
Liver disease	1 vs 0	1.17	1.10	1.24
Anemia	1 vs 0	1.29	1.23	1.36
Depression	1 vs 0	1.19	1.13	1.24
Lipid disorders	1 vs 0	0.93	0.89	0.97
Spondylosis and intervertebral discs	1 vs 0	1.13	1.08	1.18
Osteoarthritis	1 vs 0	1.09	1.04	1.15
Hyperthyroidism	1 vs 0	1.24	1.06	1.44
Metabolic syndrome	1 vs 0	0.58	0.43	0.77
Asthma	1 vs 0	0.70	0.65	0.75
Gender	1 vs 0	0.74	0.71	0.77

Age group	4 vs 0	7.01	6.41	7.66
	3 vs 0	4.17	3.82	4.56
	2 vs 0	2.28	2.14	2.44
	1 vs 0	1.72	1.61	1.83

Medicaid group	6 vs 0	1.62	1.48	1.78
	5 vs 0	2.43	2.24	2.65
	4 vs 0	1.28	1.16	1.40
	3 vs 0	1.54	1.44	1.65
	2 vs 0	1.55	1.45	1.65
	1 vs 0	3.22	2.93	3.52

Cost threshold (1 year prior)		1.62	1.54	1.71

Note: 1—The presence of comorbid condition; 0—the absence of comorbid condition; 75–90 (4) vs 18–44 years (0); 65–74 (3) vs 18–44 years (0); 55–64 (2) vs 18–44 years (0); 45–54 (1) vs 18–44 years (0); LTSS—duals (6) vs TANF (0); ABD—duals (5) vs TANF (0); LTSS—nondual (4) vs TANF (0); ABD—nondual (3) vs TANF (0); two groups (2) vs TANF (0); family care (1) vs TANF (0); model feature based on cost threshold of 1 year prior for ^$^2000 or more (1 or > ^$^2000; 0 otherwise); TANF group (1 or > $8750 that is applied for AF cohort only; 0 otherwise); 0—absence of comorbid condition, CL—confidence limits, and C index = 0.822. Data are based on the entire population.

**Table 4:** Odds ratios “OR” (95% CI) for main effect relationships between baseline characteristics of comorbid history and atrial fibrillation status group and five clinical outcomes, with cost threshold (of 2000 or more in the year prior to index date or the equivalent for non-AF cases) as a model feature.

Variable	Level	OR	Stroke	
			Lower limit	Upper limit
AF Status	1 vs 0	2.69	2.53	2.85
Congestive heart failure	1 vs 0	1.06	1.00	1.12
Hypertension	1 vs 0	1.56	1.50	1.61
Diabetes mellitus	1 vs 0	1.28	1.23	1.33
Stroke	1 vs 0	9.86	9.49	10.25
Vascular disease	1 vs 0	1.46	1.40	1.53
Valvular disease	1 vs 0	1.12	1.06	1.18
Coronary artery disease	1 vs 0	1.15	1.10	1.21
Chronic sleep apnea	**1 vs 0**	**0.97**	**0.89**	**1.05**
Chronic kidney disease	1 vs 0	1.13	1.07	1.19
Chronic pulmonary obstructive disease/bronchiectasis	1 vs 0	1.08	1.04	1.12
Major bleeding	1 vs 0	1.23	1.18	1.29
Cognitive impairment	1 vs 0	1.20	1.10	1.31
Liver disease	1 vs 0	1.10	1.06	1.14
Anemia	1 vs 0	1.14	1.10	1.19
Depression	1 vs 0	1.08	1.04	1.11
Lipid disorders	1 vs 0	1.18	1.15	1.22
Spondylosis and intervertebral discs	1 vs 0	1.17	1.13	1.20
Osteoarthritis	**1 vs 0**	**1.03**	**0.99**	**1.06**
Hyperthyroidism	**1 vs 0**	**1.04**	**0.93**	**1.16**
Metabolic syndrome	**1 vs 0**	**1.03**	**0.88**	**1.21**
Asthma	1 vs 0	1.10	1.06	1.15
Gender	**1 vs 0**	**0.98**	**0.95**	**1.01**
Age group	4 vs 0	2.16	2.00	2.34
	3 vs 0	2.25	2.10	2.42
	2 vs 0	2.09	2.00	2.18
	1 vs 0	1.89	1.81	1.96
Cost threshold (1 year prior)	1 vs 0	1.63	1.58	1.69
Medicaid group	6 vs 0	1.38	1.29	1.48
	**5 vs 0**	**1.05**	**0.98**	**1.13**
	4 vs 0	1.87	1.77	1.97
	3 vs 0	1.41	1.35	1.47
	2 vs 0	1.25	1.20	1.30
	1 vs 0	1.04	0.96	1.13

Variable	Level	OR	CHF	
			Lower limit	Upper limit

AF Status	1 vs 0	6.18	5.86	6.52
Congestive heart failure	1 vs 0	18.23	17.53	18.95
Hypertension	1 vs 0	1.91	1.85	1.98
Diabetes mellitus	1 vs 0	1.54	1.48	1.60
Stroke	1 vs 0	1.02	0.97	1.09
Vascular disease	1 vs 0	1.31	1.25	1.37
Valvular disease	1 vs 0	1.55	1.47	1.63
Coronary artery disease	1 vs 0	1.56	1.49	1.63
Chronic sleep apnea	1 vs 0	1.29	1.20	1.39
Chronic kidney disease	1 vs 0	1.72	1.64	1.80
Chronic pulmonary obstructive disease/bronchiectasis	1 vs 0	1.54	1.49	1.60
Major bleeding	1 vs 0	1.04	0.99	1.10
Cognitive impairment	1 vs 0	0.94	0.85	1.03
Liver disease	1 vs 0	1.03	0.99	1.08
Anemia	1 vs 0	1.16	1.12	1.20
Depression	1 vs 0	1.03	1.00	1.07
Lipid disorders	1 vs 0	0.89	0.86	0.92
Spondylosis and intervertebral discs	1 vs 0	0.98	0.95	1.01
Osteoarthritis	1 vs 0	1.04	1.00	1.08
Hyperthyroidism	1 vs 0	0.98	0.87	1.10
Metabolic syndrome	1 vs 0	1.07	0.91	1.26
Asthma	1 vs 0	1.17	1.13	1.22
Gender	1 vs 0	0.90	0.87	0.93
Age group	4 vs 0	2.63	2.44	2.83
	3 vs 0	2.08	1.94	2.22
	2 vs 0	1.80	1.72	1.88
	1 vs 0	1.69	1.62	1.76
Cost threshold (1 year prior)	1 vs 0	1.59	1.53	1.65
Medicaid group	6 vs 0	1.86	1.73	1.99
	5 vs 0	1.84	1.73	1.97
	4 vs 0	2.63	2.49	2.78
	3 vs 0	2.02	1.93	2.11
	2 vs 0	1.70	1.63	1.78
	1 vs 0	1.13	1.03	1.24

Variable	Level	OR	MI	
			Lower limit	Upper limit

AF Status	1 vs 0	3.71	3.49	3.94
Congestive heart failure	1 vs 0	1.57	1.49	1.65
Hypertension	1 vs 0	1.96	1.87	2.05
Diabetes mellitus	1 vs 0	1.22	1.17	1.27
Stroke	1 vs 0	1.04	0.98	1.11
Vascular disease	1 vs 0	3.36	3.22	3.51
Valvular disease	1 vs 0	1.11	1.05	1.18
Coronary artery disease	1 vs 0	3.72	3.56	3.89
Chronic sleep apnea	1 vs 0	0.94	0.86	1.02
Chronic kidney disease	1 vs 0	1.30	1.22	1.37
Chronic pulmonary obstructive disease/bronchiectasis	1 vs 0	1.32	1.27	1.37
Major bleeding	1 vs 0	1.08	1.02	1.14
Cognitive impairment	1 vs 0	0.91	0.81	1.02
Liver disease	1 vs 0	1.11	1.06	1.16
Anemia	1 vs 0	0.98	0.94	1.02
Depression	1 vs 0	1.14	1.10	1.19
Lipid disorders	1 vs 0	0.99	0.96	1.03
Spondylosis and intervertebral discs	1 vs 0	1.13	1.09	1.17
Osteoarthritis	1 vs 0	0.93	0.89	0.97
Hyperthyroidism	1 vs 0	0.93	0.81	1.07
Metabolic syndrome	1 vs 0	0.92	0.77	1.11
Asthma	1 vs 0	1.12	1.07	1.17
Gender	1 vs 0	0.72	0.70	0.75
Age group	4 vs 0	1.48	1.33	1.64
	3 vs 0	1.54	1.41	1.68
	2 vs 0	1.64	1.56	1.72
	1 vs 0	1.77	1.69	1.85
Cost threshold (1 year prior)	1 vs 0	1.33	1.27	1.38
Medicaid group	6 vs 0	0.63	0.57	0.70
	5 vs 0	1.09	1.00	1.18
	4 vs 0	1.26	1.18	1.35
	3 vs 0	1.55	1.48	1.63
	2 vs 0	1.18	1.12	1.24
	1 vs 0	1.20	1.10	1.31

Variable	Level	OR	MBldg	
			Lower limit	Upper limit

AF Status	1 vs 0	2.26	2.14	2.38
Congestive heart failure	1 vs 0	1.04	0.99	1.09
Hypertension	1 vs 0	1.17	1.14	1.19
Diabetes mellitus	1 vs 0	1.03	1.00	1.07
Stroke	1 vs 0	1.30	1.24	1.36
Vascular disease	1 vs 0	1.10	1.05	1.14
Valvular disease	1 vs 0	1.11	1.06	1.16
Coronary artery disease	1 vs 0	1.07	1.03	1.12
Chronic sleep apnea	1 vs 0	1.03	0.97	1.10
Chronic kidney disease	1 vs 0	1.23	1.18	1.29
Chronic pulmonary obstructive disease/bronchiectasis	1 vs 0	1.15	1.12	1.18
Major bleeding	1 vs 0	3.79	3.69	3.89
Cognitive impairment	1 vs 0	1.18	1.09	1.28
Liver disease	1 vs 0	1.44	1.40	1.48
Anemia	1 vs 0	1.28	1.24	1.31
Depression	1 vs 0	1.23	1.21	1.26
Lipid disorders	1 vs 0	1.06	1.04	1.09
Spondylosis and intervertebral discs	1 vs 0	1.31	1.28	1.33
Osteoarthritis	1 vs 0	1.08	1.06	1.12
Hyperthyroidism	1 vs 0	1.07	0.99	1.16
Metabolic syndrome	1 vs 0	1.10	0.98	1.23
Asthma	1 vs 0	1.17	1.14	1.20
Gender	1 vs 0	0.85	0.83	0.86
Age group	4 vs 0	1.16	1.08	1.26
	3 vs 0	1.08	1.01	1.15
	2 vs 0	1.17	1.14	1.21
	1 vs 0	1.20	1.17	1.23
Cost threshold (1 year prior)	1 vs 0	1.60	1.56	1.64
Medicaid group	6 vs 0	0.51	0.48	0.55
	5 vs 0	0.88	0.83	0.93
	4 vs 0	1.19	1.15	1.24
	3 vs 0	1.10	1.06	1.13
	2 vs 0	0.91	0.88	0.93
	1 vs 0	1.05	1.00	1.11

Variable	Level	OR	CogI	
			Lower limit	Upper limit

AF Status	1 vs 0	1.74	1.59	1.91
Congestive heart failure	1 vs 0	0.82	0.75	0.90
Hypertension	1 vs 0	1.03	0.98	1.09
Diabetes mellitus	1 vs 0	0.94	0.88	1.00
Stroke	1 vs 0	1.33	1.22	1.44
Vascular disease	1 vs 0	1.19	1.10	1.28
Valvular disease	1 vs 0	0.95	0.86	1.05
Coronary artery disease	1 vs 0	1.00	0.92	1.08
Chronic sleep apnea	1 vs 0	1.04	0.90	1.20
Chronic kidney disease	1 vs 0	0.99	0.91	1.08
Chronic pulmonary obstructive disease/bronchiectasis	1 vs 0	1.08	1.01	1.14
Major bleeding	1 vs 0	1.11	1.02	1.21
Cognitive impairment	1 vs 0	20.24	18.97	21.59
Liver disease	1 vs 0	1.21	1.12	1.29
Anemia	1 vs 0	1.22	1.15	1.30
Depression	1 vs 0	1.61	1.52	1.70
Lipid disorders	1 vs 0	1.13	1.07	1.19
Spondylosis and intervertebral discs	1 vs 0	1.05	1.00	1.11
Osteoarthritis	1 vs 0	0.96	0.91	1.02
Hyperthyroidism	1 vs 0	1.02	0.83	1.25
Metabolic syndrome	1 vs 0	0.82	0.59	1.14
Asthma	1 vs 0	0.92	0.85	0.99
Gender	1 vs 0	0.87	0.83	0.92
Age group	4 vs 0	12.47	11.27	13.79
	3 vs 0	6.56	5.92	7.26
	2 vs 0	2.95	2.73	3.19
	1 vs 0	1.98	1.84	2.14
Cost threshold (1 year prior)	1 vs 0	2.37	2.22	2.53
Medicaid group	6 vs 0	2.06	1.86	2.29
	5 vs 0	1.67	1.48	1.87
	4 vs 0	3.81	3.47	4.18
	3 vs 0	2.07	1.90	2.26
	2 vs 0	2.26	2.09	2.44
	1 vs 0	1.64	1.39	1.92

1—The presence of comorbid condition, AF status, and female gender; 0—the absence of comorbid condition. Data on the entire population. C index = 0.820 for stroke, 0.889 for CHF, 0.853 for MI, 0.718 for major bleeding, and 0.870 for cognitive impairment; CHF—congestive heart failure, MI—myocardial infarction, MBldg—major bleeding, CogI—cognitive impairment. 75–90 (4) vs 18–44 years (0), 65–74 (3) vs 18–44 years (0), 55–64 (2) vs 18-44 years (0), 45-54 (1) vs 18-44 years (0), LTSS—duals (6) vs TANF (0), ABD—duals (5) vs TANF (0), LTSS—nondual (4) vs TANF (0), ABD—nondual (3) vs TANF (0), two groups (2) vs TANF (0), and family care (1) vs TANF (0).

**Table 5:** Performance assessment for prediction models of high-risk atrial fibrillation and associated adverse clinical outcomes (stroke, heart failure, myocardial infarction, major bleeding, and cognitive impairment).

*Part a—assessment in terms of discriminant validity (i.e., c index or area under the curve)*													
*Outcome*	*C index*												
	*Training*				*Validation*								
	*Estimate*		*95% lower limit*	*95% upper limit*	*Estimate*	*95% lower limit*	*95% upper limit*						

AF outcome with condition presence and a minimum of $5000 of total annual care cost in the year prior to index date	*0.910*		0.891	0.929	*0.910*	0.883	0.937						
Stroke	*0.825*		0.809	0.841	*0.820*	0.797	0.843						
Heart failure	*0.891*		0.885	0.897	*0.888*	0.879	0.897						
Myocardial infarction	*0.857*		0.835	0.879	*0.860*	0.829	0.891						
Major bleeding	*0.716*		0.678	0.754	*0.714*	0.661	0.767						
Cognitive impairment	*0.870*		0.837	0.875	*0.874*	0.824	0.878						

*Part b—assessment in terms of the effectiveness of % targeted members (i.e., cumulative lift)*													
Member targeted (%)		Validation —Cumulative lift											
		AF outcome with condition presence and a minimum of $5000 of total annual care cost in the year prior to index date		Stroke		Heart failure		Myocardial infarction		Major bleeding		Cognitive impairment	
		Cumulative lift (multiplier)	Cumulative lift (proportion of % of member targeted)	Cumulative lift (multiplier)	Cumulative lift (proportion of % of member targeted)	Cumulative lift (multiplier)	Cumulative lift (proportion of % of member targeted)	Cumulative lift (multiplier)	Cumulative lift (proportion of % of member targeted)	Cumulative lift (multiplier)	Cumulative lift (proportion of % of member targeted	Cumulative lift (multiplier)	Cumulative lift (proportion of % of member targeted)
5		11.12	*55.62*	7.39	*36.95*	10.25	*51.26*	9.01	*45.04*	4.07	*20.37*	10.06	*50.30*
10		7.00	*70.02*	4.95	*49.46*	6.39	*63.94*	5.91	*59.10*	3.16	*31.61*	6.28	*62.78*
15		6.22	*93.35*	3.86	*57.87*	4.81	*72.14*	4.49	*67.38*	2.66	*39.84*	4.69	*70.29*
20		4.22	*84.38*	3.24	*64.70*	3.89	*77.80*	3.67	*73.46*	2.35	*46.92*	3.83	*76.62*
25		3.55	*88.80*	2.82	*70.45*	3.29	*82.25*	3.13	*78.18*	2.13	*53.15*	3.24	*81.08*
30		3.06	*91.71*	2.51	*75.15*	2.86	*85.65*	2.76	*82.74*	1.95	*58.41*	2.82	*84.57*

## Data Availability

Data are available as presented in the paper. According to US laws and corporate agreements, our own approvals to use the Anthem and Ingenio-Rx data sources for the current study do not allow us to distribute or make patient data directly available to other parties.

## Supplementary Materials

Supplementary Materials The supplementary material includes: (a) Healthcare codes for extracting atrial fibrillation from medical and pharmacy claims as well as comorbid history; (b) Details of machine learning-based models for atrial fibrillation outcomes and associated complications (i.e., stroke, congestive heart failure, myocardial infarction, major bleeding, and cognitive impairment, together with performance assessment analyses); and (c) Details of quantitative analyses.
