# Supplementary material for: Incidence and Complications of Atrial Fibrillation in a Low Socioeconomic and High Disability United States (US) Population: A Combined Statistical and Machine Learning Approach
Source: Int J Clin Pract. 2022 Aug 30;2022:8649050. doi: 10.1155/2022/8649050 (PMC9448617; doi:10.1155/2022/8649050)
Supplement: Supplementary Materials — The supplementary material includes: (a) Healthcare codes for extracting atrial fibrillation from medical and pharmacy claims as well as comorbid history; (b) Details of machine learning-based models for atrial fibrillation outcomes and associated complications (i.e., stroke, congestive heart failure, myocardial infarction, major bleeding, and cognitive impairment, together with performance assessment analyses); and (c) Details of quantitative analyses. [file 8649050.f1.docx]

**Table S1** NDC codes for anticoagulant and rhythm control medications as well as health care service codes for atrial fibrillation in medical claims

| **Medication / Healthcare Service Type** | **Medication / Healthcare Service Name** | **Code Name** | **Code Values** |
| --- | --- | --- | --- |
| Anticoagulant | Warfarin | NDC | '00182267101','00182267110','00182267189','00339653712','00615454729','00615454753','00615454763','00781035207', |
|  |  |  | '51079090820','59772035204','59772035207','59772035208','00056016901','00056016970','00056016975','00056016990', |
|  |  |  | '00093171201','00378880101','00378880110','00406205201','00406205210','00555083102','00555083105','00832121100', |
|  |  |  | '00832121101','00832121110','00832121189','15330010001','15330010010','21695067230','23490647801','23490647802', |
|  |  |  | '23490647803','31722032701','31722032710','35356054090','35356058230','35356058260','35356058290','42549049730', |
|  |  |  | '43063047130','43063065530','43353002830','43353002860','43353049330','43353049360','43353058430','43353058460', |
|  |  |  | '51138005430','51138017930','51138019530', |
|  |  |  | '51138048210','51138048230','51672402701','51672402703','51672402707','52959092430','54569444300','54569444301', |
|  |  |  | '54569622500','54569622501','54868212800','54868212801','54868212802','54868212803','54868434900','54868434901', |
|  |  |  | '54868434902','54868434903','54868434905','55045288001','55048085630','55289034030','55887026430','55887026460', |
|  |  |  | '55887026482','55887026490','57237011901','57237011999','58118402703','58118402706','58118402709','58864035715', |
|  |  |  | '58864077315','58864077330','60429078401','60429078410','60429078415','60429078430','60429078445','60429078477', |
|  |  |  | '60760003130','63629401701','63629401702','63629401703','63629401704','63629401705','65162076110','65162076111', |
|  |  |  | '66105051810','66267028530','66267062900','66336024920','66336024930','66336024960','68084014677','68115035930', |
|  |  |  | '68115035960','68115035990','68258910401','68382005201','68382005210','76282032701','76282032710','00056017030', |
|  |  |  | '00182267201','00182267210','00182267289','00339653812','00615150929','00615150953','00615150963','51079090920', |
|  |  |  | '54569015801','55289014397','59772036304','59772036307','59772036308','00056017001','00056017070','00056017075', |
|  |  |  | '00056017090','00093171301','00093171310','00378880201','00378880210','00406205301','00406205310','00555086902', |
|  |  |  | '00555086905','00781036307','00832121200','00832121201','00832121210','00832121289','15330010101','15330010110', |
|  |  |  | '21695067330','21695067360','23490648001','23490648002','23490648003','31722032801','31722032810','33358036000', |
|  |  |  | '35356057130','35356057160','35356057190','42549049630','43353002130','43353002135','43353002140','43353002145', |
|  |  |  | '43353002150','43353002153','43353002155','43353002161','51138005530','51138018030','51138018060','51138019630', |
|  |  |  | '51138019660','51138048330','51672402801','51672402803','51672402807','52959092530','53217021930','53217021990', |
|  |  |  | '54569015800','54569622400','54868082200','54868212900','54868212901','54868212902','54868212903','54868442200', |
|  |  |  | '54868442201','54868442202','54868442203','54868442204','54868442205','55045290208','55048086130','55887092690', |
|  |  |  | '57237012001','57237012099','58118402803','58864003014','58864003030','58864030114','58864087930','60429078501', |
|  |  |  | '60429078510','60429078515','60429078530','60429078535','60429078540','60429078545','60429078560','60429078577', |
|  |  |  | '60429078590','60760004030','62584098401','62584098411','62584098477','63629412201','63629412202','63629412203', |
|  |  |  | '63629412204','63629412205','63629412206','63739036001','63739036003','63739036015','65162076210','65162076211', |
|  |  |  | '66116046930','66267063600','66336025020','66336025030','66336025090','67544031815','67544031830','67544031835', |
|  |  |  | '67544031840','67544031845','67544031850','67544031853','67544031855','67544031860','67544031861','67544031870', |
|  |  |  | '67544040115','67544040130','67544040135','67544040140','67544040145','67544040150','67544040153','67544040155', |
|  |  |  | '67544040160','67544040161','67544040170','68115009330','68115039930','68258102601','68382005301','68382005310', |
|  |  |  | '76282032801','76282032810','00056017630','00182267301','00182267310','00182267389','00339653912','00615151029', |
|  |  |  | '51079091020','59772036404','59772036407','59772036408','62584098601','00056017601','00056017670','00056017675', |
|  |  |  | '00056017690','00093171401','00093171410','00378882501','00378882510','00406206401','00406206410','00555083202', |
|  |  |  | '00555083205','00781036407','00832121301','00832121310','00832121389','12280031230','12280031260','12280031290', |
|  |  |  | '15330010201','15330010210','16590034030','16590034060','16590034090','21695067430','23490648101','23490648102', |
|  |  |  | '23490648103','31722032901','31722032910','33261099730','33261099760','33261099790','35356039730','35356039760', |
|  |  |  | '35356039790','43353002930','43353002935','43353002940','43353002945','43353002953','43353002960','43353014230', |
|  |  |  | '43353014235','43353014240','43353014245','43353014253','43353014260','49999041130','51138005630','51138018130', |
|  |  |  | '51138019730','51138048410','51138048430','51138048445','51672402901','51672402903','51672402907','54569021201', |
|  |  |  | '54569021202','54569586800','54569586801','54868215400','54868215401','54868215402','54868215403','54868440000', |
|  |  |  | '54868440001','54868440002','54868440003','54868440004','55048085730','55887057730','55887057760','55887057790', |
|  |  |  | '57237012101','57237012199','58118402903','58118402906','58118402909','58864003530','60429078601','60429078610', |
|  |  |  | '60429078615','60429078630','60429078645','60429078677','60760003330','63187074530','63629317701','63629317702', |
|  |  |  | '63739036101','63739036103','63739036110','63739036115','65162076310','65162076311','66105017610','66267063000', |
|  |  |  | '66336025130','66336025160','67544019530','67544019540','67544019545','67544019553','67544019560','68084002701', |
|  |  |  | '68084002711','68084002777','68115009230','68258910101','68382006401','68382006410','76282032901','76282032910', |
|  |  |  | '00182267401','00182267489','00339654012','00615454829','00615454853','00615454863','00781036607','51079091120', |
|  |  |  | '59772036607','00056018801','00056018870','00056018875','00056018890','00093171501','00378880301','00378880310', |
|  |  |  | '00406205401','00406205410','00555092502','00832121400','00832121401','00832121410','00832121489','15330026601', |
|  |  |  | '21695067530','31722033001','31722033010','33261099830','33261099860','33261099890','35356090630','35356090690', |
|  |  |  | '43353003030','43353003060','43353008930','43353008960','43353049230','43353049260','43683011830','51138005730', |
|  |  |  | '51138018230','51138019830','51138048510','51138048530','51672403001','51672403003','51672403007','53217023530', |
|  |  |  | '54569639400','54868406300','54868406301','54868487100','54868487101','54868487102','54868487103','54868542500', |
|  |  |  | '57237012201','57237012299','58118403003','58118403006','58118403009','60429078701','60429078710','60429078715', |
|  |  |  | '60429078730','60429078745','60429078777','60760004130','63739036201','63739036203','63739036210','63739036215', |
|  |  |  | '65162076410','65162076411','66267063100','68084014777','68382005401','68382005410','71335058001','71335058002', |
|  |  |  | '76282033001','76282033010','00182267501','00182267589','00339654112','00615454929','00615454953','00615454963', |
|  |  |  | '00781036907','51079091220','59772036907','59772036908','00056016801','00056016870','00056016875','00056016890', |
|  |  |  | '00093171601','00378880401','00378880410','00406205501','00406205510','00555087402','00555087405','00832121500', |
|  |  |  | '00832121501','00832121510','00832121589','15330026701','21695093930','23490648201','23490648202','23490648203', |
|  |  |  | '31722033101','31722033110','33261099000','33261099030','33261099060','33261099090','43063021830','43353003330', |
|  |  |  | '43353004930','43353004960','49999092310','49999092330','49999092360','49999092390','51138005830','51138018330', |
|  |  |  | '51138019930','51138048610','51138048630','51672403101','51672403103','51672403107','54569586900','54868082500', |
|  |  |  | '54868339900','54868339901','54868440200','54868440201','54868440202','54868440203','55048085830','55887046430', |
|  |  |  | '55887046460','55887046490','57237012301','57237012399','58016008300','58016008330','58016008360','58016008390', |
|  |  |  | '58118403103','58118403106','58118403109','60429078801','60429078810','60429078815','60429078830','60429078845', |
|  |  |  | '60429078877','60760004330','60760070630','63629474801','63739036301','63739036303','63739036310','63739036315', |
|  |  |  | '65162076510','65162076511','66267063200','67544019430','67544019460','68084014877','68258606703','68382005501', |
|  |  |  | '68382005510','71335045201','71335045202','71335045203','71335045204','76282033101','76282033110','00056017230', |
|  |  |  | '00182267601','00182267610','00182267689','00339654212','00615151229','00781037707','51079091320','54569015901', |
|  |  |  | '55175538003','55289028601','55289028697','59772037704','59772037707','59772037708','60346038125','62584094477', |
|  |  |  | '00056017201','00056017270','00056017275','00056017290','00093172110','00378880501','00378880510','00406205601', |
|  |  |  | '00406205610','00555083302','00555083305','00832121600','00832121601','00832121610','00832121689','15330026801', |
|  |  |  | '15330026810','16590034130','16590034160','16590034190','21695067730','23490648301','23490648302','23490648303', |
|  |  |  | '31722033201','31722033210','33261035707','33261035714','33261035720','33261035721','33261035728','33261035730', |
|  |  |  | '33261035760','33261035790','33358036130','43063017614','43063017630','43353002311','43353002330','43353002335', |
|  |  |  | '43353002338','43353002340','43353002344','43353002345','43353002346','43353002350','43353002353','43353002365', |
|  |  |  | '43353002370','43353005011','43353005015','43353005020','43353005021','43353005025','43353005028','43353005030', |
|  |  |  | '43353005035','43353005038','43353005040','43353005044','43353005045','43353005046','43353005047','43353005050', |
|  |  |  | '43353005053','43353005055','43353005059','43353005060','43353005061','43353005065','43353005068','43353005070', |
|  |  |  | '43353005078','43683011730','49999009330','49999057600','49999057610','49999057620','49999057630','49999057660', |
|  |  |  | '49999057690','50090002800','51138005930','51138018430','51138020030','51138048710','51138048720','51138048730', |
|  |  |  | '51138048775','51672403203','51672403207','52959092630','54569015900','54569493400','54569493401','54569493402', |
|  |  |  | '54868125900','54868125901','54868125902','54868125903','54868125904','54868125905','54868125906','54868125907', |
|  |  |  | '54868428600','54868428601','54868428602','54868428603','54868428604','54868428605','54868520700','54868520701', |
|  |  |  | '55045288108','55048085930','55048088030','55289028614','55289028630','55289028650','55289077314','55289077330', |
|  |  |  | '55289077360','55289077390','55887057810','55887057830','55887057860','55887057886','55887057890','57237012401', |
|  |  |  | '57237012499','58118403203','58517036030','58864022314','58864022330','58864069814','58864069830','60429078901', |
|  |  |  | '60429078910','60429078915','60429078920','60429078925','60429078930','60429078935','60429078940','60429078945', |
|  |  |  | '60429078950','60429078960','60429078975','60429078977','60429078990','60760003430','61919034130','62584099401', |
|  |  |  | '62584099411','62584099477','63187067410','63187067460','63187067490','63629254801','63629254802','63739036401', |
|  |  |  | '63739036403','63739036410','63739036415','65162076610','65162076611','65243027403','66105011010','66116047030', |
|  |  |  | '66267026830','66267063300','66336025214','66336025230','66336025260','66336025290','67544005215','67544005220', |
|  |  |  | '67544005225','67544005228','67544005230','67544005235','67544005238','67544005240','67544005245','67544005250', |
|  |  |  | '67544005253','67544005255','67544005257','67544005260','67544005261','67544005265','67544005268','67544005270', |
|  |  |  | '67544005278','68115009430','68115052730','68115052760','68115052790','68115065900','68258102701','68258910201', |
|  |  |  | '68382005601','68382005610','68382005616','71335024301','71335024302','71335024303','71335024304','71335024305', |
|  |  |  | '71335024306','71610017330','71610017335','71610017340','71610017345','76282033201','76282033210','00182267701', |
|  |  |  | '00182267789','00339654312','00615455029','00781038107','51079091420','59772038107','00056018901','00056018970', |
|  |  |  | '00056018975','00056018990','00093171801','00378880601','00378880610','00406205701','00555092602','00832121700', |
|  |  |  | '00832121701','00832121710','00832121789','15330010601','33261099930','43353058730','51138006030','51138018530', |
|  |  |  | '51138020130','51138048810','51138048830','51672403301','51672403303','54569631200','54868121600','54868487300', |
|  |  |  | '54868487301','54868487302','54868487303','54868487304','54868525500','54868525501','55700000530','55700000560', |
|  |  |  | '55700000590','57237012501','57237012599','58118403303','58118403306','58118403309','60429079001','60429079010', |
|  |  |  | '60429079015','60429079030','60429079045','60429079077','65162076710','65162076711','66105052110','66267063400', |
|  |  |  | '67544007030','68382005701','76282033301','76282033310','00182267801','00182267889','00339654412','00555083405', |
|  |  |  | '00615455129','00781038607','51079091520','59772038607','00056017301','00056017370','00056017375','00093171901', |
|  |  |  | '00093172301','00378887501','00378887510','00406205801','00555083402','00832121800','00832121801','00832121850', |
|  |  |  | '00832121889','15330010701','21695094030','23490648401','23490648402','23490648403','31722033401','43353005330', |
|  |  |  | '43353049430','43353057930','49999082900','51138006130','51138018630','51138020230','51138048930','51672403401', |
|  |  |  | '51672403403','53217001830','53217001860','53217001890','54569631300','54569631301','54868225200','54868225201', |
|  |  |  | '54868495000','54868495001','54868495002','57237012601','57237012699','58118403403','58118403406','58118403409', |
|  |  |  | '60429079101','60429079115','60429079130','60429079145','60429079177','63629441701','63629441702','63629441703', |
|  |  |  | '65162076810','65162076811','66267063500','66336082530','68258909701','68382005801','76282033401','00182267901', |
|  |  |  | '00182267989','00339654512','00555083504','00615455729','00781038707','51079091620','59772038707','00056017401', |
|  |  |  | '00056017470','00056017475','00093172001','00378881001','00378881010','00406205901','00555083502','00832121900', |
|  |  |  | '00832121901','00832121950','00832121989','15330010801','21695080130','31722033501','43353005430','43353049130', |
|  |  |  | '43353057809','43353057830','51138006230','51138018730','51138020330','51138049010','51138049030','51672403501', |
|  |  |  | '51672403503','53217000100','53217000130','53217000160','53217000190','54569642700','54868245400','54868245401', |
|  |  |  | '54868245402','54868525800','55887056730','55887056760','55887056790','57237012701','57237012799','58016069700', |
|  |  |  | '58016069730','58016069760','58016069790','58118403503','58118403506','58118403509','60429079201','60429079230', |
|  |  |  | '60429079245','60429079277','63187075010','63187075030','65162076910','65162076911','66105052310','66267062800', |
|  |  |  | '68258606803','68258906401','68382005901','76282033501','00590032435','00590032496','49452813601','49452813602', |
|  |  |  | '51927247100','38779047405','38779047410','38779047425','38779047504','38779047505' |
|  | Pradaxa | NDC | '00597010754','00597010760','00597014954','00597014960','00597035509','00597035556','00597035561','00597010854', |
|  |  |  | '00597010860','00597013554','00597013560','00597036055','00597036082','21695089960','54569627600' |
|  | Eliquis | NDC | '00003089321','00003089331','50090143600','54569651300','00003089421','00003089431','00003089470','50090143700', |
|  |  |  | '54569651400' |
|  | Xarelto | NDC | '50458058010','50458058030','50458058090','50458057810','50458057830','50458057890','42254037601','50458057910', |
|  |  |  | '50458057930','50458057989','50458057990' |
| Rhythm control | Disopyramide | NDC | '00182174301','00332312709','00332312713','00339568112','00603340821','00615039213','00677102001','00781211001', |
|  |  |  | '00814261414','00904248240','00904248260','00904248261','00025275252','00093312705','00364073901','00364073990', |
|  |  |  | '00025275231','00093312701','00591556001','23629001610','42291023401','51138054930','51407013101','51862009301', |
|  |  |  | '51862009501','54868218100','00182174401','00332312909','00332312913','00339568312','00603340921','00615039313', |
|  |  |  | '00677102101','00781211501','00814261514','00904248340','00904248360','00904248361','00093312905','00364074001', |
|  |  |  | '00364074090','00025276231','00025276252','00093312901','00591556101','42291023501','51138055030','51407013201', |
|  |  |  | '51862009401','54868381600','00025273231','00025273234','00025273251','00339568412','54868132500','00025274231', |
|  |  |  | '00025274234','00025274251','54868069200','58177000204' |
|  | Procainamide | NDC | '00182070589','00223145601','00223145602','00349217901','00364021901','00364021902','00536436701','00603540421', |
|  |  |  | '00615034213','00677045001','00814633514','00904234570','00904234580','51079010120','00172234580','00003075850', |
|  |  |  | '00172234560','54868336700','54868346800','68258906901','00182092501','00182092589','00364034301','00364034390', |
|  |  |  | '00536437701','00677066701','00814633714','00839156306','00904234660','51079012520','00172234660','00172234680', |
|  |  |  | '55289016397','60346099620','00003075650','00003075750','00172234780','00182052189','00223145701','00223145702', |
|  |  |  | '00349218001','00364034401','00364034402','00364034490','00536436801','00603540621','00615034313','00677047001', |
|  |  |  | '00814634014','00839505006','00904234770','00904234780','51079010220','00172234760','54868346900','00003043150', |
|  |  |  | '00003043450','00003043850','00349839401','00814634314','00839702706','00904773660','00364071501','00182170801', |
|  |  |  | '00182170805','00349839501','00349839505','00349897801','00349897805','00364071601','00364071605','00536557601', |
|  |  |  | '00536557605','00603541121','00603541128','00615257713','00814634414','00814634428','00839702806','00904236840', |
|  |  |  | '00904236860','38245018810','38245018850','52555048001','52555048005','54569049300','00677098701','00003077550', |
|  |  |  | '00093918801','00093918805','54868069401','00182170901','00349839601','00536435801','00536435805','00603541221', |
|  |  |  | '00814634514','00839702906','38245011410','38245011450','52555045601','54569049400','00677098801','54868217000', |
|  |  |  | '00093911401','00093911405','38245011710','00093911701','00071056220','00071056240','61570006960','61570006901', |
|  |  |  | '61570006970','68258914701','00071056420','00071056440','61570007160','61570007101','61570007170' |
|  | Quinidine Gluconate | NDC | '00054874925','00182138201','00182138202','00182138205','00349704001','00349704005','00349704010','00349704025', |
|  |  |  | '00536443401','00536443402','00536443405','00615158301','00615158305','00615158313','00615158339','00615158353', |
|  |  |  | '00615158363','00781180401','00781180405','00781180413','00781180425','00814652222','00839647306','00839647309', |
|  |  |  | '00839647312','46703005325','49727040902','53258015701','53258015713','58016056200','58016056260','62584089501', |
|  |  |  | '00339532712','00364060401','00364060404','00364060405','00603559821','00603559828','00904220240','00904220260', |
|  |  |  | '00904220270','50419010110','50419010111','50419010125','50419010150','54569049600','54569049601','55175013201', |
|  |  |  | '57480038801','58864045430','00591553801','00591553805','00591553825','00677067501','00677067503','00677067505', |
|  |  |  | '23490936503','51079002701','51079002720','53489014101','53489014103','53489014105','54738090101','54738090102', |
|  |  |  | '54868069801','60429016760','60429016790','62451010150' |
|  | Quinidine Polygalacturonate | NDC | '00034547080','00034547090' |
|  | Quinidine Sulfate | NDC | '00005355823','00005355834','00054473631','00054873325','00182014401','00349214901','00349214910','00364022990', |
|  |  |  | '00536443201','00536443210','00603559432','00615051501','00615051510','00615051513','00615051532','00781190001', |
|  |  |  | '00781190010','00814652014','00814652030','00839506306','00839506316','00904220180','46703001910','51079003120', |
|  |  |  | '53258015813','54569049700','00364022901','00364022902','00904220160','55289022297','60346062725','60346062760', |
|  |  |  | '60346062790','00185434601','00185434610','00591543801','00591543810','00677012201','00677012210','00904220161', |
|  |  |  | '53489046101','53489046110','54868004703','00054473525','00054473531','00054873525','00349827801','00364058290', |
|  |  |  | '00536442901','00603559521','00781190201','00781190213','00814652114','00839660506','00904220380','54569049800', |
|  |  |  | '00364058201','00677120910','00904220360','53489046010','00185104701','00185104710','00591545401','00677120901', |
|  |  |  | '53489046001','54868089801','00031664964','00182199701','00536569501','38245017510','38245017525','54569065500', |
|  |  |  | '00031664963','00031664967','54868274001','54868274003','59911589501','59911589502','00093917501','00093917552' |
|  | Mexiletine | NDC | '00054861625','00364264101','00536574801','00597006661','00615132629','00615132643','00615132665','00615132688', |
|  |  |  | '57480083601','57480083606','62939231201','00054261625','00247123630','00597006601','00615132653','00615132663', |
|  |  |  | '00781213001','52544049101','54569473200','55953073940','55953073941','59930168501','00093873901','00591049101', |
|  |  |  | '42291062401','49999091690','54868377600','54868377601','55045363401','55045363402','58016074000','58016074002', |
|  |  |  | '58016074004','58016074020','58016074030','58016074060','58016074090','58016074099','00054861725','00364264201', |
|  |  |  | '00536574901','00597006761','57480083701','57480083706','62939232201','00054261725','00247123730','00597006701', |
|  |  |  | '00781213101','52544049201','54569478900','55953074040','55953074041','59930168601','00093874001','00591049201', |
|  |  |  | '42291062501','55045365101','00364264301','00597006861','55953074140','62939233201','00054261825','00597006801', |
|  |  |  | '00781213201','52544049301','59930168701','00093874101','00591049301','42291062601','49999091790','55045363501', |
|  |  |  | '55045363502','00186070728','61113070728','61113070768','00186070768','00186070928','61113070928','61113070968', |
|  |  |  | '00186070968' |
|  | Flecainide | NDC | '00054001020','00054001021','00054001025','00089030510','00378850501','00440754260','00555085902','00781506201', |
|  |  |  | '29336030510','42291029301','49884069401','50268032011','50268032015','51079098720','51407001801','54569613100', |
|  |  |  | '54868506500','54868506501','54868507400','54868507401','54868507402','54868507403','55045381901','57237006301', |
|  |  |  | '60687022525','60687022595','63304079401','65162064110','66105015202','66105015203','66105015206','66105015209', |
|  |  |  | '66105015210','99207018050','00054001120','00054001121','00054001125','00089030710','00378851001','00440754360', |
|  |  |  | '00555086002','00781506301','12280018230','29336030710','42291029401','49884069501','50268032111','50268032115', |
|  |  |  | '51079098820','51407001901','51407001960','54569613200','54868440700','54868540500','54868540501','57237006401', |
|  |  |  | '58016096202','58016096203','58016096204','58016096230','58016096240','58016096256','58016096260','58016096290', |
|  |  |  | '63304079501','65162064210','68084054011','68084054021','68084054025','68084054095','68258608403','99207018110', |
|  |  |  | '00781506401','00054001220','00054001221','00054001225','00089031410','00378851501','00440754460','00555086102', |
|  |  |  | '29336031410','42291029501','49884069601','50268032211','50268032215','51407002001','54868558600','54868558601', |
|  |  |  | '57237006501','62559038201','63304079601','65162064310','99207018215' |
|  | Propafenone | NDC | '00044502202','00044502210','00074162812','00677181505','52544058201','53489055105','00074162814','00173079220', |
|  |  |  | '00591058201','00603544821','00603544825','00677181501','00677181503','50111070801','51079099601','51079099620', |
|  |  |  | '53489055101','53489055103','54868477000','54868477001','54868477002','54868477003','54868477004','54868477005', |
|  |  |  | '58016030300','58016030330','58016030360','58016030390','58177033104','58177033111','58177033112','62559023001', |
|  |  |  | '63629386901','63739050910','65726026525','65726026590','68084036101','68084036111','00044502402','00044502410', |
|  |  |  | '00074173212','00677181605','52544058301','53489055205','00074173214','00173079420','00591058301','00603544921', |
|  |  |  | '00603544925','00677181601','00677181603','50111070901','53489055201','53489055203','54569613300','54868595000', |
|  |  |  | '54868595001','58177033204','62559023101','63739051010','65726026625','65726026690','00044502302','00044502310', |
|  |  |  | '00074183112','00677181703','00677181705','53489055303','53489055305','00074183114','00603545021','00677181701', |
|  |  |  | '50111071001','53489055301','58177033304','62559023201','65726026725','65726026790','00173078601','00173082318', |
|  |  |  | '00378125805','00378125891','00591228560','42291054560','49884009902','49884009905','49884009909','49884011302', |
|  |  |  | '49884011305','49884011309','49884020902','49884020905','49884020909','60429043660','60429097660','60687018532', |
|  |  |  | '60687018533','65726026115','65726026125','65726026190','66993011460','68084085832','68084085833','68462040860', |
|  |  |  | '00173078801','00173082418','00378125905','00378125991','00591228660','21695081460','42291054660','49884021002', |
|  |  |  | '49884021005','49884021009','60429043760','65726026215','65726026225','65726026290','66993011560','68084091732', |
|  |  |  | '68084091733','68462040960','00173078901','00173082618','00378126105','00378126191','00591228760','42291054760', |
|  |  |  | '49884021102','49884021105','49884021109','60429043860','65726026315','65726026325','65726026390','66993011660', |
|  |  |  | '68462041060' |
|  | Dofelitide | NDC | '00069580043','00069580060','00069580061','42291041160','47335006186','51862012560','59762003702','69452013117', |
|  |  |  | '00069581043','00069581060','00069581061','42291041260','47335006286','51862002560','59762003802','69452013217', |
|  |  |  | '00069582043','00069582060','00069582061','42291041360','42794004610','47335006386','51862000560','59762003902', |
|  |  |  | '69452013317' |
|  | Dronedarone | NDC | '00024414210','00024414218','00024414260','21695092060','54868308600' |
|  | Amiodarone | NDC | '00245014401','00245014430','00245014489','16714084301','42291011890','51862015430','51862024030','17236007560', |
|  |  |  | '38245013368','00008418806','00093913393','38245013325','38245013355','54569514000','55953021440','55953021441', |
|  |  |  | '55953021470','00008418804','00093913306','00185014405','00185014409','00185014460','00245014701','00245014715', |
|  |  |  | '00245014760','00245014789','00245014790','00555091704','00555091709','00781120305','00781120360','00781120392', |
|  |  |  | '00904655661','13107005605','13107005660','16714084401','16714084402','21695079630','21695079660','21695079690', |
|  |  |  | '35356000110','35356000130','35356000190','47463001360','49884045802','49884045804','49884045805','51079090601', |
|  |  |  | '51079090617','51079090619','51079090620','51138049130','51672402504','51862024105','51862024160','51862024190', |
|  |  |  | '54569514001','54868461800','54868461801','54868461802','54868461803','55048001360','55887079801','58016030400', |
|  |  |  | '58016030430','58016030460','58016030490','60429024705','60429024760','63739038710','65862073205','65862073260', |
|  |  |  | '67544017630','67544057030','68084037101','68084037111','68382022705','68382022714','00245014001','00245014030', |
|  |  |  | '00245014510','00245014501','00245014530','00245014589','42291013930','42494030903','51672405700','51672405706', |
|  |  |  | '51862015630','51862024230','54569612900' |
| Medical Condition | Atrial Fibrillation/Flutter | ICD10 | I480',' I481',’I4811’,’I4819’,' I482',’I4820’,’I4821’,'I483',' I484',’I489’, 'I4891', 'I4892' |
| Outpatient | Emergency Room | Revenue Code | '0450','0451','0452','0456','0459' |
|  |  | Inpatient Code | NO' |
| Inpatient | Hospital Admission | Type of Facility Code | 1' |
| Oupatient | Office Visit | CPT - New Patients | '99201','99202','99203','99204','99205' |
|  |  | CPT - Existing Patients | '99211','99212','99213','99214','99215' |
|  | Physician Observation | CPT | '99218','99219','99220' |
|  | Catheter Ablation (Procedure) | CPT | '93650','93653','93654','93655','93656','93657','93613' |
|  | Cardioversion (Procedure) | CPT | '92960','92959' |

**Table S2** Exceptions for use of anticoagulants and rhythm control

| **Source of Exception** | **Healthcare Service Type/Description** | **Code** | **Value** |
| --- | --- | --- | --- |
| Anticoagulants | Medical Condition - Pulmonary Embolus | ICD10 | 'I2601','I2602','I2609','I2690','I2692','I2699' |
|  | Medical Condition - Deep Vein Thrombosis | ICD10 | 'I80201','I80202','I80203','I80209','I80211','I80212','I80213','I80219','I80221','I80222', |
|  |  |  | 'I80223','I80229','I80231','I80232','I80233','I80239','I80291','I80292','I80293','I80299' |
|  | Procedures - Aortic / Mitral / Pulmonary Valve Replacement | CPT | 33361','33362','33363','33364','33365','33366','33367','33368','33369','33418','33419', |
|  |  |  | '33477','33999' |
| Rhythm Control | Procedures - Implantable Automatic Defibrillators | CPT | 33215','33216','33217','33218','33220','33223','33224','33225','33230','33231','33240', |
|  |  |  | 33241','33243','33244','33249','33262','33263','33264','33270','33271','33272','33273' |

**Table S3** ICD 10 codes for comorbid conditions

| **Comorbidity Condition** | **ICD10 Code Value** |
| --- | --- |
| Congestive Heart Failure Non-hypertensive | 'I0981','I501','I5020','I5021','I5022','I5023','I5030','I5031','I5032','I5033','I5040','I5041', |
|  | 'I5042','I5043','I50810','I50811','I50812','I50813','I50814','I5082','I5083','I5084','I5089','I509' |
| Essential Hypertension | 'I10' |
| Diabetes Mellitus 1 & 2 | 'E1010','E1011','E1021','E1022','E1029','E10311','E10319','E10321','E103211','E103212','E103213','E103219', |
|  | 'E10329','E103291','E103292','E103293','E103299','E10331','E103311','E103312','E103313','E103319','E10339','E103391', |
|  | 'E103392','E103393','E103399','E10341','E103411','E103412','E103413','E103419','E10349','E103491','E103492','E103493', |
|  | 'E103499','E10351','E103511','E103512','E103513','E103519','E103521','E103522','E103523','E103529','E103531','E103532', |
|  | 'E103533','E103539','E103541','E103542','E103543','E103549','E103551','E103552','E103553','E103559', |
|  | 'E10359','E103591','E103592','E103593','E103599','E1036','E1037X1','E1037X2','E1037X3','E1037X9', |
|  | 'E1039','E1040','E1041','E1042','E1043','E1044','E1049','E1051','E1052','E1059','E10610','E10618', |
|  | 'E10620','E10621','E10622','E10628','E10630','E10638','E10641','E10649','E1065','E1069','E108','E1100', |
|  | 'E1101','E1110','E1111','E1121','E1122','E1129','E11311','E11319','E11321','E113211','E113212','E113213','E113219','E11329', |
|  | 'E113291','E113292','E113293','E113299','E11331','E113311','E113312','E113313','E113319','E11339','E113391','E113392', |
|  | 'E113393','E113399','E11341','E113411','E113412','E113413','E113419','E11349','E113491','E113492','E113493','E113499', |
|  | 'E11351','E113511','E113512','E113513','E113519','E113521','E113522','E113523','E113529','E113531','E113532','E113533', |
|  | 'E113539','E113541','E113542','E113543','E113549','E113551','E113552','E113553','E113559','E11359','E113591','E113592', |
|  | 'E113593','E113599','E1136','E1137X1','E1137X2','E1137X3','E1137X9','E1139','E1140','E1141','E1142','E1143','E1144','E1149', |
|  | 'E1151','E1152','E1159','E11610','E11618','E11620','E11621','E11622','E11628','E11630','E11638','E11641','E11649','E1165','E1169','E118' |
| Ischemic Stroke | 'I6300','I63011','I63012','I63013','I63019','I6302','I63031','I63032','I63033','I63039','I6309','I6310','I63111','I63112','I63113','I63119', |
|  | 'I6312','I63131','I63132','I63133','I63139','I6319','I6320','I63211','I63212','I63213','I63219','I6322','I63231','I63232','I63233','I63239', |
|  | 'I6329','I6330','I63311','I63312','I63313','I63319','I63321','I63322','I63323','I63329','I63331','I63332','I63333','I63339','I63341','I63342', |
|  | 'I63343','I63349','I6339','I6340','I63411','I63412','I63413','I63419','I63421','I63422','I63423','I63429','I63431','I63432','I63433','I63439', |
|  | 'I63441','I63442','I63443','I63449','I6349','I6350','I63511','I63512','I63513','I63519','I63521','I63522','I63523','I63529','I63531','I63532', |
|  | 'I63533','I63539','I63541','I63542','I63543','I63549','I6359','I636','I638','I6381','I6389','I639' |
| Transient Ischemic Attack | 'G450','G451','G452','G453','G454','G458','G459' |
| Thrombo-Embolic Event | 'I7401','I7409','I7410','I7411','I7419','I742','I743','I744','I745','I748','I749', |
|  | 'I75011','I75012','I75013','I75019','I75021','I75022','I75023','I75029','I7581','I7589' |
| Acute Myocardial Infarction | I2101','I2102','I2109','I2111','I2119','I2121','I2129','I213','I214','I219','I21A1','I21A9','I220','I221','I222','I228','I229','I252' |
| Peripheral artery disease | I700','I701','I70201','I70202','I70203','I70208','I70209','I70211','I70212','I70213','I70218','I70219', |
|  | 'I70221','I70222','I70223','I70228','I70229','I70231','I70232','I70233','I70234','I70235','I70238','I70239', |
|  | 'I70241','I70242','I70243','I70244','I70245','I70248','I70249','I7025','I70291','I70292','I70293','I70298', |
|  | 'I70299','I708','I7090','I7091','I7092','I739','K550','K55011','K55012','K55019','K55021','K55022','K55029','K55031', |
|  | 'K55032','K55039','K55041','K55042','K55049','K55051','K55052','K55059','K55061','K55062','K55069','K551','K558','K559' |
| Valvular Disease | 'I050','I051','I052','I058','I059','I060','I061','I062','I068','I069','I070','I071','I072','I078','I079','I080', |
|  | 'I081','I082','I083','I088','I089','I091','I0989','I340','I341','I342','I348','I349','I350','I351','I352','I358', |
|  | 'I359','I360','I361','I362','I368','I369','I370','I371','I372','I378','I379' |
| Coronary Artery Disease | 'I2510','I25110','I25111','I25118','I25119','I25750','I25751','I25758','I25759','I25811' |
| Obstructive Sleep Apnea | 'G4730' |
| Chronic Kidney Disease | 'N181','N182','N183','N184','N185','N186','N189' |
| Chronic obstructive pulmonary disease/bronchiectasis | I2101','I2102','I2109','I2111','I2119','I2121','I2129','I213','I214','I219', 'I21A1','I21A9','I220','I221','I222','I228','I229','I252' |
| Major bleeding | 'H31301','H31302','H31303','H31309','H31311','H31312','H31313','H31319','H31411','H31412','H31413','H31419', |
|  | H3560','H3561','H3562','H3563','H35731','H35732','H35733','H35739','H4310','H4311''H4312','H4313','H47021','H47022', |
|  | H47023','H47029','I6000','I6001','I6002','I6010','I6011','I6012','I602','I6020','I6021','I6022','I6030','I6031','I6032','I604', |
|  | 'I6050','I6051','I6052','I606','I607','I608','I609','I610','I611','I612','I613','I614','I615','I616','I618','I619','I6200','I6201','I6202','I6203','I621','I629', |
|  | 'I6900','I6901','I69010','I69011','I69012','I69013','I69014','I69015','I69018','I69019','I69020','I69021','I69022','I69023', |
|  | 'I69028','I69031','I69032','I69033','I69034','I69039','I69041','I69042','I69043','I69044','I69049','I69051','I69052','I69053', |
|  | 'I69054','I69059','I69061','I69062','I69063','I69064','I69065','I69069','I69090','I69091','I69092','I69093','I69098','I6910', |
|  | 'I6911','I69110','I69111','I69112','I69113','I69114','I69115','I69118','I69119','I69120','I69121','I69122','I69123','I69128', |
|  | 'I69131','I69132','I69133','I69134','I69139','I69141','I69142','I69143','I69144','I69149','I69151','I69152','I69153','I69154', |
|  | 'I69159','I69161','I69162','I69163','I69164','I69165','I69169','I69190','I69191','I69192','I69193','I69198','I6920','I6921','I69210', |
|  | 'I69211','I69212','I69213','I69214','I69215','I69218','I69219','I69220','I69221','I69222','I69223','I69228','I69231','I69232', |
|  | 'I69233','I69234','I69239','I69241','I69242','I69243','I69244','I69249','I69251','I69252','I69253','I69254','I69259','I69261', |
|  | 'I69262','I69263','I69264','I69265','I69269','I69290','I69291','I69292','I69293','I69298', |
|  | D68311','D68312','D68318','D6832','D698','D699','H1130','H1131','H1132','H1133','H3120','H2100','H2101','H2102','H2103','H9220','H9221',' |
|  | H9222','H9223','I312','I850','I8501','I8511','K226','K250','K251','K252','K254','K255','K256','K260','K261','K262','K264','K265', |
|  | 'K266','K270','K271','K272','K274','K275','K276','K280','K281','K282','K284','K285','K286','K290','K2901', |
|  | 'K2921','K2931','K2941','K2951','K2961','K2971','K2981','K2991','K920','K921','K922','I9820','I983','K2210', |
|  | 'K2212','K2214','K2216','K6380','K3180','K5520','K625','K922' |
| Cognitive impairment | 'G3181','G3182','G3184','G3185','G3189','G319','G3183','G309','G3109' |
|  | 'K7041','K710','K7110','K7111','K717','K718','K719','K7200','K7201','K7210','K7211','K7290','K7291','K740', |
|  | 'K741','K742','K743','K744','K745','K7460','K7469','K750','K751','K7589','K759','K760','K761','K762','K763', |
|  | 'K764','K765','K766','K767','K7681','K7689','K769','K77','R160','R162','R17','R740','R748','R749','Z944' |
| Liver disease | 'D500','D501','D508','D509','D510','D511','D512','D513','D518','D519','D520','D521','D528','D529','D530','D531', |
|  | 'D532','D538','D539','D550','D551','D552','D553','D558','D559','D560','D561','D562','D563','D564','D565','D568', |
|  | 'D569','D580','D581','D582','D588','D589','D590','D591','D592','D593','D594','D595','D596','D598','D599','D600', |
|  | 'D601','D608','D609','D6101','D6109','D611','D612','D613','D61810','D61811','D61818','D6182','D6189','D619', |
|  | 'D630','D631','D638','D640','D641','D642','D643','D644','D6489','D649','Z8631','D62','D5700','D5701','D5702', |
|  | 'D571','D5720','D57211','D57212','D57219','D573','D5740','D57411','D57412','D57419','D5780','D57811','D57812', |
|  | 'D57819' |
| Depression | 'E780','E7800','E7801','E781','E782','E783','E784','E7841','E7849','E785' |
| Lipid disorders | 'E780','E7800','E7801','E781','E782','E783','E784','E7841','E7849','E785' |
| Spondylosis and intervertebral disc | 'M4320','M4321','M4322','M4323','M4324','M4325','M4326','M4327','M4328','M436','M4600', |
|  | 'M4601','M4602','M4603','M4604','M4605','M4606','M4607','M4608','M4609','M461','M4630','M4631', |
|  | 'M4632','M4633','M4634','M4635','M4636','M4637','M4638','M4639','M4640','M4641','M4642','M4643', |
|  | 'M4644','M4645','M4646','M4647','M4648','M4649','M4650','M4651','M4652','M4653','M4654','M4655', |
|  | 'M4656','M4657','M4658','M4659','M4680','M4681','M4682','M4683','M4684','M4685','M4686','M4687', |
|  | 'M4688','M4689','M4690','M4691','M4692','M4693','M4694','M4695','M4696','M4697','M4698','M4699', |
|  | 'M47011','M47012','M47013','M47014','M47015','M47016','M47019','M47022','M47029','M4710','M4711','M4712', |
|  | 'M4713','M4714','M4715','M4716','M4717','M4718','M4720','M4721','M4722','M4723','M4724','M4725','M4726','M4727', |
|  | 'M4728','M47811','M47812','M47813','M47814','M47815','M47816','M47817','M47818','M47819','M47891','M47892', |
|  | 'M47893','M47894','M47895','M47896','M47897','M47898','M47899','M479','M4800','M4801','M4802','M4803','M4804','M4805', |
|  | 'M4806','M48061','M48062','M4807','M4808','M4810','M4811','M4812','M4813','M4814','M4815','M4816','M4817','M4818', |
|  | 'M4819','M4820','M4821','M4822','M4823','M4824','M4825','M4826','M4827','M4830','M4831','M4832','M4833','M4834','M4835','M4836', |
|  | 'M4837','M4838','M489','M4980','M4981','M4982','M4983','M4984','M4985','M4986','M4987','M4988','M4989','M5000', |
|  | 'M5001','M5002','M50020','M50021','M50022','M50023','M5003','M5010','M5011','M5012','M50120','M50121','M50122','M50123', |
|  | 'M5013','M5020','M5021','M5022','M50220','M50221','M50222','M50223','M5023','M5030','M5031','M5032','M50320','M50321', |
|  | 'M50322','M50323','M5033','M5080','M5081','M5082','M50820','M50821','M50822','M50823','M5083','M5090','M5091','M5092','M50920', |
|  | 'M50921','M50922','M50923','M5093','M5104','M5105','M5106','M5107','M5114','M5115','M5116','M5117','M5124','M5125', |
|  | 'M5126','M5127','M5134','M5135','M5136','M5137','M5144','M5145','M5146','M5147','M5184','M5185','M5186','M5187', |
|  | 'M519','M530','M531','M532X1','M532X2','M532X3','M532X4','M532X5','M532X6','M532X7','M532X8','M532X9','M533','M5380', |
|  | 'M5381','M5382','M5383','M5384','M5385','M5386','M5387','M5388','M539','M5400','M5401','M5402','M5403', |
|  | 'M5404','M5405','M5406','M5407','M5408','M5409','M5410','M5411','M5412','M5413','M5414','M5415','M5416','M5417', |
|  | 'M5418','M542','M5430','M5431','M5432','M5440','M5441','M5442','M545','M546','M5481','M5489','M549','M62830', |
|  | 'M961','M9920','M9921','M9922','M9923','M9924','M9925','M9926','M9927','M9928','M9929','M9930','M9931','M9932', |
|  | 'M9933','M9934','M9935','M9936','M9937','M9938','M9939','M9940','M9941','M9942','M9943','M9944','M9945','M9946', |
|  | 'M9947','M9948','M9949','M9950','M9951','M9952','M9953','M9954','M9955','M9956','M9957','M9958','M9959','M9960', |
|  | 'M9961','M9962','M9963','M9964','M9965','M9966','M9967','M9968','M9969','M9970','M9971','M9972','M9973','M9974', |
|  | 'M9975','M9976','M9977','M9978','M9979' |
| Osteoarthritis | 'M151','M152','M153','M154','M158','M159','M160','M1610','M1611','M1612','M162','M1630','M1631', |
|  | 'M1632','M164','M1650','M1651','M1652','M166','M167','M169','M170','M1710','M1711','M1712','M172', |
|  | 'M1730','M1731','M1732','M174','M175','M179','M180','M1810','M1811','M1812','M182','M1830','M1831', |
|  | 'M1832','M184','M1850','M1851','M1852','M189','M19011','M19012','M19019','M19021','M19022','M19029', |
|  | 'M19031','M19032','M19039','M19041','M19042','M19049','M19071','M19072','M19079','M19111','M19112', |
|  | 'M19119','M19121','M19122','M19129','M19131','M19132','M19139','M19141','M19142','M19149','M19171', |
|  | 'M19172','M19179','M19211','M19212','M19219','M19221','M19222','M19229','M19231','M19232','M19239', |
|  | 'M19241','M19242','M19249','M19271','M19272','M19279','M1990','M1991','M1992','M1993' |
| Hyperthyroidism | 'E0500','E0501','E0510','E0511','E0520','E0521','E0530','E0531','E0540','E0541','E0580','E0581','E0590','E0591' |
| Metabolic syndrome | 'E8881' |
| Asthma | J4520','J4521','J4522','J4530','J4531','J4532','J4540','J4541','J4542','J4550','J4551','J4552','J45901','J45902','J45909','J45990', |
|  | 'J45991','J45998' |
| Atrial fibrillation | 'I480','I481','I4811','I4819','I482','I4820','I4821','I483','I484','I489','I4891','I4892' |

**Table S4** Terms of logistic regression-based ML model with atrial fibrillation status as outcome and inclusion of model feature defined as cost threshold of 1 year prior of $2000

Standard Wald

Parameter Levels DF Estimate Error Chi-Square Pr > ChiSq

Intercept 1 35.8407 3.0225 140.61 <.0001

MMI_2 1 0.2933 0.0467 39.51 <.0001

age 1 0.0386 0.00345 125.28 <.0001

stdy_prd 1 -1.2193 0.0263 2146.65 <.0001

ANEMIA*CHF 1 1 1 -0.0654 0.0197 11.06 0.0009

ANEMIA*COGI 1 1 1 0.0830 0.0249 11.08 0.0009

ANEMIA*cost_threshold 1 1 1 0.0582 0.0208 7.81 0.0052

ANEMIA*mdcd_grp1 1 6 1 0.1300 0.0512 6.44 0.0111

ANEMIA*mdcd_grp1 1 5 1 0.00538 0.0464 0.01 0.9077

ANEMIA*mdcd_grp1 1 4 1 0.00724 0.0522 0.02 0.8897

ANEMIA*mdcd_grp1 1 3 1 0.0789 0.0368 4.60 0.0319

ANEMIA*mdcd_grp1 1 2 1 0.0606 0.0317 3.65 0.0561

ANEMIA*mdcd_grp1 1 1 1 -0.2558 0.0648 15.60 <.0001

ASTHMA*COPD 1 1 1 -0.0440 0.0210 4.39 0.0361

ASTHMA*MS 1 1 1 0.1880 0.0375 25.08 <.0001

ASTHMA*VALVD 1 1 1 -0.0558 0.0277 4.07 0.0437

ASTHMA*cost_threshold 1 1 1 -0.0589 0.0266 4.90 0.0269

ASTHMA*mdcd_grp1 1 6 1 0.0806 0.0857 0.89 0.3468

ASTHMA*mdcd_grp1 1 5 1 -0.00450 0.0643 0.00 0.9442

ASTHMA*mdcd_grp1 1 4 1 0.1450 0.0608 5.69 0.0171

ASTHMA*mdcd_grp1 1 3 1 0.0888 0.0438 4.10 0.0429

ASTHMA*mdcd_grp1 1 2 1 0.1373 0.0408 11.34 0.0008

ASTHMA*mdcd_grp1 1 1 1 -0.4497 0.0935 23.13 <.0001

CAD*LIVD 1 1 1 0.0378 0.0189 3.99 0.0457

CAD*VD 1 1 1 -0.0582 0.0201 8.40 0.0037

CHF*c2hest 1 6 1 -4.2994 10.1947 0.18 0.6732

CHF*c2hest 1 5 1 -8.1823 . . . .

CHF*c2hest 1 4 1 0.2533 0.2909 0.76 0.3839

CHF*c2hest 1 3 1 -0.00244 0.0732 0.00 0.9734

CHF*c2hest 1 2 1 0.0904 0.0887 1.04 0.3078

CHF*c2hest 1 1 1 3.7981 2.9226 1.69 0.1937

CKD*STROKE 1 1 1 -0.1050 0.0216 23.62 <.0001

COGI*DM 1 1 1 0.0682 0.0330 4.27 0.0387

COPD*MS 1 1 1 0.1556 0.0340 20.90 <.0001

COPD*cost_threshold 1 1 1 0.0745 0.0216 11.93 0.0006

DEP*mdcd_grp1 1 6 1 0.1776 0.0509 12.16 0.0005

DEP*mdcd_grp1 1 5 1 -0.1282 0.0503 6.50 0.0108

DEP*mdcd_grp1 1 4 1 -0.0736 0.0507 2.11 0.1465

DEP*mdcd_grp1 1 3 1 -0.0973 0.0353 7.58 0.0059

DEP*mdcd_grp1 1 2 1 -0.0309 0.0306 1.02 0.3126

DEP*mdcd_grp1 1 1 1 0.1344 0.0577 5.43 0.0198

HTHD*STROKE 1 1 1 0.1424 0.0375 14.41 0.0001

HYP*chads2 1 4 1 0.1544 0.1273 1.47 0.2253

HYP*chads2 1 3 1 0.0854 0.0645 1.75 0.1857

HYP*chads2 1 2 1 -0.0402 0.0535 0.56 0.4526

HYP*chads2 1 1 1 -0.2247 0.0593 14.34 0.0002

LIPDIS*MS 1 1 1 0.0675 0.0239 7.96 0.0048

LIPDIS*VD 1 1 1 0.0531 0.0198 7.20 0.0073

LIPDIS*age_grp 1 4 1 0.0280 0.0427 0.43 0.5127

LIPDIS*age_grp 1 3 1 -0.0107 0.0402 0.07 0.7896

LIPDIS*age_grp 1 2 1 -0.0974 0.0266 13.42 0.0002

LIPDIS*age_grp 1 1 1 0.0545 0.0297 3.37 0.0666

LIPDIS*mdcd_grp1 1 6 1 0.2495 0.0509 24.02 <.0001

LIPDIS*mdcd_grp1 1 5 1 -0.0194 0.0434 0.20 0.6542

LIPDIS*mdcd_grp1 1 4 1 -0.00353 0.0520 0.00 0.9459

LIPDIS*mdcd_grp1 1 3 1 0.0323 0.0350 0.85 0.3562

LIPDIS*mdcd_grp1 1 2 1 0.0320 0.0302 1.12 0.2902

LIPDIS*mdcd_grp1 1 1 1 -0.2130 0.0549 15.03 0.0001

MS*cost_threshold 1 1 1 -0.3151 0.0359 76.93 <.0001

OSTEO*mdcd_grp1 1 6 1 0.1076 0.0467 5.30 0.0213

OSTEO*mdcd_grp1 1 5 1 0.0586 0.0436 1.80 0.1791

OSTEO*mdcd_grp1 1 4 1 -0.1821 0.0529 11.86 0.0006

OSTEO*mdcd_grp1 1 3 1 -0.00643 0.0349 0.03 0.8540

OSTEO*mdcd_grp1 1 2 1 -0.0240 0.0299 0.64 0.4226

OSTEO*mdcd_grp1 1 1 1 0.0380 0.0580 0.43 0.5122

STROKE*VALVD 1 1 1 -0.0802 0.0282 8.11 0.0044

VALVD*c2hest 1 6 1 -0.3544 0.2214 2.56 0.1094

VALVD*c2hest 1 5 1 0.0876 0.0754 1.35 0.2458

VALVD*c2hest 1 4 1 0.1234 0.0628 3.86 0.0494

VALVD*c2hest 1 3 1 -0.0679 0.0623 1.19 0.2759

VALVD*c2hest 1 2 1 0.00401 0.0545 0.01 0.9414

VALVD*c2hest 1 1 1 0.1480 0.0515 8.27 0.0040

VALVD*gndr 1 1 1 0.0545 0.0224 5.92 0.0150

VALVD*mdcd_grp1 1 6 1 0.2011 0.0798 6.35 0.0117

VALVD*mdcd_grp1 1 5 1 -0.0466 0.0609 0.59 0.4435

VALVD*mdcd_grp1 1 4 1 0.1640 0.0656 6.24 0.0125

VALVD*mdcd_grp1 1 3 1 -0.0284 0.0478 0.35 0.5527

VALVD*mdcd_grp1 1 2 1 -0.0156 0.0426 0.13 0.7139

VALVD*mdcd_grp1 1 1 1 -0.2907 0.0972 8.95 0.0028

VD*age_grp 1 4 1 0.1185 0.0468 6.41 0.0114

VD*age_grp 1 3 1 -0.0673 0.0482 1.95 0.1626

VD*age_grp 1 2 1 -0.0203 0.0292 0.48 0.4880

VD*age_grp 1 1 1 0.0416 0.0303 1.88 0.1702

age_grp*cost_threshold 4 1 1 -0.0758 0.0515 2.17 0.1408

age_grp*cost_threshold 3 1 1 -0.0345 0.0473 0.53 0.4659

age_grp*cost_threshold 2 1 1 -0.1007 0.0294 11.77 0.0006

age_grp*cost_threshold 1 1 1 0.0468 0.0324 2.09 0.1484

age_grp*mdcd_grp1 4 6 1 0.0964 0.1293 0.56 0.4559

age_grp*mdcd_grp1 4 5 1 0.3010 0.1259 5.72 0.0168

age_grp*mdcd_grp1 4 4 1 0.3543 0.3670 0.93 0.3343

age_grp*mdcd_grp1 4 3 1 -0.0383 0.1755 0.05 0.8272

age_grp*mdcd_grp1 4 2 1 0.5463 0.1151 22.54 <.0001

age_grp*mdcd_grp1 4 1 1 2.0421 8.4427 0.06 0.8089

age_grp*mdcd_grp1 3 6 1 -0.1002 0.1057 0.90 0.3433

age_grp*mdcd_grp1 3 5 1 -0.0744 0.0841 0.78 0.3764

age_grp*mdcd_grp1 3 4 1 0.2568 0.2822 0.83 0.3628

age_grp*mdcd_grp1 3 3 1 -0.3857 0.1420 7.38 0.0066

age_grp*mdcd_grp1 3 2 1 0.2326 0.0807 8.29 0.0040

age_grp*mdcd_grp1 3 1 1 -0.5215 2.2447 0.05 0.8163

age_grp*mdcd_grp1 2 6 1 0.0684 0.1073 0.41 0.5239

age_grp*mdcd_grp1 2 5 1 0.0690 0.0807 0.73 0.3927

age_grp*mdcd_grp1 2 4 1 -0.1376 0.1328 1.07 0.3004

age_grp*mdcd_grp1 2 3 1 0.1732 0.0710 5.95 0.0147

age_grp*mdcd_grp1 2 2 1 -0.2523 0.0522 23.38 <.0001

age_grp*mdcd_grp1 2 1 1 -0.5768 2.1243 0.07 0.7860

age_grp*mdcd_grp1 1 6 1 -0.0704 0.1364 0.27 0.6060

age_grp*mdcd_grp1 1 5 1 0.0383 0.0982 0.15 0.6963

age_grp*mdcd_grp1 1 4 1 -0.1354 0.1344 1.02 0.3136

age_grp*mdcd_grp1 1 3 1 0.1126 0.0764 2.17 0.1405

age_grp*mdcd_grp1 1 2 1 -0.1832 0.0666 7.56 0.0060

age_grp*mdcd_grp1 1 1 1 -0.6776 2.1251 0.10 0.7498

c2hest*cha2ds2_vasc 6 6 1 5.3042 . . . .

c2hest*cha2ds2_vasc 6 5 1 -5.3500 24.4859 0.05 0.8270

c2hest*cha2ds2_vasc 6 4 1 2.5309 7.3233 0.12 0.7296

c2hest*cha2ds2_vasc 6 3 1 0.0816 7.3428 0.00 0.9911

c2hest*cha2ds2_vasc 6 2 1 0.1017 7.3612 0.00 0.9890

c2hest*cha2ds2_vasc 6 1 1 5.8157 4.5203 1.66 0.1982

c2hest*cha2ds2_vasc 5 6 1 9.5919 4.3849 4.79 0.0287

c2hest*cha2ds2_vasc 5 5 1 4.6418 3.6787 1.59 0.2070

c2hest*cha2ds2_vasc 5 4 1 5.1804 2.9182 3.15 0.0759

c2hest*cha2ds2_vasc 5 3 1 5.3572 2.9189 3.37 0.0665

c2hest*cha2ds2_vasc 5 2 1 5.4237 2.9266 3.43 0.0639

c2hest*cha2ds2_vasc 5 1 0 0 . . . .

c2hest*cha2ds2_vasc 4 6 1 -0.9076 4.3393 0.04 0.8343

c2hest*cha2ds2_vasc 4 5 1 -4.2028 3.6837 1.30 0.2539

c2hest*cha2ds2_vasc 4 4 1 -3.4229 2.9270 1.37 0.2422

c2hest*cha2ds2_vasc 4 3 1 -3.3456 2.9269 1.31 0.2530

c2hest*cha2ds2_vasc 4 2 1 -3.2691 2.9310 1.24 0.2647

c2hest*cha2ds2_vasc 4 1 1 -3.3278 2.9963 1.23 0.2667

c2hest*cha2ds2_vasc 3 6 1 -2.7560 4.4744 0.38 0.5379

c2hest*cha2ds2_vasc 3 5 1 -3.7832 3.6791 1.06 0.3038

c2hest*cha2ds2_vasc 3 4 1 -3.2773 2.9194 1.26 0.2616

c2hest*cha2ds2_vasc 3 3 1 -3.3557 2.9188 1.32 0.2503

c2hest*cha2ds2_vasc 3 2 1 -3.1991 2.9206 1.20 0.2734

c2hest*cha2ds2_vasc 3 1 1 -3.3402 2.9254 1.30 0.2535

c2hest*cha2ds2_vasc 2 6 1 22.8713 17.3377 1.74 0.1871

c2hest*cha2ds2_vasc 2 5 1 -5.6549 3.7651 2.26 0.1331

c2hest*cha2ds2_vasc 2 4 1 -3.4741 2.9225 1.41 0.2345

c2hest*cha2ds2_vasc 2 3 1 -3.5301 2.9204 1.46 0.2268

c2hest*cha2ds2_vasc 2 2 1 -3.5016 2.9214 1.44 0.2307

c2hest*cha2ds2_vasc 2 1 1 -3.2286 2.9240 1.22 0.2695

c2hest*cha2ds2_vasc 1 6 1 -0.1206 2.8298 0.00 0.9660

c2hest*cha2ds2_vasc 1 5 1 -0.0438 2.7805 0.00 0.9874

c2hest*cha2ds2_vasc 1 4 1 0.1366 0.2264 0.36 0.5462

c2hest*cha2ds2_vasc 1 3 1 0.0804 0.1440 0.31 0.5766

c2hest*cha2ds2_vasc 1 2 1 0.0478 0.0876 0.30 0.5854

c2hest*cha2ds2_vasc 1 1 0 0 . . . .

c2hest*gndr 6 1 1 0.3516 0.2203 2.55 0.1105

c2hest*gndr 5 1 1 -0.0926 0.0955 0.94 0.3324

c2hest*gndr 4 1 1 0.0633 0.0731 0.75 0.3867

c2hest*gndr 3 1 1 -0.0783 0.0612 1.63 0.2010

c2hest*gndr 2 1 1 0.0479 0.0544 0.77 0.3787

c2hest*gndr 1 1 1 -0.1173 0.0452 6.73 0.0095

cha2ds2_vasc*mdcd_grp1 6 6 1 -1.7989 2.8329 0.40 0.5254

cha2ds2_vasc*mdcd_grp1 6 5 1 -2.8444 2.8054 1.03 0.3106

cha2ds2_vasc*mdcd_grp1 6 4 1 -2.0929 2.8815 0.53 0.4676

cha2ds2_vasc*mdcd_grp1 6 3 1 -1.2637 2.7900 0.21 0.6506

cha2ds2_vasc*mdcd_grp1 6 2 1 -2.5937 2.7705 0.88 0.3492

cha2ds2_vasc*mdcd_grp1 6 1 1 3.2143 20.7807 0.02 0.8771

cha2ds2_vasc*mdcd_grp1 5 6 1 0.6509 2.7478 0.06 0.8127

cha2ds2_vasc*mdcd_grp1 5 5 1 1.0263 2.7502 0.14 0.7090

cha2ds2_vasc*mdcd_grp1 5 4 1 1.0220 2.7566 0.14 0.7108

cha2ds2_vasc*mdcd_grp1 5 3 1 1.0557 2.7472 0.15 0.7008

cha2ds2_vasc*mdcd_grp1 5 2 1 1.0080 2.7410 0.14 0.7131

cha2ds2_vasc*mdcd_grp1 5 1 1 -4.6896 16.5284 0.08 0.7766

cha2ds2_vasc*mdcd_grp1 4 6 1 0.1992 0.1914 1.08 0.2980

cha2ds2_vasc*mdcd_grp1 4 5 1 0.3480 0.1875 3.44 0.0634

cha2ds2_vasc*mdcd_grp1 4 4 1 0.1018 0.2204 0.21 0.6441

cha2ds2_vasc*mdcd_grp1 4 3 1 0.2306 0.1635 1.99 0.1585

cha2ds2_vasc*mdcd_grp1 4 2 1 0.4185 0.1443 8.41 0.0037

cha2ds2_vasc*mdcd_grp1 4 1 1 0.0259 2.1937 0.00 0.9906

cha2ds2_vasc*mdcd_grp1 3 6 1 0.1744 0.1464 1.42 0.2336

cha2ds2_vasc*mdcd_grp1 3 5 1 0.3512 0.1238 8.05 0.0046

cha2ds2_vasc*mdcd_grp1 3 4 1 0.2088 0.1696 1.52 0.2183

cha2ds2_vasc*mdcd_grp1 3 3 1 -0.00087 0.1080 0.00 0.9936

cha2ds2_vasc*mdcd_grp1 3 2 1 0.3083 0.0904 11.64 0.0006

cha2ds2_vasc*mdcd_grp1 3 1 1 0.4900 2.1326 0.05 0.8183

cha2ds2_vasc*mdcd_grp1 2 6 1 -0.0125 0.1406 0.01 0.9289

cha2ds2_vasc*mdcd_grp1 2 5 1 0.4097 0.1131 13.13 0.0003

cha2ds2_vasc*mdcd_grp1 2 4 1 0.2344 0.1654 2.01 0.1564

cha2ds2_vasc*mdcd_grp1 2 3 1 -0.0435 0.0986 0.19 0.6594

cha2ds2_vasc*mdcd_grp1 2 2 1 0.2597 0.0821 10.01 0.0016

cha2ds2_vasc*mdcd_grp1 2 1 1 0.4593 2.1294 0.05 0.8292

cha2ds2_vasc*mdcd_grp1 1 6 1 0.2688 0.1487 3.27 0.0706

cha2ds2_vasc*mdcd_grp1 1 5 1 0.4077 0.1227 11.03 0.0009

cha2ds2_vasc*mdcd_grp1 1 4 1 0.1903 0.1783 1.14 0.2860

cha2ds2_vasc*mdcd_grp1 1 3 1 -0.0748 0.1062 0.50 0.4813

cha2ds2_vasc*mdcd_grp1 1 2 1 0.1991 0.0896 4.93 0.0263

cha2ds2_vasc*mdcd_grp1 1 1 1 0.3904 2.1296 0.03 0.8545

cost_threshold*mdcd_grp1 1 6 1 0.5422 0.0843 41.39 <.0001

cost_threshold*mdcd_grp1 1 5 1 0.0628 0.0476 1.74 0.1876

cost_threshold*mdcd_grp1 1 4 1 -0.1393 0.0757 3.39 0.0656

cost_threshold*mdcd_grp1 1 3 1 -0.2083 0.0407 26.22 <.0001

cost_threshold*mdcd_grp1 1 2 1 -0.0735 0.0360 4.16 0.0413

cost_threshold*mdcd_grp1 1 1 1 0.0144 0.0586 0.06 0.8054

gndr*mdcd_grp1 1 6 1 0.0436 0.0470 0.86 0.3542

gndr*mdcd_grp1 1 5 1 -0.0553 0.0448 1.52 0.2171

gndr*mdcd_grp1 1 4 1 0.1246 0.0559 4.97 0.0258

gndr*mdcd_grp1 1 3 1 -0.0552 0.0380 2.10 0.1470

gndr*mdcd_grp1 1 2 1 -0.0503 0.0327 2.36 0.1242

gndr*mdcd_grp1 1 1 1 0.0795 0.0582 1.87 0.1718

MMI_1*MMI_2 1 -0.00547 0.00184 8.80 0.0030

MMI_2*age 1 -0.00378 0.000522 52.59 <.0001

MMI_2*stdy_prd 1 0.00155 0.000459 11.41 0.0007

stdy_prd*stdy_prd 1 0.00946 0.000222 1810.00 <.0001

***Note:***

CHF – congestive heart failure

HYP – hypertension

DM – diabetes mellitus

STROKE – includes ischemic stroke, transient ischemic attack, thrombo-embolic event

VD – vascular disease (myocardial infarction, peripheral artery disease)

CAD – coronary artery disease

VALVD - valvular disease

SAPN – chronic sleep apnea

CKD – chronic kidney disease

COPD – chronic obstructive pulmonary disease/bronchiectasis

MBldg – major bleeding (e.g., intracranial and gastro-intestinal)

CogI – cognitive impairment

LIVD – liver disease

DEP – depression

LIPDIS - lipid disorders

SPOND – spondylosis/intervertebral discs,

OSTEO - osteoarthritis

HTHD – hyperthyroidism

MS – metabolic syndrome

Cost_threshold – high risk was considered when the total annual cost prior to the index date for AF or the equivalent date for the non-AF case

GNDR – gender

Age in years

AGE_GRP – age group (18-44 or 0; 45-54 or 1; 55-64 or 2; 65-74 or 3; 75-90 or 4)

Mdcd_grp1 – Medicaid groups (TANF or 0; family care or 1; two groups or 2; ABD nondual or 3; LTSS nondual or 4; ABD duals or 5; LTSS duals or 6)

MMI_1 - first multimorbid index (sum of comorbid history)

MMI_2 – second multimorbid index (sum of comorbid history and age group)

Std_prd – length of enrollment in study (in months)

CHADS_2_, CHA_2_DS_2_-VASc, C_2_HEST – clinical rules as originally defined in the literature (please see text)

**Table S5**

Part a – Model parameters for high risk AF outcome

Standard Wald

Parameter Levels DF Estimate Error Chi-Square Pr > ChiSq

Intercept 1 23.9367 0.7483 1023.34 <.0001

ANEMIA 1 1 0.1653 0.0265 38.77 <.0001

CAD 1 1 0.1665 0.0268 38.69 <.0001

CHF 1 1 0.3978 0.0414 92.38 <.0001

MS 1 1 -0.6644 0.2500 7.06 0.0079

SPOND 1 1 0.6087 0.2506 5.90 0.0151

mdcd_grp1 6 1 0.7233 0.0949 58.07 <.0001

mdcd_grp1 5 1 0.1220 0.0859 2.02 0.1553

mdcd_grp1 4 1 0.0192 0.1333 0.02 0.8856

mdcd_grp1 3 1 -0.4094 0.0930 19.36 <.0001

mdcd_grp1 2 1 0.1535 0.0616 6.21 0.0127

mdcd_grp1 1 1 0.9939 2.7393 0.13 0.7167

stdy_prd 1 -0.8499 0.0250 1156.31 <.0001

ANEMIA*CHF 1 1 1 -0.0835 0.0222 14.07 0.0002

ANEMIA*CKD 1 1 1 0.0605 0.0247 6.00 0.0143

ANEMIA*DEP 1 1 1 -0.0499 0.0202 6.07 0.0137

ANEMIA*DM 1 1 1 -0.0700 0.0203 11.90 0.0006

ASTHMA*LIVD 1 1 1 -0.0531 0.0222 5.75 0.0165

CAD*CKD 1 1 1 -0.0581 0.0251 5.36 0.0206

CHF*DEP 1 1 1 -0.0487 0.0237 4.23 0.0397

CHF*HYP 1 1 1 -0.0953 0.0269 12.55 0.0004

CHF*LIVD 1 1 1 -0.0702 0.0248 8.00 0.0047

CHF*STROKE 1 1 1 -0.0632 0.0306 4.25 0.0392

CHF*age_grp 1 4 1 -0.1002 0.0477 4.41 0.0358

CHF*age_grp 1 3 1 -0.0849 0.0520 2.67 0.1024

CHF*age_grp 1 2 1 0.0410 0.0367 1.25 0.2638

CHF*age_grp 1 1 1 0.00988 0.0400 0.06 0.8052

CKD*COPD 1 1 1 -0.0498 0.0243 4.19 0.0407

CKD*SAPN 1 1 1 -0.1018 0.0301 11.45 0.0007

CKD*SPOND 1 1 1 -0.0791 0.0235 11.30 0.0008

CKD*STROKE 1 1 1 -0.0701 0.0285 6.07 0.0138

CKD*age_grp 1 4 1 -0.2127 0.0522 16.58 <.0001

CKD*age_grp 1 3 1 0.0309 0.0538 0.33 0.5663

CKD*age_grp 1 2 1 0.0406 0.0387 1.10 0.2942

CKD*age_grp 1 1 1 0.0436 0.0416 1.10 0.2949

COGI*COPD 1 1 1 -0.0722 0.0287 6.33 0.0119

COPD*DM 1 1 1 -0.0981 0.0204 23.14 <.0001

COPD*SPOND 1 1 1 -0.0442 0.0192 5.30 0.0213

COPD*STROKE 1 1 1 -0.1015 0.0262 15.05 0.0001

COPD*age_grp 1 4 1 -0.1599 0.0482 11.02 0.0009

COPD*age_grp 1 3 1 0.0188 0.0472 0.16 0.6904

COPD*age_grp 1 2 1 0.0734 0.0333 4.86 0.0274

COPD*age_grp 1 1 1 0.0353 0.0354 0.99 0.3186

DEP*SAPN 1 1 1 -0.0797 0.0261 9.35 0.0022

DEP*SPOND 1 1 1 0.0637 0.0204 9.75 0.0018

DEP*VD 1 1 1 -0.0741 0.0224 10.92 0.0009

DEP*mdcd_grp1 1 6 1 0.0467 0.0525 0.79 0.3744

DEP*mdcd_grp1 1 5 1 -0.0781 0.0619 1.59 0.2069

DEP*mdcd_grp1 1 4 1 -0.1660 0.0566 8.60 0.0034

DEP*mdcd_grp1 1 3 1 -0.0746 0.0441 2.86 0.0908

DEP*mdcd_grp1 1 2 1 -0.0311 0.0370 0.71 0.4001

DEP*mdcd_grp1 1 1 1 0.2122 0.0723 8.62 0.0033

DM*HTHD 1 1 1 -0.0761 0.0299 6.50 0.0108

DM*STROKE 1 1 1 -0.0613 0.0271 5.11 0.0238

DM*mdcd_grp1 1 6 1 -0.1563 0.0476 10.77 0.0010

DM*mdcd_grp1 1 5 1 0.0233 0.0548 0.18 0.6712

DM*mdcd_grp1 1 4 1 -0.0320 0.0554 0.33 0.5631

DM*mdcd_grp1 1 3 1 0.0906 0.0435 4.33 0.0375

DM*mdcd_grp1 1 2 1 0.0404 0.0363 1.24 0.2658

DM*mdcd_grp1 1 1 1 0.000145 0.0794 0.00 0.9985

HTHD*MBldg 1 1 1 -0.1299 0.0267 23.63 <.0001

HYP*SAPN 1 1 1 -0.0995 0.0260 14.61 0.0001

HYP*age_grp 1 4 1 -0.1914 0.0430 19.82 <.0001

HYP*age_grp 1 3 1 -0.0454 0.0503 0.82 0.3665

HYP*age_grp 1 2 1 -0.0707 0.0377 3.52 0.0608

HYP*age_grp 1 1 1 0.1119 0.0395 8.01 0.0046

LIPDIS*age_grp 1 4 1 -0.0186 0.0425 0.19 0.6622

LIPDIS*age_grp 1 3 1 -0.00354 0.0454 0.01 0.9380

LIPDIS*age_grp 1 2 1 -0.0855 0.0329 6.77 0.0093

LIPDIS*age_grp 1 1 1 0.0717 0.0348 4.25 0.0394

LIVD*age_grp 1 4 1 -0.0115 0.0780 0.02 0.8828

LIVD*age_grp 1 3 1 -0.2478 0.0753 10.83 0.0010

LIVD*age_grp 1 2 1 -0.0486 0.0386 1.59 0.2074

LIVD*age_grp 1 1 1 0.1444 0.0378 14.58 0.0001

LIVD*gndr 1 1 1 -0.0446 0.0214 4.34 0.0372

MBldg*VD 1 1 1 -0.0787 0.0245 10.34 0.0013

MS*SPOND 1 1 1 0.5226 0.2500 4.37 0.0365

OSTEO*SAPN 1 1 1 -0.0674 0.0255 6.99 0.0082

OSTEO*VALVD 1 1 1 -0.0635 0.0240 6.99 0.0082

OSTEO*mdcd_grp1 1 6 1 -0.0204 0.0484 0.18 0.6729

OSTEO*mdcd_grp1 1 5 1 0.0518 0.0565 0.84 0.3592

OSTEO*mdcd_grp1 1 4 1 -0.2371 0.0589 16.20 <.0001

OSTEO*mdcd_grp1 1 3 1 -0.0409 0.0443 0.85 0.3555

OSTEO*mdcd_grp1 1 2 1 -0.0521 0.0366 2.02 0.1549

OSTEO*mdcd_grp1 1 1 1 0.2043 0.0740 7.63 0.0058

SAPN*VALVD 1 1 1 -0.1272 0.0294 18.76 <.0001

STROKE*VALVD 1 1 1 -0.0824 0.0283 8.50 0.0035

STROKE*gndr 1 1 1 0.0699 0.0227 9.50 0.0021

VALVD*VD 1 1 1 -0.0716 0.0242 8.71 0.0032

VALVD*gndr 1 1 1 0.0811 0.0228 12.60 0.0004

VD*mdcd_grp1 1 6 1 -0.0732 0.0582 1.58 0.2084

VD*mdcd_grp1 1 5 1 -0.0674 0.0639 1.11 0.2918

VD*mdcd_grp1 1 4 1 -0.1114 0.0632 3.10 0.0783

VD*mdcd_grp1 1 3 1 0.0174 0.0477 0.13 0.7157

VD*mdcd_grp1 1 2 1 0.0533 0.0385 1.91 0.1668

VD*mdcd_grp1 1 1 1 -0.0259 0.0948 0.07 0.7851

age_grp*mdcd_grp1 4 6 1 0.0993 0.1298 0.59 0.4440

age_grp*mdcd_grp1 4 5 1 0.0808 0.1479 0.30 0.5850

age_grp*mdcd_grp1 4 4 1 0.5827 0.3573 2.66 0.1029

age_grp*mdcd_grp1 4 3 1 -0.1415 0.2437 0.34 0.5616

age_grp*mdcd_grp1 4 2 1 0.7148 0.1238 33.32 <.0001

age_grp*mdcd_grp1 4 1 1 1.4004 10.9013 0.02 0.8978

age_grp*mdcd_grp1 3 6 1 -0.0335 0.1220 0.08 0.7835

age_grp*mdcd_grp1 3 5 1 -0.3159 0.1286 6.03 0.0141

age_grp*mdcd_grp1 3 4 1 0.1401 0.3160 0.20 0.6575

age_grp*mdcd_grp1 3 3 1 -0.4139 0.2052 4.07 0.0437

age_grp*mdcd_grp1 3 2 1 0.1583 0.1114 2.02 0.1556

age_grp*mdcd_grp1 3 1 1 0.2619 2.8874 0.01 0.9277

age_grp*mdcd_grp1 2 6 1 0.1014 0.1101 0.85 0.3571

age_grp*mdcd_grp1 2 5 1 0.0138 0.1077 0.02 0.8982

age_grp*mdcd_grp1 2 4 1 -0.1109 0.1402 0.63 0.4287

age_grp*mdcd_grp1 2 3 1 0.2061 0.0986 4.37 0.0366

age_grp*mdcd_grp1 2 2 1 -0.3123 0.0649 23.15 <.0001

age_grp*mdcd_grp1 2 1 1 -0.6410 2.7386 0.05 0.8149

age_grp*mdcd_grp1 1 6 1 -0.1979 0.1548 1.64 0.2009

age_grp*mdcd_grp1 1 5 1 0.2637 0.1189 4.92 0.0265

age_grp*mdcd_grp1 1 4 1 -0.2428 0.1414 2.95 0.0860

age_grp*mdcd_grp1 1 3 1 0.1458 0.1007 2.09 0.1478

age_grp*mdcd_grp1 1 2 1 -0.1444 0.0751 3.70 0.0544

age_grp*mdcd_grp1 1 1 1 -0.5989 2.7392 0.05 0.8269

gndr*mdcd_grp1 1 6 1 -0.0274 0.0409 0.45 0.5033

gndr*mdcd_grp1 1 5 1 -0.0998 0.0527 3.58 0.0584

gndr*mdcd_grp1 1 4 1 0.1403 0.0545 6.62 0.0101

gndr*mdcd_grp1 1 3 1 -0.0210 0.0412 0.26 0.6091

gndr*mdcd_grp1 1 2 1 -0.0631 0.0337 3.50 0.0615

gndr*mdcd_grp1 1 1 1 0.1627 0.0668 5.93 0.0149

age*stdy_prd 1 0.000216 0.000048 20.17 <.0001

stdy_prd*stdy_prd 1 0.00636 0.000214 882.69 <.0001

Part b – Model parameters for stroke associated high risk AF

Standard Wald

Parameter Levels DF Estimate Error Chi-Square Pr > ChiSq

Intercept 1 -4.1846 0.2515 276.73 <.0001

COPD 1 1 -0.0635 0.0231 7.57 0.0059

LIPDIS 1 1 0.0713 0.0142 25.14 <.0001

MBldg 1 1 0.1506 0.0190 62.67 <.0001

SAPN 1 1 -0.0789 0.0317 6.18 0.0129

STROKE 1 1 0.8883 0.0439 409.27 <.0001

age 1 0.1085 0.00896 146.64 <.0001

gndr 1 1 0.1005 0.0215 21.83 <.0001

mdcd_grp1 6 1 0.1819 0.1007 3.26 0.0708

mdcd_grp1 5 1 -0.4047 0.0874 21.45 <.0001

mdcd_grp1 4 1 0.2506 0.1107 5.12 0.0236

mdcd_grp1 3 1 -0.1608 0.0741 4.70 0.0301

mdcd_grp1 2 1 -0.0479 0.0675 0.50 0.4778

mdcd_grp1 1 1 1.2465 1.2165 1.05 0.3055

stdy_grp_5000 1 1 0.1873 0.0397 22.33 <.0001

ANEMIA*DEP 1 1 1 -0.0248 0.0110 5.07 0.0243

ANEMIA*MBldg 1 1 1 -0.0304 0.0127 5.72 0.0168

ANEMIA*STROKE 1 1 1 -0.0595 0.0122 23.69 <.0001

ANEMIA*age_grp 1 4 1 -0.0372 0.0365 1.04 0.3082

ANEMIA*age_grp 1 3 1 -0.0308 0.0350 0.77 0.3794

ANEMIA*age_grp 1 2 1 -0.0259 0.0195 1.76 0.1848

ANEMIA*age_grp 1 1 1 -0.00419 0.0191 0.05 0.8259

ASTHMA*STROKE 1 1 1 -0.0495 0.0131 14.28 0.0002

ASTHMA*gndr 1 1 1 0.0469 0.0129 13.21 0.0003

CAD*STROKE 1 1 1 -0.0332 0.0153 4.71 0.0299

CAD*VALVD 1 1 1 -0.0557 0.0157 12.57 0.0004

CAD*VD 1 1 1 -0.0473 0.0143 11.02 0.0009

CAD*gndr 1 1 1 0.0349 0.0135 6.71 0.0096

CAD*mdcd_grp1 1 6 1 0.0261 0.0477 0.30 0.5836

CAD*mdcd_grp1 1 5 1 0.0365 0.0432 0.72 0.3978

CAD*mdcd_grp1 1 4 1 0.0657 0.0362 3.30 0.0691

CAD*mdcd_grp1 1 3 1 0.00705 0.0278 0.06 0.8000

CAD*mdcd_grp1 1 2 1 -0.0462 0.0257 3.24 0.0720

CAD*mdcd_grp1 1 1 1 -0.1382 0.0696 3.95 0.0470

CAD*stdy_grp_5000 1 1 1 -0.0469 0.0185 6.46 0.0110

CHF*MBldg 1 1 1 0.0354 0.0178 3.98 0.0461

CHF*VD 1 1 1 -0.0698 0.0160 18.96 <.0001

CHF*gndr 1 1 1 0.0342 0.0160 4.59 0.0321

CHF*stdy_grp_5000 1 1 1 -0.0630 0.0187 11.35 0.0008

CKD*LIVD 1 1 1 -0.0391 0.0146 7.14 0.0075

CKD*stdy_grp_5000 1 1 1 -0.0750 0.0168 19.96 <.0001

COGI*DEP 1 1 1 -0.0603 0.0156 15.00 0.0001

COGI*VALVD 1 1 1 -0.0475 0.0203 5.45 0.0195

COGI*age_grp 1 4 1 -0.1748 0.0561 9.71 0.0018

COGI*age_grp 1 3 1 -0.0576 0.0638 0.81 0.3668

COGI*age_grp 1 2 1 0.0807 0.0399 4.08 0.0434

COGI*age_grp 1 1 1 0.0523 0.0439 1.42 0.2335

COGI*stdy_grp_5000 1 1 1 -0.0950 0.0308 9.49 0.0021

COPD*DEP 1 1 1 -0.0232 0.0112 4.34 0.0372

COPD*STROKE 1 1 1 -0.0446 0.0136 10.70 0.0011

COPD*stdy_grp_5000 1 1 1 -0.0903 0.0214 17.82 <.0001

DEP*HYP 1 1 1 -0.0234 0.0111 4.43 0.0354

DEP*mdcd_grp1 1 6 1 0.0413 0.0432 0.91 0.3394

DEP*mdcd_grp1 1 5 1 -0.0612 0.0420 2.12 0.1452

DEP*mdcd_grp1 1 4 1 -0.0184 0.0293 0.40 0.5295

DEP*mdcd_grp1 1 3 1 -0.00216 0.0237 0.01 0.9272

DEP*mdcd_grp1 1 2 1 0.0476 0.0218 4.76 0.0292

DEP*mdcd_grp1 1 1 1 0.0655 0.0530 1.53 0.2164

DM*LIPDIS 1 1 1 -0.0313 0.0112 7.73 0.0054

DM*OSTEO 1 1 1 -0.0436 0.0107 16.58 <.0001

DM*SAPN 1 1 1 -0.0578 0.0155 13.98 0.0002

DM*STROKE 1 1 1 -0.0741 0.0128 33.64 <.0001

DM*mdcd_grp1 1 6 1 -0.2166 0.0393 30.37 <.0001

DM*mdcd_grp1 1 5 1 -0.0405 0.0378 1.15 0.2840

DM*mdcd_grp1 1 4 1 0.0411 0.0291 2.00 0.1577

DM*mdcd_grp1 1 3 1 0.0292 0.0233 1.58 0.2092

DM*mdcd_grp1 1 2 1 0.0797 0.0214 13.86 0.0002

DM*mdcd_grp1 1 1 1 0.0371 0.0517 0.51 0.4731

HTHD*HYP 1 1 1 -0.0572 0.0191 8.95 0.0028

HTHD*age_grp 1 4 1 -0.2437 0.1501 2.64 0.1045

HTHD*age_grp 1 3 1 0.1870 0.1275 2.15 0.1424

HTHD*age_grp 1 2 1 0.1355 0.0496 7.47 0.0063

HTHD*age_grp 1 1 1 0.1021 0.0480 4.53 0.0333

HYP*SPOND 1 1 1 -0.0210 0.00964 4.73 0.0296

HYP*STROKE 1 1 1 -0.0595 0.0145 16.84 <.0001

HYP*VD 1 1 1 -0.0347 0.0152 5.22 0.0223

HYP*age_grp 1 4 1 -0.0413 0.0356 1.35 0.2454

HYP*age_grp 1 3 1 -0.0914 0.0326 7.86 0.0051

HYP*age_grp 1 2 1 -0.0270 0.0200 1.82 0.1769

HYP*age_grp 1 1 1 0.0435 0.0193 5.10 0.0239

LIPDIS*STROKE 1 1 1 0.0470 0.0128 13.52 0.0002

LIPDIS*gndr 1 1 1 0.0338 0.00964 12.27 0.0005

LIVD*STROKE 1 1 1 -0.0714 0.0139 26.23 <.0001

LIVD*mdcd_grp1 1 6 1 0.0361 0.0682 0.28 0.5962

LIVD*mdcd_grp1 1 5 1 0.0144 0.0517 0.08 0.7802

LIVD*mdcd_grp1 1 4 1 -0.0923 0.0334 7.63 0.0057

LIVD*mdcd_grp1 1 3 1 -0.00420 0.0266 0.02 0.8744

LIVD*mdcd_grp1 1 2 1 -0.0423 0.0251 2.85 0.0916

LIVD*mdcd_grp1 1 1 1 0.0338 0.0560 0.36 0.5463

OSTEO*age_grp 1 4 1 -0.0943 0.0345 7.45 0.0063

OSTEO*age_grp 1 3 1 -0.00222 0.0328 0.00 0.9460

OSTEO*age_grp 1 2 1 0.00339 0.0172 0.04 0.8440

OSTEO*age_grp 1 1 1 0.0131 0.0176 0.55 0.4587

SAPN*STROKE 1 1 1 0.0711 0.0303 5.53 0.0187

SAPN*VD 1 1 1 -0.0978 0.0199 24.05 <.0001

SPOND*STROKE 1 1 1 -0.0607 0.0113 28.81 <.0001

SPOND*gndr 1 1 1 0.0275 0.00938 8.61 0.0033

SPOND*mdcd_grp1 1 6 1 -0.1083 0.0410 6.96 0.0083

SPOND*mdcd_grp1 1 5 1 0.0654 0.0349 3.51 0.0609

SPOND*mdcd_grp1 1 4 1 -0.0693 0.0280 6.12 0.0133

SPOND*mdcd_grp1 1 3 1 -0.0556 0.0217 6.56 0.0104

SPOND*mdcd_grp1 1 2 1 0.0172 0.0196 0.76 0.3820

SPOND*mdcd_grp1 1 1 1 0.0875 0.0432 4.09 0.0431

SPOND*stdy_grp_5000 1 1 1 -0.0410 0.0136 9.09 0.0026

STROKE*VALVD 1 1 1 -0.0505 0.0166 9.19 0.0024

STROKE*VD 1 1 1 -0.0957 0.0142 45.23 <.0001

STROKE*age_grp 1 4 1 -0.1374 0.0422 10.60 0.0011

STROKE*age_grp 1 3 1 0.0110 0.0382 0.08 0.7724

STROKE*age_grp 1 2 1 -0.0575 0.0228 6.35 0.0118

STROKE*age_grp 1 1 1 0.0146 0.0241 0.37 0.5455

STROKE*mdcd_grp1 1 6 1 0.3247 0.0440 54.49 <.0001

STROKE*mdcd_grp1 1 5 1 -0.1133 0.0462 6.01 0.0142

STROKE*mdcd_grp1 1 4 1 0.1043 0.0340 9.39 0.0022

STROKE*mdcd_grp1 1 3 1 -0.1054 0.0272 14.97 0.0001

STROKE*mdcd_grp1 1 2 1 -0.0445 0.0241 3.41 0.0647

STROKE*mdcd_grp1 1 1 1 -0.0150 0.0587 0.07 0.7979

STROKE*stdy_grp_5000 1 1 1 -0.1178 0.0259 20.61 <.0001

VALVD*age_grp 1 4 1 -0.0932 0.0547 2.90 0.0887

VALVD*age_grp 1 3 1 0.0154 0.0505 0.09 0.7604

VALVD*age_grp 1 2 1 -0.0477 0.0276 2.98 0.0845

VALVD*age_grp 1 1 1 0.0139 0.0281 0.24 0.6224

VD*age_grp 1 4 1 -0.0787 0.0411 3.67 0.0554

VD*age_grp 1 3 1 -0.1028 0.0392 6.86 0.0088

VD*age_grp 1 2 1 0.0221 0.0217 1.03 0.3094

VD*age_grp 1 1 1 0.0319 0.0224 2.02 0.1548

age_grp*gndr 4 1 1 0.0797 0.0370 4.64 0.0313

age_grp*gndr 3 1 1 -0.0651 0.0319 4.18 0.0409

age_grp*gndr 2 1 1 -0.0716 0.0190 14.27 0.0002

age_grp*gndr 1 1 1 -0.0230 0.0198 1.35 0.2449

age_grp*mdcd_grp1 4 6 1 -0.7498 0.1992 14.17 0.0002

age_grp*mdcd_grp1 4 5 1 -0.4252 0.2038 4.35 0.0369

age_grp*mdcd_grp1 4 4 1 -0.2213 0.3249 0.46 0.4958

age_grp*mdcd_grp1 4 3 1 -0.5733 0.2169 6.99 0.0082

age_grp*mdcd_grp1 4 2 1 -0.1695 0.1954 0.75 0.3858

age_grp*mdcd_grp1 4 1 1 5.9009 4.7986 1.51 0.2188

age_grp*mdcd_grp1 3 6 1 0.0859 0.1512 0.32 0.5698

age_grp*mdcd_grp1 3 5 1 0.1873 0.1510 1.54 0.2147

age_grp*mdcd_grp1 3 4 1 -0.4991 0.2714 3.38 0.0659

age_grp*mdcd_grp1 3 3 1 -0.1093 0.1612 0.46 0.4977

age_grp*mdcd_grp1 3 2 1 0.2206 0.1451 2.31 0.1284

age_grp*mdcd_grp1 3 1 1 -1.3891 1.4585 0.91 0.3409

age_grp*mdcd_grp1 2 6 1 0.1883 0.0853 4.88 0.0272

age_grp*mdcd_grp1 2 5 1 0.1374 0.0820 2.81 0.0938

age_grp*mdcd_grp1 2 4 1 0.2695 0.1102 5.98 0.0145

age_grp*mdcd_grp1 2 3 1 0.2005 0.0721 7.73 0.0054

age_grp*mdcd_grp1 2 2 1 -0.0397 0.0616 0.42 0.5188

age_grp*mdcd_grp1 2 1 1 -1.5252 1.2145 1.58 0.2092

age_grp*mdcd_grp1 1 6 1 0.1383 0.1046 1.75 0.1860

age_grp*mdcd_grp1 1 5 1 0.1091 0.0910 1.44 0.2308

age_grp*mdcd_grp1 1 4 1 0.2055 0.1093 3.54 0.0600

age_grp*mdcd_grp1 1 3 1 0.2284 0.0712 10.31 0.0013

age_grp*mdcd_grp1 1 2 1 0.0812 0.0666 1.49 0.2229

age_grp*mdcd_grp1 1 1 1 -1.4933 1.2153 1.51 0.2192

gndr*mdcd_grp1 1 6 1 0.00747 0.0362 0.04 0.8368

gndr*mdcd_grp1 1 5 1 -0.0318 0.0360 0.78 0.3772

gndr*mdcd_grp1 1 4 1 0.00615 0.0282 0.05 0.8273

gndr*mdcd_grp1 1 3 1 0.000712 0.0219 0.00 0.9740

gndr*mdcd_grp1 1 2 1 -0.0405 0.0200 4.09 0.0430

gndr*mdcd_grp1 1 1 1 -0.00986 0.0440 0.05 0.8224

age*age 1 -0.00069 0.000073 90.31 <.0001

age*stdy_prd 1 -0.00031 0.000085 13.71 0.0002

stdy_prd*stdy_prd 1 0.000178 0.000040 19.81 <.0001

Part c – Model parameters for heart failure associated high risk AF

Standard Wald

Parameter Levels DF Estimate Error Chi-Square Pr > ChiSq

Intercept 1 -3.1905 0.4491 50.47 <.0001

CAD 1 1 0.1608 0.0181 79.02 <.0001

CHF 1 1 1.0400 0.0342 927.35 <.0001

CKD 1 1 0.2088 0.0287 53.04 <.0001

DEP 1 1 -0.0966 0.0253 14.59 0.0001

DM 1 1 0.0742 0.0201 13.62 0.0002

VALVD 1 1 0.1731 0.0214 65.25 <.0001

age 1 0.0872 0.00900 93.78 <.0001

age_grp 4 1 0.0162 2.7920 0.00 0.9954

age_grp 3 1 -1.2170 2.6519 0.21 0.6463

age_grp 2 1 0.0677 0.2346 0.08 0.7729

age_grp 1 1 0.1916 0.2370 0.65 0.4190

mdcd_grp1 6 1 0.3946 0.2429 2.64 0.1043

mdcd_grp1 5 1 0.1943 0.2379 0.67 0.4142

mdcd_grp1 4 1 0.5807 0.2432 5.70 0.0169

mdcd_grp1 3 1 0.1867 0.2336 0.64 0.4242

mdcd_grp1 2 1 0.2837 0.2315 1.50 0.2203

mdcd_grp1 1 1 -0.3748 0.1347 7.75 0.0054

stdy_grp_5000 1 1 0.4841 0.0396 149.11 <.0001

stdy_prd 1 0.0124 0.00519 5.68 0.0171

ANEMIA*CHF 1 1 1 -0.1127 0.0124 82.54 <.0001

ANEMIA*CKD 1 1 1 0.0546 0.0132 17.06 <.0001

ANEMIA*COPD 1 1 1 -0.0330 0.0115 8.22 0.0041

ANEMIA*HYP 1 1 1 -0.0244 0.0124 3.87 0.0490

ANEMIA*MBldg 1 1 1 0.0277 0.0140 3.91 0.0480

ANEMIA*stdy_grp_5000 1 1 1 -0.0799 0.0160 24.99 <.0001

ASTHMA*DM 1 1 1 -0.0310 0.0129 5.81 0.0159

ASTHMA*VALVD 1 1 1 -0.0351 0.0143 6.04 0.0140

ASTHMA*VD 1 1 1 -0.0518 0.0136 14.54 0.0001

CAD*CHF 1 1 1 -0.0763 0.0143 28.62 <.0001

CAD*COPD 1 1 1 -0.0400 0.0123 10.55 0.0012

CAD*LIPDIS 1 1 1 0.0376 0.0134 7.95 0.0048

CAD*VALVD 1 1 1 -0.0497 0.0158 9.88 0.0017

CHF*COPD 1 1 1 -0.0459 0.0123 13.98 0.0002

CHF*DM 1 1 1 -0.1162 0.0128 82.65 <.0001

CHF*HYP 1 1 1 -0.0702 0.0147 22.84 <.0001

CHF*LIPDIS 1 1 1 0.0417 0.0127 10.76 0.0010

CHF*MBldg 1 1 1 -0.0464 0.0169 7.53 0.0061

CHF*STROKE 1 1 1 -0.0840 0.0170 24.44 <.0001

CHF*VD 1 1 1 -0.0656 0.0140 22.09 <.0001

CHF*age_grp 1 4 1 -0.1487 0.0363 16.81 <.0001

CHF*age_grp 1 3 1 -0.0975 0.0346 7.95 0.0048

CHF*age_grp 1 2 1 -0.0208 0.0222 0.88 0.3483

CHF*age_grp 1 1 1 0.0232 0.0235 0.97 0.3235

CHF*mdcd_grp1 1 6 1 0.0383 0.0397 0.93 0.3357

CHF*mdcd_grp1 1 5 1 -0.0533 0.0391 1.86 0.1726

CHF*mdcd_grp1 1 4 1 0.0516 0.0351 2.17 0.1409

CHF*mdcd_grp1 1 3 1 0.0416 0.0271 2.36 0.1242

CHF*mdcd_grp1 1 2 1 -0.0365 0.0241 2.28 0.1308

CHF*mdcd_grp1 1 1 1 -0.1978 0.0700 8.00 0.0047

CHF*stdy_grp_5000 1 1 1 -0.0836 0.0247 11.47 0.0007

CKD*COPD 1 1 1 -0.0578 0.0150 14.79 0.0001

CKD*DEP 1 1 1 -0.0716 0.0156 20.97 <.0001

CKD*HYP 1 1 1 -0.0479 0.0183 6.87 0.0087

CKD*LIVD 1 1 1 -0.0452 0.0134 11.45 0.0007

CKD*OSTEO 1 1 1 -0.0364 0.0109 11.08 0.0009

CKD*age_grp 1 4 1 -0.1624 0.0404 16.18 <.0001

CKD*age_grp 1 3 1 -0.0334 0.0382 0.76 0.3826

CKD*age_grp 1 2 1 -0.0363 0.0248 2.14 0.1437

CKD*age_grp 1 1 1 0.0456 0.0273 2.79 0.0950

CKD*mdcd_grp1 1 6 1 -0.1237 0.0481 6.61 0.0102

CKD*mdcd_grp1 1 5 1 0.0524 0.0404 1.68 0.1948

CKD*mdcd_grp1 1 4 1 -0.00782 0.0386 0.04 0.8394

CKD*mdcd_grp1 1 3 1 0.0389 0.0315 1.53 0.2165

CKD*mdcd_grp1 1 2 1 0.0765 0.0281 7.41 0.0065

CKD*mdcd_grp1 1 1 1 -0.0347 0.0875 0.16 0.6920

COGI*LIPDIS 1 1 1 0.0363 0.0153 5.64 0.0176

COGI*SPOND 1 1 1 0.0358 0.0158 5.15 0.0233

COPD*MS 1 1 1 -0.0964 0.0193 24.83 <.0001

COPD*age_grp 1 4 1 0.0130 0.0362 0.13 0.7201

COPD*age_grp 1 3 1 -0.0148 0.0328 0.20 0.6507

COPD*age_grp 1 2 1 0.0531 0.0198 7.21 0.0073

COPD*age_grp 1 1 1 0.0172 0.0210 0.67 0.4130

COPD*gndr 1 1 1 0.0313 0.00965 10.54 0.0012

COPD*mdcd_grp1 1 6 1 0.0757 0.0395 3.67 0.0554

COPD*mdcd_grp1 1 5 1 -0.0365 0.0343 1.13 0.2874

COPD*mdcd_grp1 1 4 1 0.0238 0.0297 0.64 0.4225

COPD*mdcd_grp1 1 3 1 -0.0618 0.0232 7.09 0.0077

COPD*mdcd_grp1 1 2 1 0.00961 0.0214 0.20 0.6531

COPD*mdcd_grp1 1 1 1 -0.0246 0.0576 0.18 0.6688

DEP*mdcd_grp1 1 6 1 -0.0428 0.0408 1.10 0.2935

DEP*mdcd_grp1 1 5 1 -0.0440 0.0370 1.41 0.2345

DEP*mdcd_grp1 1 4 1 -0.0129 0.0294 0.19 0.6602

DEP*mdcd_grp1 1 3 1 -0.0783 0.0239 10.73 0.0011

DEP*mdcd_grp1 1 2 1 0.0261 0.0222 1.38 0.2399

DEP*mdcd_grp1 1 1 1 0.1119 0.0584 3.67 0.0554

DEP*stdy_grp_5000 1 1 1 -0.0869 0.0215 16.33 <.0001

DM*VALVD 1 1 1 -0.0470 0.0156 9.11 0.0025

DM*age_grp 1 4 1 -0.0519 0.0323 2.58 0.1079

DM*age_grp 1 3 1 -0.1412 0.0299 22.35 <.0001

DM*age_grp 1 2 1 0.00565 0.0188 0.09 0.7641

DM*age_grp 1 1 1 0.0539 0.0192 7.90 0.0049

DM*gndr 1 1 1 0.0336 0.00967 12.05 0.0005

HYP*LIVD 1 1 1 -0.0333 0.0134 6.14 0.0132

HYP*age_grp 1 4 1 -0.1083 0.0337 10.33 0.0013

HYP*age_grp 1 3 1 -0.1588 0.0317 25.14 <.0001

HYP*age_grp 1 2 1 -0.00134 0.0219 0.00 0.9512

HYP*age_grp 1 1 1 0.0448 0.0225 3.96 0.0467

HYP*mdcd_grp1 1 6 1 -0.0905 0.0339 7.13 0.0076

HYP*mdcd_grp1 1 5 1 0.0289 0.0349 0.69 0.4073

HYP*mdcd_grp1 1 4 1 0.0440 0.0359 1.50 0.2205

HYP*mdcd_grp1 1 3 1 -0.0151 0.0251 0.36 0.5488

HYP*mdcd_grp1 1 2 1 -0.00218 0.0221 0.01 0.9215

HYP*mdcd_grp1 1 1 1 -0.0432 0.0520 0.69 0.4056

HYP*stdy_grp_5000 1 1 1 -0.0858 0.0191 20.23 <.0001

LIPDIS*LIVD 1 1 1 -0.0333 0.0124 7.18 0.0074

MBldg*age_grp 1 4 1 -0.1741 0.0586 8.81 0.0030

MBldg*age_grp 1 3 1 0.0873 0.0502 3.03 0.0816

MBldg*age_grp 1 2 1 0.0387 0.0297 1.70 0.1925

MBldg*age_grp 1 1 1 -0.0160 0.0303 0.28 0.5984

MBldg*mdcd_grp1 1 6 1 0.2551 0.0648 15.50 <.0001

MBldg*mdcd_grp1 1 5 1 -0.0259 0.0542 0.23 0.6333

MBldg*mdcd_grp1 1 4 1 -0.0802 0.0417 3.70 0.0544

MBldg*mdcd_grp1 1 3 1 -0.0519 0.0333 2.44 0.1185

MBldg*mdcd_grp1 1 2 1 -0.1107 0.0301 13.55 0.0002

MBldg*mdcd_grp1 1 1 1 0.0475 0.0692 0.47 0.4922

MBldg*stdy_grp_5000 1 1 1 -0.0540 0.0211 6.54 0.0105

OSTEO*SPOND 1 1 1 0.0222 0.0108 4.22 0.0401

SAPN*stdy_grp_5000 1 1 1 -0.1456 0.0227 41.10 <.0001

SPOND*VALVD 1 1 1 -0.0457 0.0137 11.09 0.0009

SPOND*age_grp 1 4 1 -0.0363 0.0330 1.21 0.2719

SPOND*age_grp 1 3 1 -0.0168 0.0295 0.32 0.5694

SPOND*age_grp 1 2 1 -0.0351 0.0178 3.89 0.0485

SPOND*age_grp 1 1 1 0.00238 0.0180 0.02 0.8946

STROKE*age_grp 1 4 1 -0.0442 0.0467 0.89 0.3446

STROKE*age_grp 1 3 1 -0.0336 0.0468 0.52 0.4730

STROKE*age_grp 1 2 1 -0.0496 0.0267 3.44 0.0638

STROKE*age_grp 1 1 1 0.0510 0.0283 3.25 0.0714

VALVD*age_grp 1 4 1 -0.0105 0.0471 0.05 0.8243

VALVD*age_grp 1 3 1 -0.0509 0.0463 1.21 0.2711

VALVD*age_grp 1 2 1 -0.0339 0.0271 1.56 0.2110

VALVD*age_grp 1 1 1 -0.0164 0.0279 0.35 0.5555

VD*age_grp 1 4 1 -0.0792 0.0374 4.50 0.0339

VD*age_grp 1 3 1 -0.0849 0.0363 5.47 0.0194

VD*age_grp 1 2 1 0.0260 0.0214 1.48 0.2241

VD*age_grp 1 1 1 0.0137 0.0226 0.37 0.5443

VD*stdy_grp_5000 1 1 1 -0.0853 0.0166 26.38 <.0001

age_grp*mdcd_grp1 4 6 1 -0.5955 2.7903 0.05 0.8310

age_grp*mdcd_grp1 4 5 1 -0.4856 2.7903 0.03 0.8618

age_grp*mdcd_grp1 4 4 1 -0.6069 2.7975 0.05 0.8283

age_grp*mdcd_grp1 4 3 1 -0.6134 2.7910 0.05 0.8260

age_grp*mdcd_grp1 4 2 1 -0.2472 2.7901 0.01 0.9294

age_grp*mdcd_grp1 4 1 1 5.9891 15.8405 0.14 0.7054

age_grp*mdcd_grp1 3 6 1 0.9002 2.6513 0.12 0.7342

age_grp*mdcd_grp1 3 5 1 0.9498 2.6513 0.13 0.7202

age_grp*mdcd_grp1 3 4 1 1.1334 2.6562 0.18 0.6696

age_grp*mdcd_grp1 3 3 1 0.6067 2.6519 0.05 0.8190

age_grp*mdcd_grp1 3 2 1 1.1472 2.6512 0.19 0.6652

age_grp*mdcd_grp1 3 1 1 -5.7133 15.8387 0.13 0.7183

age_grp*mdcd_grp1 2 6 1 -0.0861 0.2371 0.13 0.7164

age_grp*mdcd_grp1 2 5 1 -0.0666 0.2338 0.08 0.7757

age_grp*mdcd_grp1 2 4 1 -0.2028 0.2399 0.71 0.3980

age_grp*mdcd_grp1 2 3 1 0.0584 0.2313 0.06 0.8007

age_grp*mdcd_grp1 2 2 1 -0.3368 0.2299 2.15 0.1429

age_grp*mdcd_grp1 2 1 1 -0.1189 0.1153 1.06 0.3026

age_grp*mdcd_grp1 1 6 1 -0.0920 0.2417 0.14 0.7035

age_grp*mdcd_grp1 1 5 1 -0.2625 0.2353 1.24 0.2646

age_grp*mdcd_grp1 1 4 1 -0.1578 0.2391 0.44 0.5094

age_grp*mdcd_grp1 1 3 1 -0.0534 0.2309 0.05 0.8170

age_grp*mdcd_grp1 1 2 1 -0.2422 0.2299 1.11 0.2922

age_grp*mdcd_grp1 1 1 0 0 . . .

age_grp*stdy_grp_5000 4 1 1 -0.1468 0.0400 13.48 0.0002

age_grp*stdy_grp_5000 3 1 1 -0.0330 0.0442 0.56 0.4549

age_grp*stdy_grp_5000 2 1 1 0.0400 0.0343 1.36 0.2439

age_grp*stdy_grp_5000 1 1 1 -0.0691 0.0375 3.39 0.0657

gndr*mdcd_grp1 1 6 1 0.0755 0.0284 7.07 0.0078

gndr*mdcd_grp1 1 5 1 0.00819 0.0298 0.08 0.7837

gndr*mdcd_grp1 1 4 1 0.0139 0.0272 0.26 0.6082

gndr*mdcd_grp1 1 3 1 0.00919 0.0196 0.22 0.6389

gndr*mdcd_grp1 1 2 1 0.0158 0.0178 0.79 0.3754

gndr*mdcd_grp1 1 1 1 -0.0639 0.0417 2.34 0.1259

age*age 1 -0.00038 0.000068 30.91 <.0001

age*stdy_prd 1 -0.00038 0.000089 18.02 <.0001

Part d – Model parameters for myocardial infarction associated high risk AF

Standard Wald

Parameter Levels DF Estimate Error Chi-Square Pr > ChiSq

Intercept 1 -7.2277 0.9029 64.08 <.0001

CAD 1 1 0.2623 0.0327 64.23 <.0001

CHF 1 1 0.2425 0.0299 65.88 <.0001

CKD 1 1 0.1665 0.0289 33.15 <.0001

HYP 1 1 -0.1282 0.0408 9.90 0.0017

LIVD 1 1 0.0989 0.0263 14.14 0.0002

MBldg 1 1 0.1051 0.0238 19.55 <.0001

VALVD 1 1 0.1428 0.0194 53.93 <.0001

VD 1 1 0.6458 0.0706 83.69 <.0001

age 1 0.1609 0.0122 173.75 <.0001

mdcd_grp1 6 1 -0.3976 0.1288 9.54 0.0020

mdcd_grp1 5 1 0.2093 0.0868 5.82 0.0159

mdcd_grp1 4 1 0.2726 0.1237 4.86 0.0275

mdcd_grp1 3 1 0.3771 0.0746 25.51 <.0001

mdcd_grp1 2 1 0.2461 0.0659 13.95 0.0002

mdcd_grp1 1 1 0.1951 1.5314 0.02 0.8986

stdy_grp_5000 1 1 0.6043 0.0560 116.43 <.0001

stdy_prd 1 0.0759 0.0248 9.39 0.0022

ANEMIA*CAD 1 1 1 -0.0488 0.0151 10.50 0.0012

ANEMIA*LIPDIS 1 1 1 -0.0329 0.0132 6.22 0.0126

ANEMIA*VD 1 1 1 -0.0357 0.0146 6.01 0.0142

ANEMIA*mdcd_grp1 1 6 1 0.1612 0.0577 7.81 0.0052

ANEMIA*mdcd_grp1 1 5 1 0.0973 0.0436 4.97 0.0258

ANEMIA*mdcd_grp1 1 4 1 -0.0231 0.0371 0.39 0.5333

ANEMIA*mdcd_grp1 1 3 1 -0.0252 0.0259 0.95 0.3303

ANEMIA*mdcd_grp1 1 2 1 -0.0137 0.0240 0.32 0.5689

ANEMIA*mdcd_grp1 1 1 1 -0.1171 0.0574 4.16 0.0413

ASTHMA*HYP 1 1 1 -0.0554 0.0157 12.42 0.0004

ASTHMA*SAPN 1 1 1 -0.0743 0.0159 21.81 <.0001

CAD*CHF 1 1 1 -0.0860 0.0158 29.65 <.0001

CAD*CKD 1 1 1 -0.0380 0.0186 4.19 0.0406

CAD*COPD 1 1 1 -0.0725 0.0127 32.77 <.0001

CAD*DM 1 1 1 -0.0654 0.0141 21.58 <.0001

CAD*LIPDIS 1 1 1 0.1191 0.0151 62.24 <.0001

CAD*STROKE 1 1 1 -0.0735 0.0198 13.72 0.0002

CAD*VALVD 1 1 1 -0.0681 0.0174 15.31 <.0001

CAD*VD 1 1 1 0.1113 0.0139 63.73 <.0001

CAD*age_grp 1 4 1 -0.1086 0.0471 5.31 0.0212

CAD*age_grp 1 3 1 -0.0861 0.0423 4.13 0.0421

CAD*age_grp 1 2 1 -0.0367 0.0244 2.27 0.1320

CAD*age_grp 1 1 1 0.1080 0.0246 19.32 <.0001

CAD*gndr 1 1 1 0.0235 0.0120 3.85 0.0498

CAD*stdy_grp_5000 1 1 1 -0.1257 0.0245 26.24 <.0001

CHF*COPD 1 1 1 -0.0560 0.0149 14.13 0.0002

CHF*HYP 1 1 1 -0.0512 0.0241 4.52 0.0336

CHF*stdy_grp_5000 1 1 1 -0.0645 0.0233 7.69 0.0056

CKD*HYP 1 1 1 -0.0648 0.0258 6.31 0.0120

CKD*VD 1 1 1 -0.0412 0.0184 5.02 0.0250

CKD*age_grp 1 4 1 -0.1288 0.0520 6.14 0.0132

CKD*age_grp 1 3 1 -0.0652 0.0477 1.87 0.1717

CKD*age_grp 1 2 1 0.0143 0.0278 0.27 0.6063

CKD*age_grp 1 1 1 0.0255 0.0291 0.77 0.3817

COGI*DEP 1 1 1 -0.0691 0.0236 8.56 0.0034

COGI*DM 1 1 1 -0.0711 0.0204 12.19 0.0005

COPD*HTHD 1 1 1 -0.0573 0.0185 9.56 0.0020

COPD*gndr 1 1 1 0.0414 0.0115 12.88 0.0003

COPD*mdcd_grp1 1 6 1 -0.0589 0.0605 0.95 0.3308

COPD*mdcd_grp1 1 5 1 -0.0117 0.0417 0.08 0.7796

COPD*mdcd_grp1 1 4 1 -0.00388 0.0356 0.01 0.9132

COPD*mdcd_grp1 1 3 1 -0.0347 0.0256 1.83 0.1762

COPD*mdcd_grp1 1 2 1 0.0739 0.0252 8.56 0.0034

COPD*mdcd_grp1 1 1 1 -0.0250 0.0607 0.17 0.6804

DEP*LIVD 1 1 1 0.0413 0.0143 8.35 0.0039

DEP*VALVD 1 1 1 0.0384 0.0167 5.30 0.0214

DEP*mdcd_grp1 1 6 1 -0.0290 0.0680 0.18 0.6701

DEP*mdcd_grp1 1 5 1 -0.00244 0.0462 0.00 0.9578

DEP*mdcd_grp1 1 4 1 -0.1113 0.0366 9.24 0.0024

DEP*mdcd_grp1 1 3 1 -0.0350 0.0270 1.68 0.1955

DEP*mdcd_grp1 1 2 1 -0.0215 0.0263 0.67 0.4137

DEP*mdcd_grp1 1 1 1 0.1383 0.0590 5.49 0.0192

DEP*stdy_grp_5000 1 1 1 -0.0716 0.0218 10.77 0.0010

DM*HYP 1 1 1 -0.0469 0.0172 7.45 0.0063

DM*OSTEO 1 1 1 -0.0384 0.0126 9.25 0.0024

DM*VD 1 1 1 -0.0704 0.0142 24.47 <.0001

DM*gndr 1 1 1 0.0489 0.0117 17.38 <.0001

DM*mdcd_grp1 1 6 1 -0.1738 0.0593 8.58 0.0034

DM*mdcd_grp1 1 5 1 -0.00822 0.0413 0.04 0.8422

DM*mdcd_grp1 1 4 1 0.0406 0.0361 1.27 0.2606

DM*mdcd_grp1 1 3 1 -0.0113 0.0267 0.18 0.6728

DM*mdcd_grp1 1 2 1 0.0703 0.0253 7.70 0.0055

DM*mdcd_grp1 1 1 1 0.0363 0.0590 0.38 0.5386

HTHD*LIPDIS 1 1 1 0.0965 0.0220 19.19 <.0001

HTHD*stdy_grp_5000 1 1 1 0.1159 0.0444 6.82 0.0090

HYP*VD 1 1 1 -0.0812 0.0171 22.62 <.0001

HYP*age_grp 1 4 1 -0.1081 0.0500 4.66 0.0308

HYP*age_grp 1 3 1 -0.1458 0.0449 10.55 0.0012

HYP*age_grp 1 2 1 -0.0220 0.0257 0.73 0.3923

HYP*age_grp 1 1 1 0.0228 0.0247 0.85 0.3559

HYP*stdy_grp_5000 1 1 1 -0.0893 0.0297 9.02 0.0027

LIPDIS*LIVD 1 1 1 -0.0317 0.0143 4.89 0.0271

LIPDIS*VALVD 1 1 1 -0.0574 0.0181 10.07 0.0015

LIPDIS*VD 1 1 1 -0.0406 0.0148 7.56 0.0060

LIPDIS*mdcd_grp1 1 6 1 0.0530 0.0550 0.93 0.3346

LIPDIS*mdcd_grp1 1 5 1 -0.0559 0.0396 2.00 0.1575

LIPDIS*mdcd_grp1 1 4 1 0.0910 0.0366 6.18 0.0129

LIPDIS*mdcd_grp1 1 3 1 -0.0589 0.0251 5.50 0.0191

LIPDIS*mdcd_grp1 1 2 1 0.0186 0.0245 0.58 0.4473

LIPDIS*mdcd_grp1 1 1 1 -0.1303 0.0515 6.40 0.0114

LIVD*VD 1 1 1 0.0413 0.0148 7.81 0.0052

LIVD*age_grp 1 4 1 0.0264 0.0767 0.12 0.7312

LIVD*age_grp 1 3 1 -0.00092 0.0597 0.00 0.9877

LIVD*age_grp 1 2 1 -0.0552 0.0307 3.24 0.0719

LIVD*age_grp 1 1 1 -0.0383 0.0305 1.58 0.2091

MBldg*mdcd_grp1 1 6 1 0.2039 0.0878 5.40 0.0201

MBldg*mdcd_grp1 1 5 1 0.0238 0.0623 0.15 0.7021

MBldg*mdcd_grp1 1 4 1 -0.0486 0.0486 1.00 0.3175

MBldg*mdcd_grp1 1 3 1 -0.00264 0.0366 0.01 0.9427

MBldg*mdcd_grp1 1 2 1 -0.1070 0.0366 8.56 0.0034

MBldg*mdcd_grp1 1 1 1 -0.00472 0.0769 0.00 0.9510

MS*SAPN 1 1 1 0.0817 0.0266 9.43 0.0021

MS*VD 1 1 1 0.1390 0.0618 5.06 0.0245

OSTEO*VALVD 1 1 1 0.0279 0.0129 4.70 0.0301

SPOND*VD 1 1 1 -0.0393 0.0116 11.47 0.0007

SPOND*stdy_grp_5000 1 1 1 -0.0471 0.0118 15.84 <.0001

STROKE*VD 1 1 1 -0.0392 0.0198 3.90 0.0484

STROKE*gndr 1 1 1 0.0464 0.0148 9.83 0.0017

VD*age_grp 1 4 1 -0.2143 0.0511 17.60 <.0001

VD*age_grp 1 3 1 -0.0781 0.0450 3.00 0.0831

VD*age_grp 1 2 1 0.0149 0.0256 0.34 0.5619

VD*age_grp 1 1 1 0.0224 0.0267 0.70 0.4024

VD*mdcd_grp1 1 6 1 0.1313 0.0633 4.30 0.0381

VD*mdcd_grp1 1 5 1 0.00700 0.0445 0.02 0.8750

VD*mdcd_grp1 1 4 1 -0.1684 0.0377 19.90 <.0001

VD*mdcd_grp1 1 3 1 -0.0115 0.0276 0.17 0.6768

VD*mdcd_grp1 1 2 1 -0.0672 0.0262 6.56 0.0104

VD*mdcd_grp1 1 1 1 0.0521 0.0574 0.82 0.3640

VD*stdy_grp_5000 1 1 1 -0.0555 0.0242 5.28 0.0216

age_grp*gndr 4 1 1 0.0125 0.0448 0.08 0.7797

age_grp*gndr 3 1 1 -0.0332 0.0388 0.73 0.3924

age_grp*gndr 2 1 1 -0.0569 0.0212 7.19 0.0073

age_grp*gndr 1 1 1 -0.0122 0.0217 0.32 0.5733

age_grp*mdcd_grp1 4 6 1 0.5014 0.1613 9.66 0.0019

age_grp*mdcd_grp1 4 5 1 0.6673 0.1519 19.31 <.0001

age_grp*mdcd_grp1 4 4 1 0.3401 0.3790 0.81 0.3696

age_grp*mdcd_grp1 4 3 1 0.4351 0.1785 5.94 0.0148

age_grp*mdcd_grp1 4 2 1 0.4397 0.1456 9.13 0.0025

age_grp*mdcd_grp1 4 1 1 0.2073 6.0423 0.00 0.9726

age_grp*mdcd_grp1 3 6 1 -0.0907 0.1338 0.46 0.4979

age_grp*mdcd_grp1 3 5 1 0.1359 0.1040 1.71 0.1910

age_grp*mdcd_grp1 3 4 1 0.3182 0.2417 1.73 0.1879

age_grp*mdcd_grp1 3 3 1 -0.3962 0.1363 8.45 0.0037

age_grp*mdcd_grp1 3 2 1 0.1243 0.0955 1.69 0.1930

age_grp*mdcd_grp1 3 1 1 -0.0995 1.6708 0.00 0.9525

age_grp*mdcd_grp1 2 6 1 -0.0115 0.1284 0.01 0.9289

age_grp*mdcd_grp1 2 5 1 -0.1515 0.0848 3.19 0.0740

age_grp*mdcd_grp1 2 4 1 -0.1606 0.1228 1.71 0.1910

age_grp*mdcd_grp1 2 3 1 -0.0399 0.0706 0.32 0.5718

age_grp*mdcd_grp1 2 2 1 -0.2825 0.0573 24.31 <.0001

age_grp*mdcd_grp1 2 1 1 -0.0794 1.5294 0.00 0.9586

age_grp*mdcd_grp1 1 6 1 0.0537 0.1462 0.14 0.7132

age_grp*mdcd_grp1 1 5 1 -0.1420 0.0935 2.31 0.1287

age_grp*mdcd_grp1 1 4 1 -0.3201 0.1228 6.79 0.0091

age_grp*mdcd_grp1 1 3 1 -0.1085 0.0718 2.29 0.1305

age_grp*mdcd_grp1 1 2 1 -0.2193 0.0659 11.07 0.0009

age_grp*mdcd_grp1 1 1 1 -0.0191 1.5302 0.00 0.9900

age_grp*stdy_grp_5000 4 1 1 -0.1302 0.0537 5.89 0.0153

age_grp*stdy_grp_5000 3 1 1 -0.0768 0.0550 1.95 0.1625

age_grp*stdy_grp_5000 2 1 1 0.0506 0.0340 2.22 0.1362

age_grp*stdy_grp_5000 1 1 1 0.0236 0.0351 0.45 0.5013

gndr*mdcd_grp1 1 6 1 0.1715 0.0559 9.40 0.0022

gndr*mdcd_grp1 1 5 1 0.0340 0.0398 0.73 0.3937

gndr*mdcd_grp1 1 4 1 -0.00056 0.0347 0.00 0.9870

gndr*mdcd_grp1 1 3 1 0.0129 0.0244 0.28 0.5967

gndr*mdcd_grp1 1 2 1 -0.0407 0.0227 3.20 0.0736

gndr*mdcd_grp1 1 1 1 -0.0772 0.0476 2.63 0.1046

age*age 1 -0.00118 0.000093 162.24 <.0001

age*stdy_prd 1 -0.00063 0.000122 26.51 <.0001

stdy_prd*stdy_prd 1 -0.00039 0.000185 4.46 0.0348

Part e – Model parameters for major bleeding associated high risk AF

Standard Wald

Parameter Levels DF Estimate Error Chi-Square Pr > ChiSq

Intercept 1 -5.1528 0.6298 66.94 <.0001

ANEMIA 1 1 0.1369 0.0174 62.00 <.0001

ASTHMA 1 1 0.0652 0.0105 38.55 <.0001

CAD 1 1 0.0612 0.0136 20.38 <.0001

COGI 1 1 0.1390 0.0272 26.18 <.0001

DEP 1 1 0.0831 0.0167 24.60 <.0001

LIVD 1 1 0.1054 0.0216 23.90 <.0001

MBldg 1 1 0.5488 0.0285 372.08 <.0001

age 1 0.0689 0.00674 104.46 <.0001

age_grp 4 1 0.6011 1.3722 0.19 0.6614

age_grp 3 1 -0.6750 1.3680 0.24 0.6217

age_grp 2 1 0.00376 0.0467 0.01 0.9359

age_grp 1 1 -0.0828 0.0564 2.16 0.1418

stdy_grp_5000 1 1 0.3984 0.0302 174.54 <.0001

stdy_prd 1 0.0857 0.0186 21.11 <.0001

ANEMIA*ASTHMA 1 1 1 -0.0276 0.00983 7.87 0.0050

ANEMIA*CKD 1 1 1 0.0283 0.0134 4.47 0.0346

ANEMIA*LIPDIS 1 1 1 -0.0474 0.00786 36.36 <.0001

ANEMIA*OSTEO 1 1 1 -0.0323 0.00924 12.23 0.0005

ANEMIA*gndr 1 1 1 -0.0183 0.00829 4.88 0.0271

ANEMIA*mdcd_grp1 1 6 1 0.0577 0.0447 1.66 0.1973

ANEMIA*mdcd_grp1 1 5 1 0.0299 0.0346 0.75 0.3876

ANEMIA*mdcd_grp1 1 4 1 0.0452 0.0249 3.29 0.0698

ANEMIA*mdcd_grp1 1 3 1 0.0410 0.0198 4.30 0.0381

ANEMIA*mdcd_grp1 1 2 1 0.000266 0.0184 0.00 0.9885

ANEMIA*mdcd_grp1 1 1 1 -0.1282 0.0351 13.37 0.0003

ASTHMA*COPD 1 1 1 -0.0207 0.00897 5.33 0.0210

CAD*VD 1 1 1 -0.0508 0.0128 15.63 <.0001

CHF*HYP 1 1 1 0.0648 0.0174 13.83 0.0002

CHF*age_grp 1 4 1 -0.1044 0.0497 4.41 0.0357

CHF*age_grp 1 3 1 0.0838 0.0452 3.44 0.0635

CHF*age_grp 1 2 1 -0.0119 0.0264 0.20 0.6534

CHF*age_grp 1 1 1 -0.0335 0.0276 1.48 0.2241

CHF*mdcd_grp1 1 6 1 -0.0589 0.0577 1.04 0.3077

CHF*mdcd_grp1 1 5 1 0.0381 0.0476 0.64 0.4236

CHF*mdcd_grp1 1 4 1 -0.0281 0.0380 0.55 0.4593

CHF*mdcd_grp1 1 3 1 0.1013 0.0299 11.48 0.0007

CHF*mdcd_grp1 1 2 1 -0.0409 0.0276 2.19 0.1388

CHF*mdcd_grp1 1 1 1 0.0975 0.0761 1.64 0.1998

CKD*COPD 1 1 1 -0.0413 0.0119 12.09 0.0005

CKD*LIVD 1 1 1 -0.0390 0.0145 7.25 0.0071

CKD*MBldg 1 1 1 -0.0654 0.0151 18.71 <.0001

CKD*SAPN 1 1 1 -0.0414 0.0141 8.60 0.0034

CKD*SPOND 1 1 1 -0.0390 0.0111 12.40 0.0004

COGI*HYP 1 1 1 -0.0480 0.0215 4.99 0.0255

COPD*LIPDIS 1 1 1 -0.0221 0.00824 7.19 0.0073

COPD*OSTEO 1 1 1 -0.0290 0.00908 10.21 0.0014

COPD*STROKE 1 1 1 -0.0314 0.0120 6.87 0.0088

DEP*MBldg 1 1 1 -0.0263 0.00917 8.21 0.0042

DEP*age_grp 1 4 1 0.0216 0.0473 0.21 0.6478

DEP*age_grp 1 3 1 -0.0464 0.0402 1.33 0.2494

DEP*age_grp 1 2 1 -0.0450 0.0204 4.84 0.0278

DEP*age_grp 1 1 1 -0.0169 0.0191 0.79 0.3751

DM*SAPN 1 1 1 -0.0403 0.00944 18.23 <.0001

DM*gndr 1 1 1 0.0257 0.00913 7.91 0.0049

HTHD*STROKE 1 1 1 -0.0875 0.0160 30.11 <.0001

HYP*LIPDIS 1 1 1 -0.0158 0.00749 4.46 0.0346

HYP*OSTEO 1 1 1 -0.0239 0.00897 7.12 0.0076

HYP*SPOND 1 1 1 -0.0178 0.00686 6.76 0.0093

HYP*age_grp 1 4 1 0.0588 0.0433 1.84 0.1748

HYP*age_grp 1 3 1 0.0281 0.0363 0.60 0.4394

HYP*age_grp 1 2 1 -0.0630 0.0200 9.94 0.0016

HYP*age_grp 1 1 1 -0.0396 0.0194 4.17 0.0411

HYP*mdcd_grp1 1 6 1 -0.0606 0.0423 2.05 0.1521

HYP*mdcd_grp1 1 5 1 -0.0126 0.0319 0.16 0.6929

HYP*mdcd_grp1 1 4 1 0.0188 0.0261 0.52 0.4717

HYP*mdcd_grp1 1 3 1 0.0217 0.0189 1.31 0.2518

HYP*mdcd_grp1 1 2 1 0.0468 0.0178 6.93 0.0085

HYP*mdcd_grp1 1 1 1 0.00580 0.0312 0.03 0.8526

HYP*stdy_grp_5000 1 1 1 -0.0767 0.0213 12.91 0.0003

LIPDIS*LIVD 1 1 1 -0.0309 0.00849 13.28 0.0003

LIPDIS*MS 1 1 1 0.0587 0.0163 12.90 0.0003

LIPDIS*STROKE 1 1 1 -0.0290 0.0135 4.64 0.0313

LIPDIS*gndr 1 1 1 0.0217 0.00698 9.68 0.0019

LIPDIS*mdcd_grp1 1 6 1 0.0740 0.0399 3.44 0.0638

LIPDIS*mdcd_grp1 1 5 1 -0.0156 0.0306 0.26 0.6110

LIPDIS*mdcd_grp1 1 4 1 -0.0237 0.0236 1.01 0.3160

LIPDIS*mdcd_grp1 1 3 1 -0.0526 0.0179 8.61 0.0033

LIPDIS*mdcd_grp1 1 2 1 0.0198 0.0173 1.30 0.2534

LIPDIS*mdcd_grp1 1 1 1 -0.0230 0.0308 0.56 0.4551

LIVD*SPOND 1 1 1 -0.0299 0.00819 13.30 0.0003

LIVD*STROKE 1 1 1 -0.0556 0.0158 12.38 0.0004

LIVD*VALVD 1 1 1 -0.0346 0.0140 6.12 0.0134

MBldg*SPOND 1 1 1 -0.0434 0.00824 27.76 <.0001

MBldg*VD 1 1 1 -0.0336 0.0123 7.45 0.0063

MBldg*age_grp 1 4 1 -0.0650 0.0509 1.63 0.2015

MBldg*age_grp 1 3 1 -0.0325 0.0408 0.64 0.4250

MBldg*age_grp 1 2 1 0.00818 0.0226 0.13 0.7172

MBldg*age_grp 1 1 1 -0.0196 0.0221 0.78 0.3756

MBldg*mdcd_grp1 1 6 1 0.4636 0.0498 86.81 <.0001

MBldg*mdcd_grp1 1 5 1 -0.0672 0.0377 3.17 0.0751

MBldg*mdcd_grp1 1 4 1 -0.1022 0.0287 12.72 0.0004

MBldg*mdcd_grp1 1 3 1 -0.0835 0.0216 14.99 0.0001

MBldg*mdcd_grp1 1 2 1 -0.0468 0.0198 5.55 0.0185

MBldg*mdcd_grp1 1 1 1 -0.0461 0.0371 1.54 0.2142

MBldg*stdy_grp_5000 1 1 1 -0.0910 0.0239 14.47 0.0001

MS*gndr 1 1 1 0.0337 0.0141 5.74 0.0166

OSTEO*mdcd_grp1 1 6 1 -0.0192 0.0442 0.19 0.6637

OSTEO*mdcd_grp1 1 5 1 0.0655 0.0323 4.11 0.0427

OSTEO*mdcd_grp1 1 4 1 0.0322 0.0256 1.58 0.2087

OSTEO*mdcd_grp1 1 3 1 0.0145 0.0195 0.55 0.4564

OSTEO*mdcd_grp1 1 2 1 0.0261 0.0186 1.96 0.1611

OSTEO*mdcd_grp1 1 1 1 -0.1070 0.0388 7.62 0.0058

OSTEO*stdy_grp_5000 1 1 1 -0.0467 0.0132 12.42 0.0004

SPOND*age_grp 1 4 1 -0.1249 0.0365 11.72 0.0006

SPOND*age_grp 1 3 1 -0.0445 0.0315 1.99 0.1584

SPOND*age_grp 1 2 1 0.0287 0.0158 3.32 0.0684

SPOND*age_grp 1 1 1 0.0488 0.0147 11.08 0.0009

STROKE*VALVD 1 1 1 -0.0452 0.0142 10.16 0.0014

STROKE*gndr 1 1 1 0.0287 0.0132 4.72 0.0298

VALVD*VD 1 1 1 -0.0388 0.0127 9.35 0.0022

age_grp*mdcd_grp1 4 6 1 -0.5275 1.3697 0.15 0.7001

age_grp*mdcd_grp1 4 5 1 -0.4623 1.3698 0.11 0.7358

age_grp*mdcd_grp1 4 4 1 -1.0485 1.3873 0.57 0.4498

age_grp*mdcd_grp1 4 3 1 -0.9143 1.3711 0.44 0.5049

age_grp*mdcd_grp1 4 2 1 -0.4624 1.3692 0.11 0.7355

age_grp*mdcd_grp1 4 1 1 3.0305 8.1930 0.14 0.7115

age_grp*mdcd_grp1 3 6 1 0.6049 1.3681 0.20 0.6584

age_grp*mdcd_grp1 3 5 1 0.5959 1.3676 0.19 0.6631

age_grp*mdcd_grp1 3 4 1 0.6239 1.3797 0.20 0.6511

age_grp*mdcd_grp1 3 3 1 0.7067 1.3683 0.27 0.6055

age_grp*mdcd_grp1 3 2 1 0.7199 1.3675 0.28 0.5986

age_grp*mdcd_grp1 3 1 1 -3.1379 8.1867 0.15 0.7015

age_grp*mdcd_grp1 2 6 1 -0.0112 0.0800 0.02 0.8889

age_grp*mdcd_grp1 2 5 1 -0.1082 0.0585 3.42 0.0645

age_grp*mdcd_grp1 2 4 1 0.2336 0.0659 12.56 0.0004

age_grp*mdcd_grp1 2 3 1 0.1027 0.0436 5.57 0.0183

age_grp*mdcd_grp1 2 2 1 -0.1406 0.0394 12.73 0.0004

age_grp*mdcd_grp1 2 1 1 0.0407 0.1177 0.12 0.7297

age_grp*mdcd_grp1 1 6 1 -0.3070 0.1071 8.22 0.0041

age_grp*mdcd_grp1 1 5 1 -0.0491 0.0616 0.63 0.4258

age_grp*mdcd_grp1 1 4 1 0.1886 0.0654 8.32 0.0039

age_grp*mdcd_grp1 1 3 1 0.1110 0.0437 6.47 0.0110

age_grp*mdcd_grp1 1 2 1 -0.00739 0.0411 0.03 0.8572

age_grp*mdcd_grp1 1 1 1 0.1055 0.1108 0.91 0.3408

mdcd_grp1*stdy_grp_5000 6 1 1 0.1357 0.0495 7.52 0.0061

mdcd_grp1*stdy_grp_5000 5 1 1 -0.00412 0.0493 0.01 0.9334

mdcd_grp1*stdy_grp_5000 4 1 1 -0.1105 0.0529 4.36 0.0367

mdcd_grp1*stdy_grp_5000 3 1 1 -0.1734 0.0375 21.33 <.0001

mdcd_grp1*stdy_grp_5000 2 1 1 0.00288 0.0327 0.01 0.9299

mdcd_grp1*stdy_grp_5000 1 1 1 0.0357 0.0771 0.21 0.6432

age*age 1 -0.00034 0.000061 30.80 <.0001

age*stdy_prd 1 -0.00055 0.000070 62.08 <.0001

stdy_prd*stdy_prd 1 -0.00041 0.000143 8.23 0.0041

Part f – Model parameters for cognitive impairment associated high risk AF

Standard Wald

Parameter Levels DF Estimate Error Chi-Square Pr > ChiSq

Intercept 1 -9.4075 1.2321 58.29 <.0001

COGI 1 1 1.1106 0.0520 456.77 <.0001

age 1 0.0675 0.00944 51.21 <.0001

mdcd_grp1 6 1 0.6025 0.1117 29.08 <.0001

mdcd_grp1 5 1 0.0679 0.1133 0.36 0.5489

mdcd_grp1 4 1 0.6978 0.1298 28.90 <.0001

mdcd_grp1 3 1 -0.1125 0.1068 1.11 0.2921

mdcd_grp1 2 1 0.1903 0.0924 4.24 0.0394

mdcd_grp1 1 1 -0.3427 2.3305 0.02 0.8831

stdy_prd 1 0.1512 0.0353 18.35 <.0001

ANEMIA*CKD 1 1 1 -0.0856 0.0215 15.79 <.0001

ANEMIA*COGI 1 1 1 -0.0853 0.0217 15.40 <.0001

ANEMIA*age_grp 1 4 1 -0.0848 0.0447 3.59 0.0580

ANEMIA*age_grp 1 3 1 -0.0902 0.0475 3.61 0.0575

ANEMIA*age_grp 1 2 1 0.0613 0.0321 3.64 0.0564

ANEMIA*age_grp 1 1 1 0.0958 0.0360 7.08 0.0078

ANEMIA*gndr 1 1 1 -0.0375 0.0180 4.37 0.0365

ANEMIA*mdcd_grp1 1 6 1 0.1819 0.0554 10.78 0.0010

ANEMIA*mdcd_grp1 1 5 1 0.0581 0.0614 0.89 0.3442

ANEMIA*mdcd_grp1 1 4 1 0.1075 0.0458 5.51 0.0189

ANEMIA*mdcd_grp1 1 3 1 -0.0252 0.0422 0.36 0.5500

ANEMIA*mdcd_grp1 1 2 1 -0.0303 0.0341 0.79 0.3745

ANEMIA*mdcd_grp1 1 1 1 -0.2166 0.0925 5.48 0.0192

ASTHMA*HTHD 1 1 1 0.0572 0.0238 5.79 0.0161

ASTHMA*SPOND 1 1 1 0.0717 0.0229 9.78 0.0018

CAD*COGI 1 1 1 -0.0558 0.0239 5.47 0.0194

CHF*COGI 1 1 1 -0.0802 0.0339 5.59 0.0180

CHF*MS 1 1 1 0.1492 0.0330 20.48 <.0001

COGI*COPD 1 1 1 -0.0699 0.0187 13.90 0.0002

COGI*DEP 1 1 1 -0.1038 0.0220 22.26 <.0001

COGI*HYP 1 1 1 -0.0618 0.0191 10.42 0.0012

COGI*LIPDIS 1 1 1 -0.0681 0.0168 16.38 <.0001

COGI*LIVD 1 1 1 -0.0766 0.0269 8.11 0.0044

COGI*SPOND 1 1 1 -0.0956 0.0219 19.02 <.0001

COGI*STROKE 1 1 1 -0.1743 0.0279 39.12 <.0001

COGI*age_grp 1 4 1 -0.2537 0.0461 30.31 <.0001

COGI*age_grp 1 3 1 0.0190 0.0496 0.15 0.7011

COGI*age_grp 1 2 1 -0.0783 0.0384 4.14 0.0418

COGI*age_grp 1 1 1 0.0457 0.0456 1.00 0.3164

COGI*mdcd_grp1 1 6 1 0.3136 0.0586 28.61 <.0001

COGI*mdcd_grp1 1 5 1 0.0451 0.0718 0.39 0.5300

COGI*mdcd_grp1 1 4 1 -0.0727 0.0610 1.42 0.2337

COGI*mdcd_grp1 1 3 1 -0.1348 0.0556 5.88 0.0153

COGI*mdcd_grp1 1 2 1 -0.0725 0.0471 2.36 0.1241

COGI*mdcd_grp1 1 1 1 -0.1831 0.1584 1.34 0.2475

DEP*SAPN 1 1 1 -0.1085 0.0315 11.88 0.0006

DEP*gndr 1 1 1 -0.0408 0.0168 5.87 0.0154

DEP*mdcd_grp1 1 6 1 -0.00875 0.0489 0.03 0.8580

DEP*mdcd_grp1 1 5 1 -0.0270 0.0580 0.22 0.6410

DEP*mdcd_grp1 1 4 1 -0.0905 0.0431 4.41 0.0357

DEP*mdcd_grp1 1 3 1 -0.1099 0.0394 7.80 0.0052

DEP*mdcd_grp1 1 2 1 0.0626 0.0334 3.52 0.0605

DEP*mdcd_grp1 1 1 1 0.0697 0.0933 0.56 0.4549

DEP*stdy_grp_5000 1 1 1 -0.1115 0.0297 14.11 0.0002

DM*VD 1 1 1 -0.0532 0.0196 7.38 0.0066

DM*mdcd_grp1 1 6 1 -0.1635 0.0500 10.68 0.0011

DM*mdcd_grp1 1 5 1 0.1332 0.0575 5.37 0.0204

DM*mdcd_grp1 1 4 1 -0.1154 0.0485 5.66 0.0173

DM*mdcd_grp1 1 3 1 -0.0282 0.0431 0.43 0.5127

DM*mdcd_grp1 1 2 1 0.00854 0.0337 0.06 0.7998

DM*mdcd_grp1 1 1 1 0.1498 0.0901 2.76 0.0966

HTHD*age_grp 1 4 1 0.1214 0.1673 0.53 0.4682

HTHD*age_grp 1 3 1 0.3839 0.1408 7.43 0.0064

HTHD*age_grp 1 2 1 -0.0497 0.0796 0.39 0.5329

HTHD*age_grp 1 1 1 -0.1331 0.0878 2.30 0.1296

HTHD*stdy_grp_5000 1 1 1 -0.1397 0.0405 11.90 0.0006

HYP*gndr 1 1 1 -0.0336 0.0165 4.17 0.0411

HYP*mdcd_grp1 1 6 1 -0.1063 0.0354 9.02 0.0027

HYP*mdcd_grp1 1 5 1 -0.0930 0.0570 2.66 0.1027

HYP*mdcd_grp1 1 4 1 -0.0247 0.0491 0.25 0.6151

HYP*mdcd_grp1 1 3 1 -0.0273 0.0406 0.45 0.5003

HYP*mdcd_grp1 1 2 1 0.0285 0.0341 0.70 0.4023

HYP*mdcd_grp1 1 1 1 0.1049 0.0831 1.59 0.2070

LIVD*stdy_grp_5000 1 1 1 -0.0648 0.0266 5.95 0.0147

MBldg*age_grp 1 4 1 -0.0657 0.0677 0.94 0.3319

MBldg*age_grp 1 3 1 -0.1975 0.0714 7.66 0.0056

MBldg*age_grp 1 2 1 -0.0124 0.0426 0.08 0.7717

MBldg*age_grp 1 1 1 0.1284 0.0427 9.03 0.0027

MS*gndr 1 1 1 0.0821 0.0206 15.87 <.0001

SAPN*SPOND 1 1 1 -0.0604 0.0279 4.69 0.0303

SAPN*VD 1 1 1 -0.0680 0.0223 9.30 0.0023

SPOND*mdcd_grp1 1 6 1 -0.1033 0.0499 4.28 0.0386

SPOND*mdcd_grp1 1 5 1 0.0584 0.0511 1.31 0.2532

SPOND*mdcd_grp1 1 4 1 -0.1840 0.0424 18.87 <.0001

SPOND*mdcd_grp1 1 3 1 0.00748 0.0372 0.04 0.8407

SPOND*mdcd_grp1 1 2 1 -0.0190 0.0317 0.36 0.5484

SPOND*mdcd_grp1 1 1 1 0.1365 0.0829 2.71 0.0998

STROKE*age_grp 1 4 1 -0.1901 0.0559 11.58 0.0007

STROKE*age_grp 1 3 1 -0.0297 0.0602 0.24 0.6216

STROKE*age_grp 1 2 1 0.0224 0.0401 0.31 0.5760

STROKE*age_grp 1 1 1 0.0935 0.0447 4.38 0.0363

STROKE*stdy_grp_5000 1 1 1 -0.1228 0.0282 18.92 <.0001

age_grp*gndr 4 1 1 0.0651 0.0346 3.54 0.0597

age_grp*gndr 3 1 1 0.0200 0.0376 0.28 0.5937

age_grp*gndr 2 1 1 0.0558 0.0273 4.16 0.0413

age_grp*gndr 1 1 1 -0.00507 0.0298 0.03 0.8649

age_grp*mdcd_grp1 4 6 1 -0.2272 0.2098 1.17 0.2790

age_grp*mdcd_grp1 4 5 1 -0.0400 0.2160 0.03 0.8532

age_grp*mdcd_grp1 4 4 1 0.0315 0.3155 0.01 0.9205

age_grp*mdcd_grp1 4 3 1 -0.1744 0.2285 0.58 0.4455

age_grp*mdcd_grp1 4 2 1 0.3386 0.2014 2.83 0.0927

age_grp*mdcd_grp1 4 1 1 3.4222 9.1884 0.14 0.7096

age_grp*mdcd_grp1 3 6 1 -0.1264 0.1903 0.44 0.5065

age_grp*mdcd_grp1 3 5 1 0.1553 0.1935 0.64 0.4222

age_grp*mdcd_grp1 3 4 1 0.3043 0.2700 1.27 0.2598

age_grp*mdcd_grp1 3 3 1 0.0262 0.2041 0.02 0.8980

age_grp*mdcd_grp1 3 2 1 0.5920 0.1768 11.21 0.0008

age_grp*mdcd_grp1 3 1 1 -2.9356 2.5483 1.33 0.2493

age_grp*mdcd_grp1 2 6 1 0.1022 0.1202 0.72 0.3955

age_grp*mdcd_grp1 2 5 1 -0.2272 0.1264 3.23 0.0723

age_grp*mdcd_grp1 2 4 1 -0.1460 0.1243 1.38 0.2400

age_grp*mdcd_grp1 2 3 1 0.0568 0.0994 0.33 0.5677

age_grp*mdcd_grp1 2 2 1 -0.2629 0.0864 9.27 0.0023

age_grp*mdcd_grp1 2 1 1 0.0267 2.3261 0.00 0.9908

age_grp*mdcd_grp1 1 6 1 0.1462 0.1538 0.90 0.3417

age_grp*mdcd_grp1 1 5 1 -0.0247 0.1427 0.03 0.8627

age_grp*mdcd_grp1 1 4 1 -0.0229 0.1251 0.03 0.8549

age_grp*mdcd_grp1 1 3 1 -0.0811 0.1033 0.62 0.4326

age_grp*mdcd_grp1 1 2 1 -0.1969 0.0964 4.17 0.0410

age_grp*mdcd_grp1 1 1 1 -0.3567 2.3275 0.02 0.8782

age_grp*stdy_grp_5000 4 1 1 -0.1029 0.0574 3.22 0.0729

age_grp*stdy_grp_5000 3 1 1 -0.1568 0.0743 4.45 0.0349

age_grp*stdy_grp_5000 2 1 1 0.1337 0.0549 5.94 0.0148

age_grp*stdy_grp_5000 1 1 1 0.0506 0.0680 0.55 0.4568

age*stdy_prd 1 -0.00051 0.000136 14.21 0.0002

stdy_prd*stdy_prd 1 -0.00103 0.000267 14.99 0.0001

***Note:***

CHF – congestive heart failure

HYP – hypertension

DM – diabetes mellitus

STROKE – includes ischemic stroke, transient ischemic attack, thrombo-embolic event

VD – vascular disease (myocardial infarction, peripheral artery disease)

CAD – coronary artery disease

VALVD - valvular disease

SAPN – chronic sleep apnea

CKD – chronic kidney disease

COPD – chronic obstructive pulmonary disease/bronchiectasis

MBldg – major bleeding (e.g., intracranial and gastro-intestinal)

CogI – cognitive impairment

LIVD – liver disease

DEP – depression

LIPDIS - lipid disorders

SPOND – spondylosis/intervertebral discs,

OSTEO - osteoarthritis

HTHD – hyperthyroidism

MS – metabolic syndrome

Cost_threshold – high risk was considered when the total annual cost prior to the index date for AF or the equivalent date for the non-AF case

GNDR – gender

Age in years

AGE_GRP – age group (18-44 or 0; 45-54 or 1; 55-64 or 2; 65-74 or 3; 75-90 or 4)

Mdcd_grp1 – Medicaid groups (TANF or 0; family care or 1; two groups or 2; ABD nondual or 3; LTSS nondual or 4; ABD duals or 5; LTSS duals or 6)

Std_prd – length of enrollment in study (in months)

stdy_grp_5000 – a case is considered high risk if the total annual cost prior to the index date for the AF case or 0 otherwise


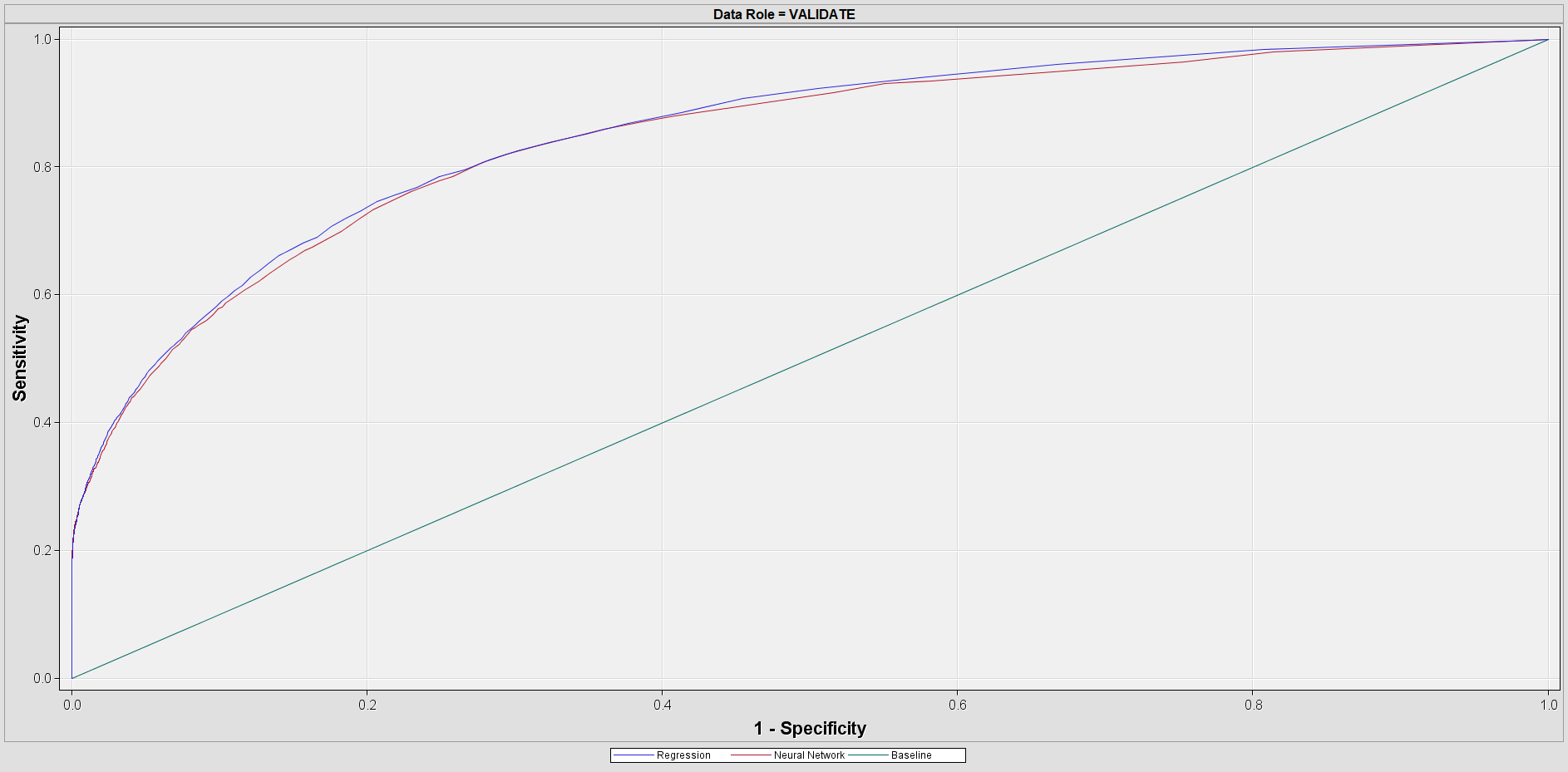


**Fig S1** Validation of ML models for AF incident outcome with cost threshold as a model feature “> $2000 for any case” (logistic regression – c index 0.851 95% CI 0.816-0.886; neural network – c index 0.844 95% CI 0.803-0.88)

| 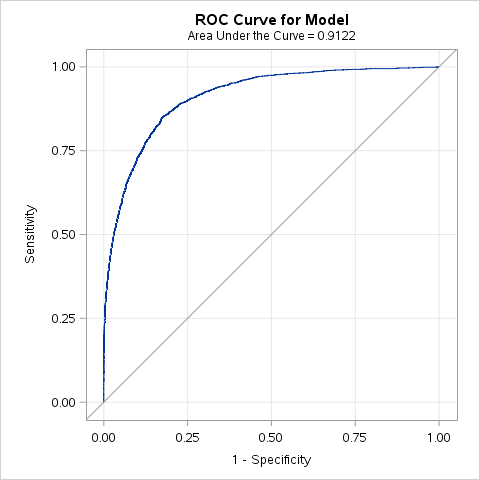  a | 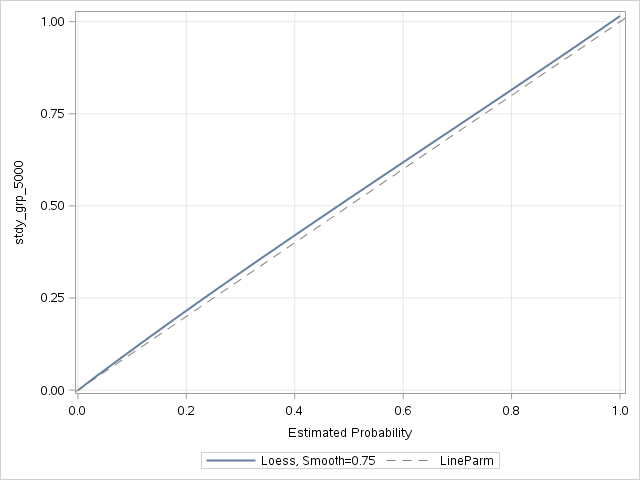  b |
| --- | --- |
| 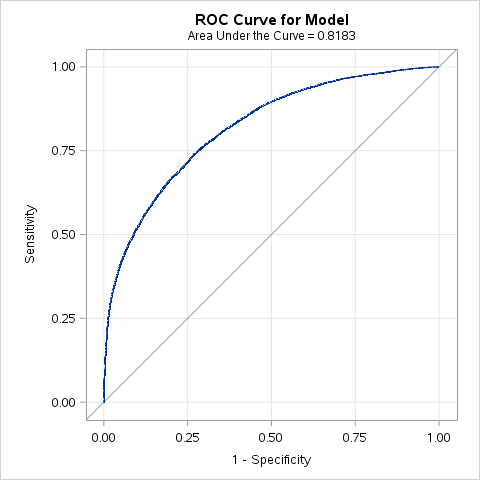  c | 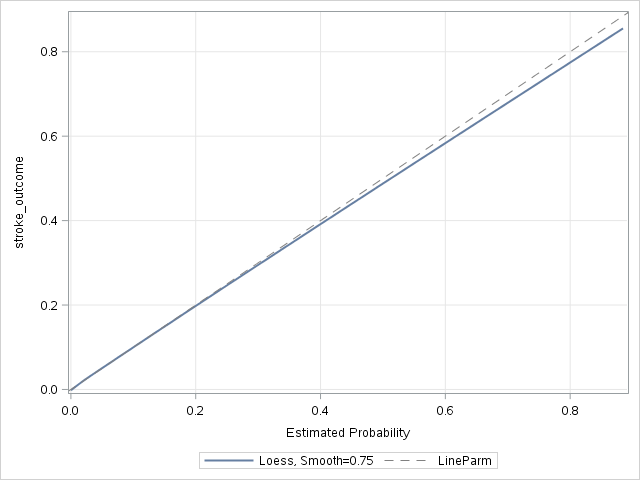  d |
| 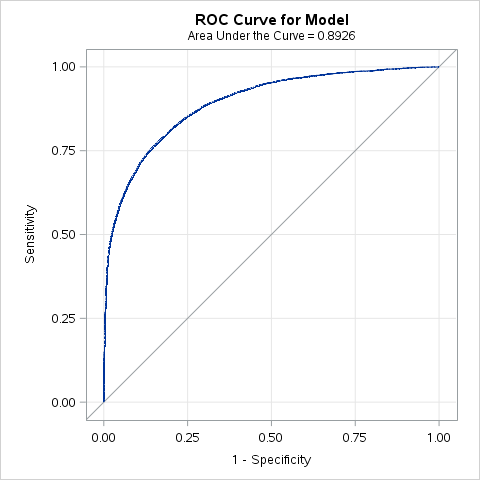  e | 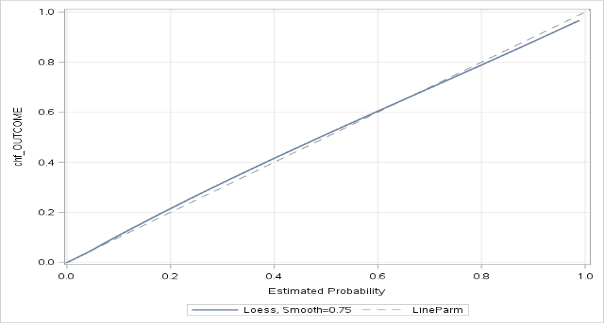  f |
| 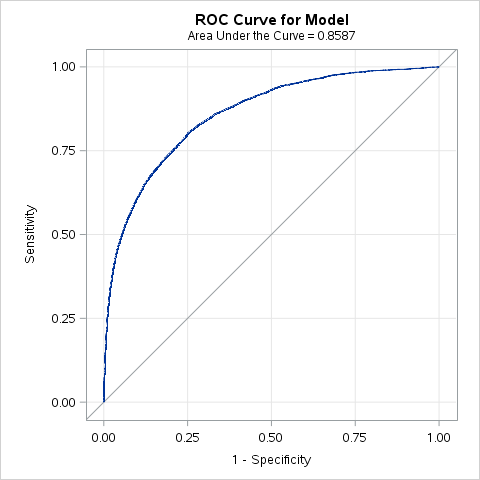  g | 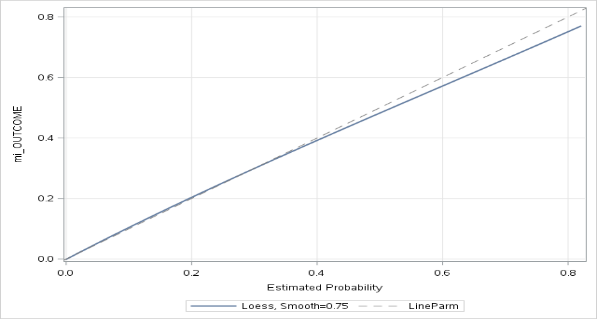  h |
| 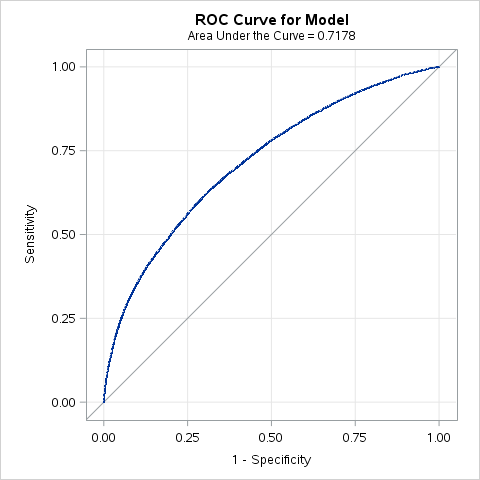  i | 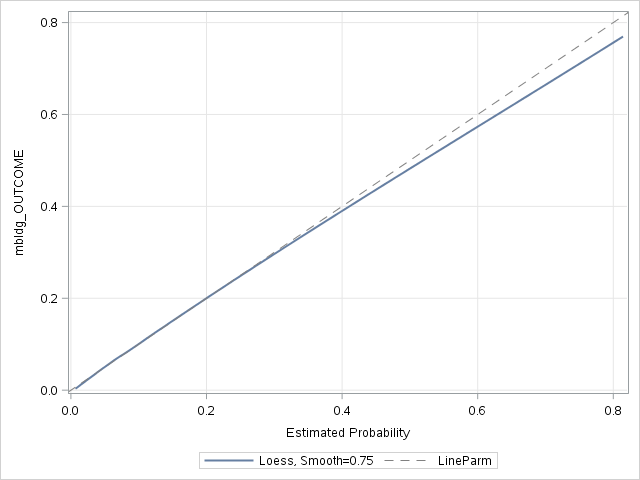  j |
| 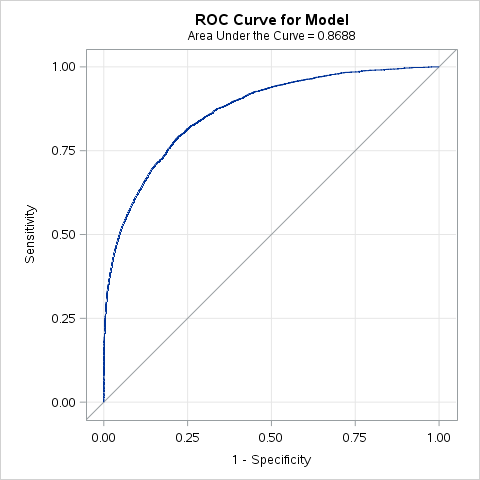  k | 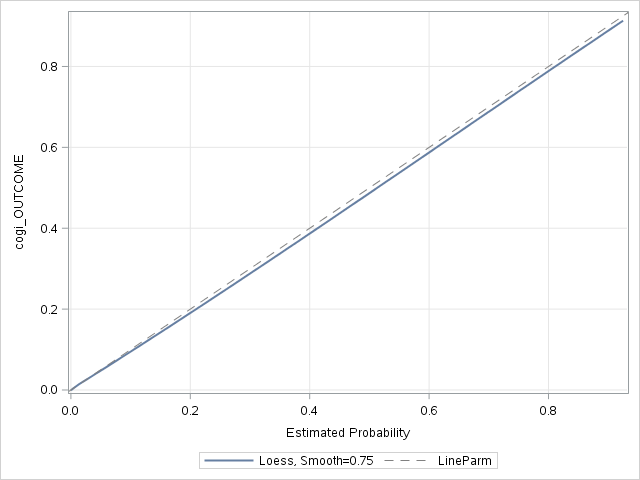  l |

**Fig S2** Discriminant validity and calibration of the atrial fibrillation (a/b), and associated stroke (c/d), heart failure (e/f), myocardial infarction (g/h), major bleeding (i/j) and cognitive impairment outcomes using the ML logistic regression based models (developed on training samples) using validation samples. AF was defined as having a condition as defined by an ICD10 code and further subjected to a cost threshold of total annual care cost of $5000 or more in the year prior to the index date for an AF case or 0 otherwise

**Appendix S1** Details of quantitative analyses

Descriptive analyses were performed to characterize the Medicaid population and its sub-cohorts for demographic variables (age as continuous and categorical representations; gender), comorbid history, and incidence rates and ratios. Descriptive statistics were calculated in numbers and % for categorical variables and mean (standard deviation or SD) for continuous variables. Main effect modeling was performed using the stepwise procedures and keeping variables in the model if they satisfy the 5% significance level. All parameters were estimated using the maximum likelihood computations.

Given the limitations of using administrative databases with regard to the severity of comorbid conditions as well as clinical outcomes, this was partly addressed via the use of a cost threshold with the assumption that conditions exceeding the threshold are considered economically costlier conditions and hence more severe. Therefore, a binary variable was created with 1 representing the high cost (hence the severe cases) and 0 representing the lower cost (thus the mild cases). The cost was based on the total allowed amount (paid by the insurance company as well as the deduction paid by the patient in a given health plan).

The severity of conditions was assessed by defining the proxy for severity as part of the model features. A model feature was created as high risk if the total annual cost in the prior year is $2000 or more and 0 otherwise (i.e., < $2000). Main effect models were developed on the entire population for AF as an outcome as well as the complication outcomes associated with AF or non-AF case. Additionally an ML model was only developed on the basis of the entire data for AF outcome.

Finally, a set of six ML models were developed and validated using the logistic-based formulations consisting of main effects, interactive terms and polynomial representations. The six models included the following: (a) high risk AF defined in terms of both the presence of an AF condition based on ICD 10 code as well as a cost threshold of $5000 or more based on the total annual care cost 1 year prior to the index date and low risk consisted of AF cases with cost threshold < $5000; and (b) the five potential complications associated with the AF conditions including stroke, heart failure, myocardial infarction, major bleeding and cognitive impairment. The model training was performed on 2/3 of the data and the remaining 1/3 had the external validation in terms of discriminant validity (c index), cumulative lift, model calibration, and decision analysis in terms of net benefit (cases/100 patients).

The ML models were parametric based (i.e., logistic regression and neutral network) and the detailed algorithms were detailed elsewhere.^13-14^ The ML based logistic regression algorithm included main effects, interaction terms and polynomial effects, with the model selection based on the stepwise method. Several polynomial terms were included in the ML formulation. The neutral network used a multilayer perceptron which consists of a feedforward multilayer network architecture composed of several layers of neurons, input layer, output layer, and five hidden layers. Model validation for the ML models was based on discrimination validity, with the models trained on 67% of the data and the remaining 33% for external validation. In this respect, the training and validation samples were extracted at random. Discriminant validity was assessed using C-indexes (area under the curve) for both the training and validation samples, separately.
